# Translational and Therapeutic Evaluation of RAS-GTP Inhibition by RMC-6236 in RAS-Driven Cancers

**DOI:** 10.1158/2159-8290.CD-24-0027

**Published:** 2024-04-09

**Authors:** Jingjing Jiang, Lingyan Jiang, Benjamin J. Maldonato, Yingyun Wang, Matthew Holderfield, Ida Aronchik, Ian P. Winters, Zeena Salman, Cristina Blaj, Marie Menard, Jens Brodbeck, Zhe Chen, Xing Wei, Michael J. Rosen, Yevgeniy Gindin, Bianca J. Lee, James W. Evans, Stephanie Chang, Zhican Wang, Kyle J. Seamon, Dylan Parsons, James Cregg, Abby Marquez, Aidan C.A. Tomlinson, Jason K. Yano, John E. Knox, Elsa Quintana, Andrew J. Aguirre, Kathryn C. Arbour, Abby Reed, W. Clay Gustafson, Adrian L. Gill, Elena S. Koltun, David Wildes, Jacqueline A.M. Smith, Zhengping Wang, Mallika Singh

**Affiliations:** 1 Revolution Medicines, Inc., Redwood City, California.; 2 D2G Oncology, Inc., Mountain View, California.; 3 Department of Medical Oncology, Dana-Farber Cancer Institute, Boston, Massachusetts.; 4 Harvard Medical School, Boston, Massachusetts.; 5 Broad Institute of MIT and Harvard, Cambridge, Massachusetts.; 6 Department of Medicine, Brigham and Women's Hospital, Boston, Massachusetts.; 7 Department of Medicine, Memorial Sloan Kettering Cancer Center, New York, New York.; 8 The Christ Hospital Cancer Center, Cincinnati, Ohio.

## Abstract

RAS-driven cancers comprise up to 30% of human cancers. RMC-6236 is a RAS(ON) multi-selective noncovalent inhibitor of the active, GTP-bound state of both mutant and wild-type variants of canonical RAS isoforms with broad therapeutic potential for the aforementioned unmet medical need. RMC-6236 exhibited potent anticancer activity across RAS-addicted cell lines, particularly those harboring mutations at codon 12 of *KRAS*. Notably, oral administration of RMC-6236 was tolerated *in vivo* and drove profound tumor regressions across multiple tumor types in a mouse clinical trial with *KRAS^G12X^* xenograft models. Translational PK/efficacy and PK/PD modeling predicted that daily doses of 100 mg and 300 mg would achieve tumor control and objective responses, respectively, in patients with RAS-driven tumors. Consistent with this, we describe here objective responses in two patients (at 300 mg daily) with advanced *KRAS^G12X^* lung and pancreatic adenocarcinoma, respectively, demonstrating the initial activity of RMC-6236 in an ongoing phase I/Ib clinical trial (NCT05379985).

**Significance:**

The discovery of RMC-6236 enables the first-ever therapeutic evaluation of targeted and concurrent inhibition of canonical mutant and wild-type RAS-GTP in RAS-driven cancers. We demonstrate that broad-spectrum RAS-GTP inhibition is tolerable at exposures that induce profound tumor regressions in preclinical models of, and in patients with, such tumors.

## Teaser

Broad-spectrum inhibition of RAS-GTP with RMC-6236 is active and tolerated at exposures that induce profound tumor regressions in preclinical models of RAS-driven cancers and that can be associated with objective responses in patients with such cancers.

## INTRODUCTION

Oncogenic mutations in *RAS* (*KRAS*, *HRAS*, and *NRAS*) proto-oncogenes drive up to 30% of human cancers, accounting for more than 200,000 new cancer cases in the United States each year, most notably of non–small cell lung cancer (NSCLC), colorectal cancer, and pancreatic ductal adenocarcinoma (PDAC; refs. 1, 2). Most oncogenic RAS mutations are gain-of-function missense alterations at hotspot codons 12, 13, or 61 that result in an impairment of GTP hydrolysis and/or acceleration of GDP-to-GTP nucleotide exchange by these small GTPases, such that the normally tightly regulated cellular equilibrium of a RAS protein shifts predominantly toward the active, GTP-bound (RAS(ON)) state. This shift drives increased oncogenic flux via activation of downstream effectors and signaling pathways linked to cell proliferation and survival (3). Until the recent development of direct inhibitors of KRAS^G12C^, RAS was largely considered undruggable (4).

*KRAS* is most frequently mutated in PDAC (92% of patients), followed by colorectal cancer (49%) and NSCLC (29%; refs. 1, 2), predominantly at codon 12 in these three indications. *KRAS* mutations also occur in 9% of ovarian cancers and 12% of gastric adenocarcinomas (1, 2). The relative representation of oncogenic RAS variants is highly variable across cancer types, likely due to an interplay between allele-specific biochemical, structural, and signaling distinctions and tissue (indication)-specific properties (3, 5). KRAS glycine 12 mutant (*KRAS*^G12X^) NSCLC and PDAC are thought to be particularly addicted to oncogenic RAS signaling, exemplified by *Kras*^G12D^ inactivation studies in genetically engineered mouse models (6), pharmacologic inhibition of KRAS^G12D^ in preclinical models (7, 8), and most recently illustrated by the clinical activity of KRAS^G12C^ inhibitors in patients, leading to regulatory approvals for monotherapy in the treatment of patients with advanced *KRAS*^G12C^ mutant NSCLC (9, 10). In contrast, *KRAS* mutations in colorectal cancer typically require *APC* loss to drive frank adenocarcinoma (11), suggesting that RAS oncogenic signaling may act as a cooperative oncogenic driver in this case (12). In addition, colorectal cancer is characterized by strong adaptive feedback mechanisms in response to RAS pathway inhibition, mostly mediated via the EGF receptor (EGFR) (13, 14). This likely underlies the reduced and more heterogeneous response to inactive-state selective KRAS^G12C^ inhibitors that has been observed thus far in colorectal cancer as compared with that in NSCLC, and the significant combination benefit that has been observed for these inhibitors with anti-EGFR antibodies (15, 16).

KRAS^G12C^ mutant-selective inhibitors introduce an allele-specific covalent modification of the cysteine residue of the KRAS^G12C^ protein in the GDP-bound inactive [KRAS^G12C^(OFF)] state. However, KRAS^G12C^ inhibitors only cover a small fraction of all oncogenic RAS mutations, including the most prevalent codon 12 mutations described above, leaving a significant unmet medical need for inhibitors targeting most RAS alterations in cancer (17). Mutant-selective inhibitors of KRAS^G12D^ (18, 19) and pan-KRAS inhibitors (20) could potentially expand the therapeutic landscape beyond *KRAS*^G12C^ mutant cancers. However, most currently described inhibitors bind in the same pocket on mutant KRAS as the first-generation KRAS^G12C^ inhibitors and selectively target the inactive, GDP-bound state of KRAS. Recently described covalent inhibitors of KRAS^G12D^ are a notable exception, but at present, activity in tumor models *in vivo* has not been demonstrated (19). Furthermore, resistance to KRAS^G12C^(OFF) inhibitors inevitably and rapidly arises in most patients with numerous recurring mechanisms of escape, including the emergence of secondary RAS mutations, KRAS^G12C^ switch II binding pocket mutations, targeted amplification of the *KRAS*^G12C^ allele, and upstream receptor tyrosine kinase (RTK) activation, all of which can reactivate RAS signaling via increased levels of GTP-bound RAS (21–23). Although clinical data on more recent inactive-state inhibitors are not yet available, it is anticipated that these will also be vulnerable to RAS-GTP–driven mechanisms of resistance, which could potentially be addressed by concurrent inhibition of the active, GTP-bound state of RAS variants in tumors cells.

To address the high unmet medical need in RAS-dependent cancers and the variety of RAS mutations beyond *KRAS*^G12C^, we developed a series of active state-selective RAS-GTP inhibitors that target multiple RAS variants (RAS(ON) multi-selective tri-complex inhibitors; https://doi.org/10.1038/s41586-024-07205-6). These compounds are derived from sanglifehrin A, which binds the abundant immunophilin cyclophilin A (CypA, HUGO symbol PPIA) with high affinity (24) and is a member of a class of natural products that inspired a paradigm for inhibiting undruggable targets (25, 26). We previously described the discovery and comprehensive *in vitro* and *in vivo* characterization of a preclinical tool RAS(ON) multi-selective inhibitor, RMC-7977, which remodels the CypA surface to create a binary compound:CypA complex with high affinity and selectivity for the active, GTP-bound state of both mutant and wild-type variants (https://doi.org/10.1038/s41586-024-07205-6, https://doi.org/10.1101/2023.12.03.569791). The resulting noncovalent CypA:compound:RAS tri-complex sterically blocks RAS–effector interactions and disrupts downstream oncogenic signaling. Here, we describe the preclinical characterization and key observations driving the initial clinical translation of the investigational agent, RMC-6236, which is structurally related to RMC-7977 and shares a conserved binding site and binding mode in the tri-complex formed between RAS(ON) proteins and CypA. Both compounds have comparable *in vitro* and *in vivo* properties in preclinical models. Based on an overall attractive drug-like profile, RMC-6236 was advanced into clinical development and is undergoing evaluation in a phase I/Ib trial as a monotherapy in patients with previously treated, advanced solid tumors, including NSCLC, and PDAC, with *KRAS* glycine 12 mutant (*KRAS^G12X^*) genotypes (NCT05379985). Two case studies of patients treated in this trial are described herein, providing select examples of the clinical antitumor activity of RMC-6236.

## RESULTS

### RMC-6236 Is A Potent Noncovalent Inhibitor of the GTP-Bound State of Multiple RAS Variants *In Vitro*

**Figure 1.**
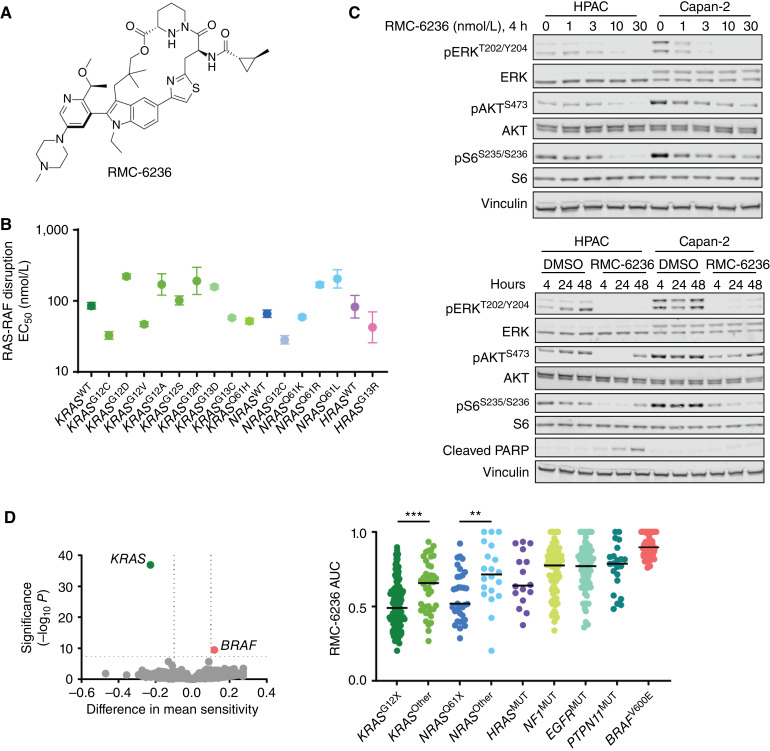
RMC-6236 is a potent noncovalent inhibitor of the GTP-bound state of multiple RAS variants *in vitro*. **A,** Chemical structure of RMC-6236. **B,** Biochemical potency of RMC-6236 for wild-type KRAS, NRAS, HRAS, and several oncogenic RAS variants. EC_50_ values shown for inhibition of RAS-RAF binding using recombinant proteins *in vitro*. Error bars indicate ± 95% CI. **C,** Immunoblot protein Western analyses of KRAS pathway targets in HPAC (*KRAS*^G12D/WT^, PDAC) and Capan-2 (*KRAS*^G12V/WT^, PDAC) cancer cells treated with RMC-6236 at the indicated concentrations and time points. **D,** RMC-6236 potency measured in the PRISM panel of cancer cell lines. Left, AUC difference between cell lines with and without a given gene mutation (x-axis) and the significance of the difference (y-axis). Points represent mutated genes. A negative AUC indicates increased sensitivity to RMC-6236 and positive AUC indicates resistance. Horizontal dashed line represents the *P*-value cutoff of 5 × 10^−8^. Vertical lines represent the absolute effect cutoff of 0.1. Right: AUC for KRAS mutant [glycine 12 depicted as *KRAS*^G12X^ (115 lines); all other KRAS mutations labeled *KRAS*^Other^ (42 lines)], *NRAS* mutant [glutamine 61 depicted as *NRAS*^Q61X^ (34 lines); all other NRAS mutations labeled as *NRAS*^Other^ (20 lines)], *HRAS* mutant, *NF1* mutant, *EGFR* mutant, *PTPN11* mutant, and *BRAF*^V600E^ mutant cell lines are shown. Comparison of indicated groups was done by the Wilcoxon rank-sum test with continuity correction. (**, *P* < 0.01; ***, *P* < 0.001).

The structure of RMC-6236, a tri-complex inhibitor developed using structure-guided design from sanglifehrin A, is shown in Fig. 1A (Supplementary Methods). A high-resolution cocrystal structure of RMC-6236 bound to CypA and GMPPNP-bound KRAS^G12D^ was solved (PDB Code: 9AX6), showing protein–protein interactions and protein–ligand interactions similar to those previously described (https://doi.org/10.1038/s41586-024-07205-6; Supplementary Fig. S1A). To characterize the steps of tri-complex formation, we first determined RMC-6236 affinity for CypA protein (K_D_1), which was 55.3 nmol/L (Supplementary Fig. S1B). Next, we evaluated the affinity of the RMC-6236:CypA binary complex for KRAS^G12V^, KRAS^G12D^, and KRAS^WT^ (K_D_2), which were 131, 364, and 154 nmol/L, respectively (Supplementary Fig. S1B).

We then measured the biochemical potency of RMC-6236 for RAS-RAF complex disruption *in vitro* using recombinant RAS variants, the RAS-binding domain of BRAF (RAF-RBD), and CypA proteins. Formation of the tri-complex with either wild-type KRAS, NRAS, or HRAS proteins potently disrupted RAF-RBD binding in a concentration-dependent manner, with EC_50_ values of 85, 66, and 82 nmol/L, respectively (Fig. 1B). Activity for oncogenic RAS mutant proteins was similar, ranging from 28 to 220 nmol/L. To explore the correlation between cellular and biochemical potencies of RMC-6236 for different RAS variants, cellular pERK inhibition potencies of RMC-6236 were investigated in a panel of matched mouse embryonic fibroblasts (MEF) null for all three *Ras* genes (RAS-less) where proliferation was restored with ectopic expression of WT or mutationally activated KRAS (Supplementary Fig. S1C; ref. 27). An excellent correlation was shown between the biochemical RAS-RAF disruption and cellular pERK inhibition potencies (*r*^2^ = 0.94). Cellular pERK inhibition data from representative cancer cell lines harboring different *RAS* mutant alleles treated with RMC-6236 (Supplementary Fig. S1D) were also in general agreement with the biochemical data, with *KRAS*^G12V^ mutant cell lines being most sensitive, notwithstanding the potential impact of other factors in a human cancer cell line that can influence apparent potency for inhibition of signaling. Furthermore, consistent with the biochemical activity observed against multiple RAS variants and cellular pERK inhibition potency in RAS mutant cell lines, RMC-6236 caused potent growth inhibition of *KRAS* mutant cancer cell lines, exemplified by HPAC (*KRAS*^G12D/WT^, PDAC) and Capan-2 (*KRAS*^G12V/WT^, PDAC) with EC_50_ at 1.2 and 1.4 nmol/L, respectively (Supplementary Fig. S1B).

Time- and concentration-dependent suppression of RAS pathway signaling markers, pERK, pAKT, and pS6, was observed in both HPAC and Capan-2 cell lines treated with RMC-6236, with sustained inhibition of pERK and pS6 up to at least 48 hours in Capan-2. HPAC cells also exhibited sustained pERK inhibition and time-dependent induction of apoptosis (Fig. 1C). We hypothesized that concurrent RAS inhibition by RMC-6236 would result in more sustained pathway inhibition as compared with that achieved by mutant-selective RAS inhibition wherein pathway rebound has been reported, driven by compensatory signaling through WT RAS variants (https://doi.org/10.1038/s41586-024-07205-6). When pERK levels were monitored in 2 *KRAS*^G12C^ cell lines [SW1463 (*KRAS*^G12C/G12C^, colorectal cancer) and NCI-H2030 (*KRAS*^G12C/G12C^, NSCLC)] up to 72 hours, KRAS^G12C^ mutant-selective inhibitor adagrasib treatment resulted in significant rebound of pERK signal over the course of 24 to 72 hours (Supplementary Fig. S1E). As predicted, substantially lower pERK recovery was observed when cells were treated with RMC-6236. These observations are consistent with a comparison between RAS(ON) multi-selective inhibitor RMC-7977 and the KRAS^G12D^-selective inhibitor MRTX1133 in 3 human cell lines described in Holderfield and colleagues (https://doi.org/10.1038/s41586-024-07205-6).

To identify genetic markers of response, RMC-6236 activity was assessed across a panel of 845 cancer cell lines (PRISM screen; Supplementary Table S1). Consistent with the mechanism of action, *KRAS* mutations were significantly correlated with sensitivity (*P* = 1.19 × 10^−37^, Wilcoxon rank-sum test with continuity correction), whereas *BRAF*^V600E^ mutations were associated with resistance (*P* = 6 × 10^−9^; Fig. 1D). Although *KRAS* mutation status was the most significant single genetic alteration associated with response, many *NRAS* mutant (*NRAS*^MUT^) cells and a subset of *HRAS*, *NF1*, *EGFR*, and *PTPN11* mutant cell lines also exhibited sensitivity to RMC-6236. Among *RAS* mutant cancer cell lines, *KRAS*^G12X^ and *NRAS* glutamine 61 mutant (*NRAS*^Q61X^) cells were significantly more sensitive compared with cell lines with other oncogenic *KRAS* or *NRAS* mutations, respectively (Wilcoxon rank-sum test with continuity correction, Fig. 1D). Consistent with the observation in the larger PRISM screen, RMC-6236 potently inhibited cell growth in *KRAS*^G12X^ and *NRAS*^Q61X^ cells with a median EC_50_ of 8 and 22 nmol/L, respectively, when tested in a smaller panel of *KRAS*^G12X^, *NRAS*^Q61X^, and *BRAF*^V600E^ mutant cancer cell lines, with *KRAS*^G12V^ lines being the most sensitive within the *KRAS*^G12X^ subset (Supplementary Fig. S1F; Supplementary Table S2). As predicted, all three *BRAF*^V600E^ mutant cells were resistant to RMC-6236 up to 100 nmol/L, consistent with the lack of dependence of *BRAF*^ClassI^ (A class I mutation at the V600 locus in the proto-oncogene encoding the BRAF serine/threonine-protein kinase) mutants upon upstream RAS signaling. Evidence of induction of apoptosis following direct RAS inhibition was also observed, with 40% of *KRAS*^G12X^ and *NRAS*^Q61X^ (28 out of 62 and 5 out of 13, respectively) cell lines exhibiting at least a 2-fold increase in caspase activation (Supplementary Fig. S1G). Sensitivity in *KRAS* mutant cells did not correlate with *KRAS* or *PPIA* (the gene encoding CypA) mRNA expression (Supplementary Fig. S1H and S1I), and copy numbers of either the *KRAS* mutant or WT allele did not significantly affect RMC-6236 sensitivity in the PRISM screen (Supplementary Table S1; Supplementary Fig. S1J).

### RMC-6236 Treatment Inhibits RAS Signaling and Drives Tumor Regressions *In Vivo*

**Figure 2.**
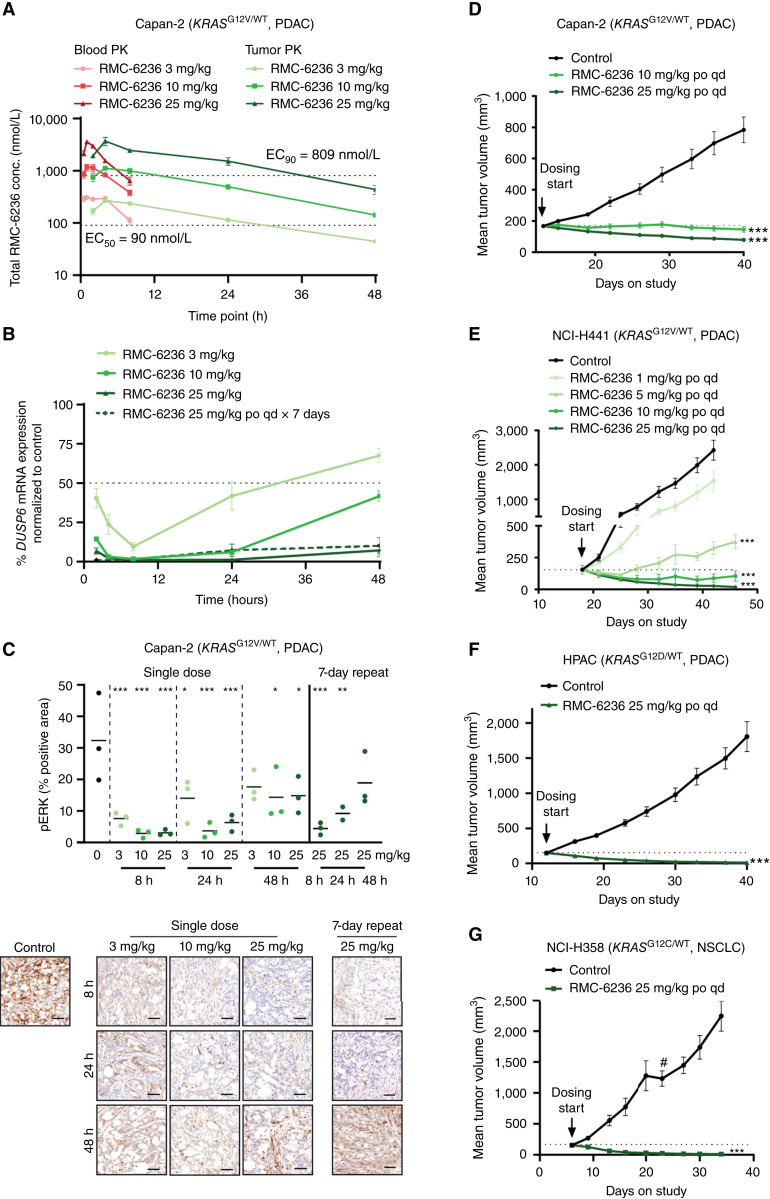
RMC-6236 inhibits RAS signaling and tumor growth and drives tumor regressions *in vivo.***A,** Blood and tumor PK profiles of RMC-6236 in Capan-2 (*KRAS*^G12V/WT^, PDAC) xenograft tumor-bearing BALB/c nude mice. Tumor-bearing mice were treated with a single dose of vehicle or RMC-6236 at 3, 10, or 25 mg/kg. Blood and tumors were harvested at indicated time points (*n* = 3/time point/dose). PK profiles are shown as RMC-6236 concentration in tumors (green lines) and blood (red lines) over time. Shades of green or red represent PK profiles at three tested doses. The dashed lines represent EC_50_ and EC_90_ potency of RMC-6236 in inhibiting *DUSP6* mRNA expression in Capan-2 tumors derived from the PK/PD relationship curve in Fig. 5A. Values are plotted as mean ± SEM. **B,** PD of RMC-6236 in Capan-2 (*KRAS*^G12V/WT^, PDAC) xenograft tumors, shown as the relative change in *DUSP6* mRNA expression. Tumor-bearing mice were treated with a single dose (solid lines) of vehicle, RMC-6236 at 3, 10, or 25 mg/kg, or 7 consecutive daily doses of RMC-6236 at 25 mg/kg (dashed lines). Shades of green represent three tested doses. Solid lines represent a single dose while the dashed line represents repeat dosing. Values are plotted as mean ± SEM. **C,** Histopathology analysis of Capan-2 xenograft tumors treated with a single dose of vehicle control, or RMC-6236 at 3, 10, or 25 mg/kg or 7 consecutive daily doses of RMC-6236 at 25 mg/kg and collected at indicated time points (*n* = 2–3/time point/dose). pERK staining in tumor areas was quantified and compared with vehicle using one-way ANOVA followed by Dunnett multiple comparison test (*, *P* < 0.05; **, *P* < 0.01; ***, P < 0.001). Representative images are shown at 200× magnification from samples closest to the mean of the group. Scale bar, 50 µm. **D–G,** Dose-dependent antitumor activity of RMC-6236 in subcutaneous xenograft models of (**D**) Capan-2 (*KRAS*^G12V/WT^, PDAC; *n* = 8 per group), po qd, per os quaqua (once a day) (**E**) NCI-H441 (*KRAS*^G12V/WT^, NSCLC; *n* = 10 per group), (**F**) HPAC (*KRAS*^G12D/WT^, PDAC; *n* = 10 per group), and (**G**) NCI-H358 (*KRAS*^G12C/WT^, NSCLC; *n* = 8–10 per group). Tumor-bearing mice were treated with vehicle or RMC-6236 at indicated doses for 27–28 days, and mean tumor volumes of each group were plotted over the course of treatment. Vehicle control and RMC-6236 groups were compared by two-way repeated-measures ANOVA on the last measurement day of the vehicle group (***, *P* < 0.001). The dotted line indicates the initial average tumor volume. Error bars, SEM. # indicates 1 animal terminated upon reaching a tumor burden endpoint.

We then assessed the pharmacokinetic (PK) and pharmacodynamic (PD) profile of RMC-6236 as well as the antitumor activity in a series of mutant RAS-driven human tumor xenograft models *in vivo*, beginning with the Capan-2 xenograft model. Dose-dependent blood and tumor exposure were observed following a single dose of RMC-6236 at 3, 10, or 25 mg/kg (Fig. 2A; Supplementary Table S3). RMC-6236 exhibited similar PK profiles across multiple xenograft models and did not accumulate in blood and tumors following repeated doses (Supplementary Table S3). The exposure of RMC-6236 in various xenograft tumors was approximately 3- to 7-fold higher than that in blood, and elimination from tumors was relatively slower. Consistent with the dose-dependent and prolonged exposure in xenograft tumors, oral administration of RMC-6236 led to dose-dependent and durable suppression of RAS pathway signaling as measured by human *DUSP6* (a RAS/MAPK pathway transcriptional target) mRNA expression levels in tumor lysates (Fig. 2B). A single oral dose of 10 or 25 mg/kg RMC-6236 was sufficient to achieve more than 95% inhibition (relative to vehicle control) of tumor *DUSP6* levels at 8 hours after dose; the latter maintained >90% inhibition up to 24 hours after dose, diminishing thereafter in concordance with declining tumor RMC-6236 concentrations (Fig. 2B). Suppression of the RAS signaling pathway was maintained following repeated dosing of RMC-6236, indicating no/minimal pathway adaptation in these tumors.

To confirm our findings, the downstream RAS signaling marker pERK was further evaluated in formalin-fixed, paraffin-embedded sections from vehicle- and RMC-6236–treated Capan-2 and NCI-H441 (*KRAS*^G12V/WT^, NSCLC) xenograft tumors utilizing IHC methods followed by customized marker quantitation of tumor regions on whole-slide images. In Capan-2, dose- and time-dependent inhibition of pERK was observed in tumors treated with 3, 10, or 25 mg/kg of RMC-6236 (Fig. 2C). At 25 mg/kg, RMC-6236 was able to maintain more than 80% inhibition of pERK levels in tumors up to 24 hours post-dose comparable with the inhibition pattern of *DUSP6* levels in tumor lysates. In addition, pERK was also suppressed in a dose- and time-dependent manner in the NCI-H441 model similar to that observed for Capan-2 xenograft tumors (Supplementary Fig. S2A).

Consistent with the deep and durable RAS signaling modulation, daily RMC-6236 treatment resulted in dose-dependent antitumor activity in a series of human tumor xenograft models harboring prevalent *KRAS* mutations, i.e., *KRAS*^G12D^, *KRAS*^G12V^, and *KRAS*^G12C^. RMC-6236 dosed daily at 25 mg/kg was able to drive deep tumor regressions in Capan-2 (Fig. 2D), NCI-H441 (Fig. 2E), HPAC (Fig. 2F), and NCI-H358 (*KRAS*^G12C/WT^, NSCLC, Fig. 2G) following 4 weeks of treatment. At 10 mg/kg, RMC-6236 induced modest regression in sensitive models, leading to 13% and 29% mean tumor regressions in Capan-2 and NCI-H441 models, respectively. In relatively refractory models such as NCI-H2122 (*KRAS*^G12C/G12C^, NSCLC; Supplementary Fig. S2B) and KP-4 (*KRAS*^G12D/WT^, PDAC; Supplementary Fig. S2C), RMC-6236 at 25 mg/kg caused initial tumor regressions, which then relapsed after 2 weeks of treatment, albeit still resulting in tumor control and growth inhibition relative to control groups at a tumor burden endpoint. Interestingly, in both NCI-H2122 and KP-4 models, which showed attenuated response to RMC-6236, a single or repeat daily oral administration of RMC-6236 at 25 mg/kg could still drive potent inhibition of *DUSP6* post last dose (Supplementary Fig. S2D and S2E). Though in the case of KP-4 xenograft tumors, the RAS pathway signaling inhibition was relatively less durable compared with that observed in the more sensitive Capan-2 model (Fig. 2B). KP-4 harbors *MYC* amplification, as well as hyperactivation of upstream RTK signaling via an HGF/MET autocrine loop (28), both of which could contribute to the observed reduction in pathway modulation durability. Repeated oral administration of RMC-6236 was tolerated at all dose levels and in all preclinical models evaluated, as assessed by body weight change (Supplementary Fig. S2F).

### Broad-Spectrum Antitumor Activity of RMC-6236 in Preclinical Models of RAS-Driven Cancers

**Figure 3.**
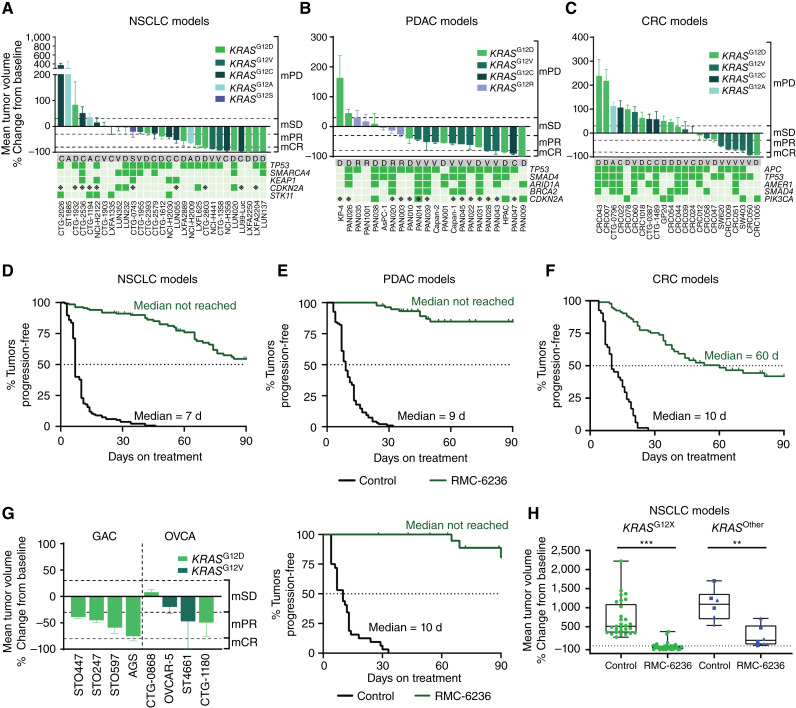
Broad-spectrum antitumor activity of RMC-6236 in preclinical models of RAS-addicted cancers. **A–C,** Tumor response waterfall plots of *KRAS*^G12X^ NSCLC (**A**), PDAC (**B**), and colorectal cancer (**C**) xenograft models upon RMC-6236 daily treatment at 25 mg/kg. 29 NSCLC, 22 PDAC, and 23 colorectal cancer xenograft models were included (*n* = 1–10 per model). Average % mean tumor volume change ± SEM from baseline at response calling date are shown. mRECIST criteria were used to call tumor response as indicated on the right-hand side of each waterfall plot. Oncoplots illustrating gene alterations and expression levels in critical genes linked to the clinicopathologic characteristics of the indicated models are shown below each waterfall. Color coding represents dark green for mutations and light green for the absence of mutations. The ◈ symbol denotes that mRNA expression of corresponding genes not expressed, defined as having a gene-expression value of ≤0.5 CPM. The top row specifically highlights the mutation codon at *KRAS*^G12^. **D**–**F,** Kaplan–Meier analyses of time to tumor doubling on treatment in individual tumor-bearing animals from *KRAS*^G12X^ NSCLC (**D**), PDAC (**E**), and colorectal cancer (**F**) xenograft models upon daily treatment of vehicle control or RMC-6236 at 25 mg/kg for up to 90 days. 29 NSCLC models (*n* = 135 animals each in control and RMC-6236 treatment groups), 22 PDAC models (*n* = 95 animals in control, *n* = 83 in RMC-6236 treatment group), and 23 colorectal cancer models (*n* = 95 animals in control, *n* = 93 in RMC-6236 treatment group) were included. Time to event was determined by the time on treatment until tumor volume doubling from baseline on survival plots by Kaplan–Meier analysis. Log-rank test was used to compare vehicle control with treatment groups, Cox proportional hazards models were used to estimate hazard ratios: *KRAS*^G12X^ NSCLC (HR 0.035, 95% interval 0.020–0.061, *P* < 2 × 10^−16^), *KRAS*^G12X^ PDAC (HR 0.008, 95% interval 0.002–0.026, *P* < 2 × 10^−16^) and *KRAS*^G12X^ colorectal cancer (HR 0.072, 95% interval 0.043–0.120, *P* < 2 × 10^−16^). **G,** Tumor response waterfall plot and Kaplan–Meier analysis of *KRAS*^G12X^ GAC and OVCA xenograft models upon daily treatment of vehicle control or RMC-6236 at 25 mg/kg for up to 90 days. Four models of GAC and 4 models of OVCA tumors were included. Average % mean tumor volume change ± SEM from baseline at the response calling date were plotted. mRECIST criteria were used to call tumor response as indicated on right-hand side of the waterfall plot. Time to event was determined above. **H,** Bar plots of mean tumor volume % change ± SEM from baseline for xenograft models of NSCLC with *KRAS*^G12X^ and *KRAS*^Other^ mutations. Data for both vehicle control and RMC-6236 treatment groups of 35 *KRAS*^MUT^ NSCLC models (29 *KRAS*^G12X^ and 6 *KRAS*^Other^ models) are shown with each model represented by one symbol. The genotype of each model was represented by color and shapes: *KRAS*^G12X^ (green dot), *KRAS*^Other^ (purple; *KRAS*^G13X^, square; *KRAS*^Q61H^, triangle; *KRAS*^K117N^, star). Mean tumor volume % change from baseline of the vehicle control groups and RMC-6236 treatment groups for *KRAS*^G12X^ models are 708.1% and −13.7% respectively; for *KRAS*^Other^ models are 1,070% and 257.5%, respectively. Vehicle control and RMC-6236 treatment groups were compared by paired *t* test, with *P* < 0.001 (***) for *KRAS*^G12X^ models and *P* < 0.01 (**) for *KRAS*^Other^ models. The dotted line represents mean baseline tumor volume.

To evaluate the breadth of antitumor activity of RMC-6236 in *RAS*-mutant human cancers, we conducted a mouse clinical trial (MCT; ref. 29) across a series of xenograft models with *KRAS* hotspot mutations, which represented key RAS-driven cancer indications. Based on the initial assessment above, RMC-6236 was evaluated at a fixed daily dose of 25 mg/kg (tolerable and shown to demonstrate deep and durable RAS pathway suppression; Fig. 2B) in a total of 82 *KRAS*^G12X^ models, including 29 NSCLC, 22 PDAC, 23 colorectal cancer, 4 gastric carcinoma (GAC), and 4 ovarian adenocarcinoma (OVCA) xenograft models (Fig. 3; Supplementary Table S4). Using whole-transcriptome and whole-exome sequencing, we also examined alterations in key genes implicated in NSCLC, PDAC, and colorectal cancer disease etiology (30–32). The gene alteration frequency in each set of models examined was generally concordant with that observed in the Foundation Medicine, Inc. (FMI) database (1) for the corresponding cancer type, indicating that models enrolled in this MCT were representative of the genomic landscape in patients with *KRAS*^G12X^ NSCLC, PDAC, or colorectal cancer, respectively (Supplementary Fig. S3A). Modified Response Evaluation Criteria in Solid Tumors (mRECIST; see Methods for details) were applied to call the initial response in each model following 28 ± 2 days of treatment or when control tumors reached two tumor doublings (whichever was later). Tumor response was called based on the percentage of mean tumor volume change from baseline and categorized into four criteria: progressive disease (mPD), stable disease (mSD), partial response (mPR), and complete response (mCR), yielding an overall response rate [ORR = (mCR + mPR)/total treated] and disease control rate [DCR = (mCR + mPR + mSD)/total treated; ref. 29]. Consistent with RMC-6236 sensitivity and *KRAS* dependency in human cancer cells *in vitro* (Fig. 1D; Supplementary Fig. S1F), RMC-6236 monotherapy drove durable antitumor activity and frequent regressions in *KRAS*^G12X^ xenograft models across all indications tested (Fig. 3A–G; Supplementary Table S4). In particular, the ORR and DCR in *KRAS*^G12X^ NSCLC models (Fig. 3A) were 52% (15/29) and 83% (24/29), and 64% (14/22) and 91% (20/22) in PDAC models (Fig. 3B), respectively. In *KRAS*^G12X^ colorectal cancer models (Fig. 3C), ORR (26%, 6/23) and DCR (52%, 12/23) were lower than those observed in NSCLC and PDAC models; this may reflect the presence of multiple oncogenic drivers and/or EGFR-mediated adaptive feedback to RAS/MAPK pathway signaling inhibition that is particularly resilient in colorectal cancer, as has been observed following BRAF and/or inactive-state selective KRAS^G12C^ inhibition (13, 14). In addition, RMC-6236 drove tumor regressions in 4 of 4 *KRAS*^G12D^ GAC and 2 of 4 *KRAS*^G12X^ OVCA xenograft models (Fig. 3G).

Next, we assessed the durability of the responses depicted above via long-term treatment (up to 90 days) with RMC-6236 (Supplementary Table S5). Kaplan–Meier analyses of this experiment (Fig. 3D–F), wherein tumor progression was defined as individual tumor volume doubling from baseline (29), showed that RMC-6236 treatment resulted in significantly improved progression-free survival (PFS) as compared with vehicle controls in all *KRAS*^G12X^ tumor-bearing animals. Indeed, RMC-6236–treated *KRAS*^G12X^ NSCLC (Fig. 3D) and PDAC tumors (Fig. 3E) did not reach a median time to tumor doubling as the majority of regressions and even cytostatic responses were maintained over 90 days (Supplementary Table S5). Although *KRAS*^G12X^ colorectal cancer tumors exhibited a more heterogeneous response, RMC-6236 also significantly improved PFS in these models, with a 6-fold increase in median time to tumor doubling at 60 days after treatment initiation as compared with 10 days for controls (Fig. 3F). Across all three top *KRAS*^G12X^ indications (i.e., NSCLC, PDAC, and colorectal cancer), 20 models were considered mPR or mCR at response calling date and dosed long-term (more than 60 days). Tumor relapse was observed only in 5 of 20 models with one or more tumors rebounding on treatment. Within this sensitive *KRAS*^G12X^ set of (NSCLC, PDAC, and colorectal cancer) models, it was also interesting to note that *KRAS*^G12V^ models tended to have a higher ORR (71%, 15/21) and DCR (95%, 20/21), and significantly longer durability of response as compared with the other prevalent *KRAS*^G12D^ subset of models (Supplementary Fig. S3B–D). This is interestingly consistent with the biochemical and signaling differences observed following KRAS^G12V^ and KRAS^G12D^ inhibition by RMC-6236 in isogenic systems (Supplementary Fig. S1C), and in antiproliferative sensitivity across a large panel of human cancer cell lines (Supplementary Fig. S1F). Whether these genotype differences in sensitivity will be apparent in the clinical setting remains to be determined.

Based on the biochemical and cellular profiles of RMC-6236 (Fig. 1B and D), we also tested RMC-6236 treatment in xenograft models harboring hotspot oncogenic mutations beyond *KRAS*^G12^ termed *KRAS*^Other^. A panel of 6 *KRAS*^Other^ NSCLC models (3 *KRAS*^G13X^, 2 *KRAS*^Q61H^, and 1 *KRAS*^K117N^) exhibited significant and durable responses to RMC-6236 treatment at 25 mg/kg *in vivo* as compared with controls, albeit the mean reduction in tumor volumes was less marked than that observed in *KRAS*^G12X^ models of NSCLC (Fig. 3H; Supplementary Fig. S3E).

We then examined the status of prevalent genomic/molecular aberrations in each set of models as potential comodifiers of response to RMC-6236 treatment. No significant association was found between RMC-6236 tumor volume response and the presence of functional alterations in genes reflecting major comutation classes and associated with disease etiology in each indication (oncoplots in Fig. 3A–C), albeit we noted a (nonsignificant) trend in the occurrence of oncogenic mutations in *KEAP1* and *SMARCA4* as well as loss of expression of *CDKN2A* in *KRAS*^G12X^ NSCLC models with somewhat reduced responses (Fig. 3A). Given each of these alterations was previously reported to be an independent determinant of a relative reduction in the durability of responses in patients with *KRAS*^G12C^ NSCLC treated with KRAS^G12C^(OFF) inhibitor monotherapy (32, 33), we examined the impact of each on PFS in *KRAS*^G12X^ NSCLC models enrolled in the MCT above (Supplementary Fig. S3F). Interestingly, *KRAS*^G12X^ NSCLC models with loss of expression of *CDKN2A* (*CDKN2A* loss) exhibited a significantly shorter PFS on RMC-6236 treatment as compared with those with intact *CDKN2A* expression. Given that RAS signaling and cell-cycle progression converge at the CDK4/CyclinD (CCND1) axis (34), it is possible that the loss of p16 (the gene product of the gene encoding cyclin dependent kinase 2A (CDKN2A) removes the negative regulation of CDK4/CyclinD and reduces the impact of RAS inhibition on this axis. Comutation of *KEAP1* (*KEAP*^MUT^1) was also associated with reduced durability of response to RMC-6236, whereas *SMARCA4* comutation (*SMARCA4*^MUT^) was not (Supplementary Fig. S3F). Notably, no significant prognostic effect was observed for any of the above-mentioned genomic aberrations on the PFS of *KRAS*^G12X^ NSCLC models in control groups.

Because CypA is indispensable for tri-complex formation, and thus essential for RMC-6236 activity (24), we also evaluated the potential effect of differential CypA expression as a modifier of RMC-6236 response. Typically, *PPIA* is abundantly expressed across cancer types with low endogenous variation in tumor levels (24). Consistent with our *in vitro* findings (Supplementary Fig. S1H), baseline *PPIA* mRNA levels in the *KRAS*^G12X^ xenograft models surveyed above (NSCLC, PDAC, colorectal cancer, GAC, and OVCA) demonstrated minimal variation and no association with RMC-6236 response (Supplementary Fig. S3G and Supplementary Table S4).

### Translating RMC-6236 Activity in NSCLC: Blood–Brain Barrier Dynamics, Overcoming Resistance to Mutant-Selective Inhibitors, and Combination with Checkpoint Inhibition

**Figure 4.**
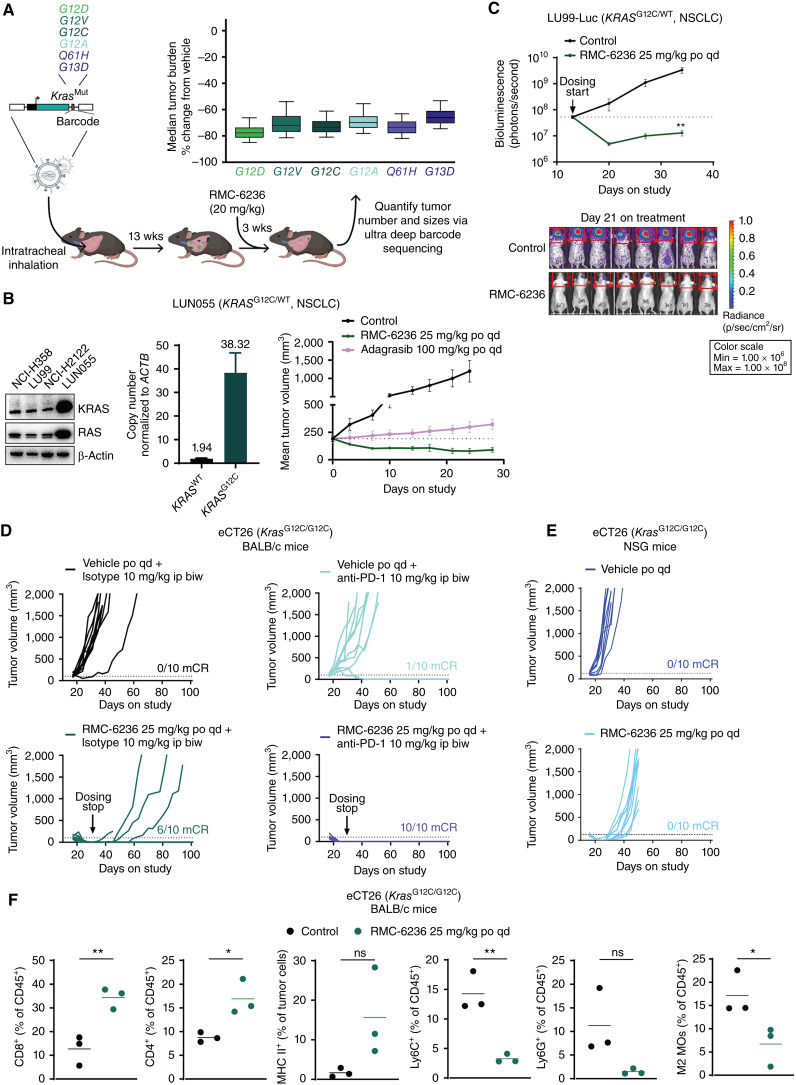
Translating RMC-6236 activity in NSCLC. **A,** Efficacy of RMC-6236 on *Kras*^G12C^, *Kras*^G12D^, *Kras*^G12V^, *Kras*^G12A^, *Kras*^G13D^, or *Kras*^Q61H^-driven autochthonous lung tumors in immunocompetent mice. A pool of lentiviral cDNA vectors encoding each oncogenic *Kras* variant was delivered intratracheally to the lungs of each mouse, and 13 weeks after tumor growth, mice were treated with RMC-6236 at 20 mg/kg po qd for 3 weeks prior to analysis. 95% confidence intervals are shown. **B,** Efficacy of RMC-6236 and adagrasib in the LUN055 NSCLC PDX model with *KRAS*^G12C^ allele copy-number gain. Immunoblot Western analyses (left) of RAS and KRAS protein levels in NCI-H358 (*KRAS*^G12C/WT^, NSCLC), LU99 (*KRAS*^G12C/WT^, NSCLC), NCI-H2122 (*KRAS*^G12C/G12C^, NSCLC), and LUN055 (*KRAS*^G12C/WT^, NSCLC) xenograft tumors. Relative copy-number (middle) of *KRAS*^WT^ or *KRAS*^G12C^ in LUN055 xenograft tumors (*n* = 2) were determined by ddPCR and normalized to *ACTB*. LUN055 xenograft tumor-bearing mice were treated with vehicle or RMC-6236 at 25 mg/kg po qd or adagrasib at 100 mg/kg po qd for 24 to 28 days (*n* = 3 per group, right). Mean tumor volumes of each group were plotted over the course of treatment. Dotted line indicates the initial average tumor volume. Error bars, SEM. **C,** Efficacy of RMC-6236 in the intracranially implanted LU99-Luc (*KRAS*^G12C/WT^, NSCLC) xenograft model (*n* = 8 per group). RMC-6236 was dosed at 25 mg/kg daily for 21 days. Images of bioluminescence in individual mice were shown. Bioluminescence of ROI in vehicle control and RMC-6236 groups were compared by two-way repeated-measures ANOVA at day 21 (**, *P* < 0.01). Results were shown as mean ± SEM. **D,** Antitumor activity of RMC-6236 and the combination with anti–PD-1 (clone RMP1-14, rat IgG2a) following repeated administration in BALB/c mice bearing the murine colon carcinoma eCT26 (*Kras*^G12C/G12C^) shown as individual tumor growth curves (*n* = 10 per group). Graphs indicate the number of complete regressions per injected mice. RMC-6236 and anti–PD-1 treatment started on day 17 after implantation. RMC-6236 treatment was stopped at day 31 after implantation and anti–PD-1 at day 35 after implantation. **E,** Antitumor activity of RMC-6236 following repeated administration in NSG mice bearing the murine colon carcinoma eCT26 (*Kras*^G12C/G12C^) shown as individual tumor growth curves (*n* = 10 per group). Graphs indicate the number of complete regressions per injected mice. RMC-6236 treatment started on day 16 after implantation. **F,** Immune cell composition (CD8^+^ and CD4^+^ T cells, Ly6C^+^ and Ly6G^+^ myeloid-derived suppressor cells and M2 macrophages) in murine colon carcinoma eCT26 syngeneic tumors (*Kras*^G12C/G12C^) represented as percentage of CD45^+^ cells and expression of cell-surface markers on viable, CD45^−^ large cells (assessed as tumor cells) 24 hours post 4 days of treatment with vehicle or RMC-6236 at 25 mg/kg po qd *n* = 3 biological replicates/group represented as mean; *, *P* < 0.05; **, *P* < 0.01; ns, nonsignificant by two-sided Student *t* test.

Given the potential for a RAS(ON) multi-selective inhibitor in mutant *KRAS*-driven NSCLC, we investigated key translational elements of RMC-6236 activity in relevant preclinical models. First, we directly compared the therapeutic impact of RMC-6236 across diverse *Kras* mutant genotypes in a quantitative and internally controlled autochthonous genetically engineered mouse model system (35, 36). We initiated autochthonous *Kras* G12C-, G12D-, G12V-, G12A-, G13D-, and Q61H-driven lung tumors in parallel, each with unique barcodes within individual immunocompetent mice (Fig. 4A). This enabled us to assess RMC-6236 activity across a spectrum of individual *Kras* variant NSCLC tumors within the same animal as well as across separate cohorts of RMC-6236 and vehicle-treated mice. Ultra-deep sequencing of thousands of tumor barcodes unique to each individual tumor revealed that daily treatment with RMC-6236 drove significant and consistent reductions in tumor burden (median number of neoplastic tumor cells in RMC-6236–treated mice relative to vehicle-treated mice) across all oncogenic *Kras* variants tested (Fig. 4A).

Interestingly, clinical and preclinical evidence suggests that this spectrum of oncogenic *KRAS* point mutations (capable of driving tumorigenesis) is often exploited to reactivate RAS signaling and drive resistance following treatment with KRAS mutant-selective therapies, e.g., in patients with *KRAS*^G12C^ NSCLC tumors treated with sotorasib, adagrasib, or divarasib (22, 23, 37). In each of these cases, preexisting and acquired *KRAS* codon 12, 13, and 61 mutations, and second-site alterations in *cis* within the *KRAS*^G12C^ allele itself, have been commonly observed. To address the latter, we examined RMC-6236 activity in autochthonous lung tumors driven by *Kras*^G12C^ harboring secondary H95D or Y96C alterations and found that RMC-6236 treatment again drove significant reductions in tumor burden relative to vehicle control-treated mice (Supplementary Fig. S4A).

In addition to second-site alterations, copy-number gain of the *KRAS*^G12C^ allele itself is considered a putative on-target resistance mechanism to KRAS^G12C^(OFF) inhibitors (21). Because RMC-6236 targets the active, GTP-bound form of RAS proteins, we hypothesized that RMC-6236 may be more active in cancer cells with *KRAS*-mutant allele copy-number gain than those inhibitors targeting the GDP-bound form of RAS proteins. As shown previously in this paper (Supplementary Fig. S1J), *KRAS* gene copy-number gain at baseline did not affect RMC-6236 median sensitivity *in vitro*. We also evaluated this hypothesis *in vivo* as shown in Fig. 4B: LUN055 (*KRAS*^G12C/WT^, NSCLC) is a patient-derived xenograft model of human NSCLC with *KRAS*^G12C^ allele copy-number gain and KRAS protein overexpression. Daily RMC-6236 treatment showed a clear improvement in the depth of response as compared with adagrasib treatment in this model, leading to tumor regressions, whereas the latter achieved tumor growth inhibition. However, it is worth noting that any resistance mechanism that leads to increased mutant RAS(ON), through either gene copy-number gains, mRNA or protein overexpression, or upstream RTK activation, may attenuate RAS inhibitor potency, including that of RMC-6236.

Brain metastases frequently occur in patients with *KRAS*^MUT^ NSCLC and impact the prognosis (38). Therefore, we examined the potential of RMC-6236 to penetrate the brain and into intracranial tumors in rodents. In naïve (nontumor-bearing) BALB/c mice, RMC-6236 was quantifiable in brain tissue, and concentrations increased in a dose-dependent manner (Supplementary Table S3). In intracranial tumor models that mimic brain metastases in mice (39), RMC-6236 tumor concentrations were comparable to levels in adjacent normal brain at matching time points, which in turn were similar to levels in naïve animal brains following a single dose of RMC-6236. These results indicated that RMC-6236 could penetrate the central nervous system and that the surgical implantation procedures that we used to generate the intracranial models tested herein resulted in minimal damage to the blood–brain barrier. Daily oral administration of RMC-6236 at 25 mg/kg was tolerated and led to tumor regressions as assessed via bioluminescent signals in two intracranially implanted (luciferase-expressing) xenograft models of human *KRAS*^G12C^ mutant NSCLC: LU99 and NCI-H1373 (Fig. 4C; Supplementary Fig. S4B and S4C).

Lastly, we examined the impact of RMC-6236 treatment alone and in combination with immune-checkpoint inhibition in a representative model of immunogenic *KRAS*^G12C^-mutant cancers, i.e., an engineered murine syngeneic tumor model harboring a homozygous *Kras*^G12C^ mutation (eCT26 *Kras*^G12C/G12C^) in immune-competent BALB/c mice. RMC-6236 monotherapy at 25 mg/kg was sufficient to drive complete tumor regressions in all animals over a period of 14 days, with 60% (6 of 10) of animals maintaining these mCRs following treatment withdrawal (Fig. 4D). Consistent with reports that abrogation of the immune evasive effects of oncogenic *Kras* can sensitize tumors to immune-checkpoint blockade (40), the combination of RMC-6236 with antiprogrammed death protein-1 (anti–PD-1, clone RMP1-14, rat IgG2a) resulted in durable complete tumor regressions in all animals (Fig. 4D; Supplementary Fig. S4D). To further assess the role of antitumor immunity during the response to RMC-6236, eCT26 *Kras*^G12C/G12C^ tumors were treated for 34 days in immune-deficient NOD-SCID/IL2Rg^null^ (NSG) mice. Although in immunocompetent mice all animals achieved mCRs after 14 days of treatment with RMC-6236, in immunodeficient mice all tumors relapsed on treatment, suggesting that the immune compartment is essential for the generation of long-term mCRs (Fig. 4E). Next, a rechallenge experiment with the same eCT26 *Kras*^G12C/G12C^ tumor cells was performed in all the tumor-free immune-competent BALB/c mice remaining at day 161 (post-tumor implantation) to assess the development of immunologic memory. All mice with mCRs withstood the rechallenge and remained tumor-free, indicating the presence of immunologic memory (Supplementary Fig. S4E). Analysis of tumor immune cell composition after 4 days of RMC-6236 administration showed a significant increase of CD4^+^ and CD8^+^ T cells in the tumor microenvironment (TME), relative to tumors from vehicle controls (Fig. 4F). Monocytic and granulocytic myeloid-derived suppressor cells, as well as M2 macrophages, were decreased in response to RMC-6236 treatment. In addition, RMC-6236 drove an increase of MHC class II–positive tumor cells. A similar modification of the TME in favor of antitumor immunity was observed in eCT26 tumors harboring the original homozygous *Kras^G12D^* mutation (eCT26 *Kras*^G12D/G12D^). RMC-6236 plus anti–PD-1 also showed combination benefit in this model, inducing durable responses and immunologic memory (Supplementary Fig. S4F–I). Taken together, these results indicate that RMC-6236 treatment can drive durable antitumor immunity in models of both *Kras*^G12C^ and *Kras*^G12D^ mutant cancers *in vivo*, and these effects are enhanced in combination with immune-checkpoint inhibition, a key standard-of-care treatment for patients with NSCLC (40).

### Effects of RMC-6236–Mediated Pharmacologic Modulation of RAS Pathway Signaling in Tumor-Bearing Mice

**Figure 5.**
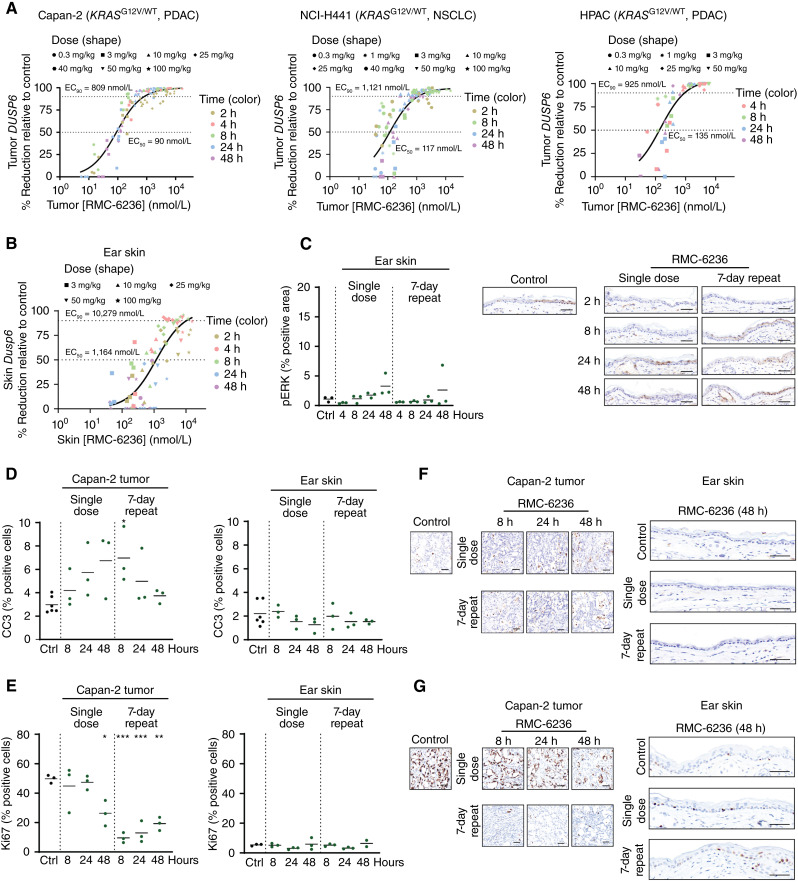
Effects of RMC-6236 mediated pharmacologic modulation of RAS pathway signaling in tumor-bearing mice. **A,** PK/PD relationship between RMC-6236 concentration and inhibition of *DUSP6* expression in Capan-2 (EC_50_ = 90 nmol/L and EC_90_ = 809 nmol/L), NCI-H441 (EC_50_ = 117 nmol/L and EC_90_ = 1,121 nmol/L), and HPAC (EC_50_ = 135 nmol/L and EC_90_ = 925 nmol/L) xenograft tumors. Subcutaneous xenograft tumors were treated with vehicle or RMC-6236 ranging from 0.3 to 100 mg/kg (Capan-2 and H441) or to 50 mg/kg (HPAC). **B,** PK/PD relationship between RMC-6236 concentration and inhibition of *Dusp6* expression in ear skin (EC_50_ = 1,164 nmol/L and EC_90_ = 10,279 nmol/L) isolated from tumor-bearing BALB/c nude mice treated with vehicle or RMC-6236 ranging from 3 mg/kg to 100 mg/kg. **A** and **B,** Tumors and ear skin from tumor-bearing BALB/c nude mice were harvested at indicated time points (*n* = 3/timepoint/dose). A 3-parameter sigmoidal exposure–response model was fitted to the data to derive EC_50_ and EC_90_ values. Time points are represented by colors and doses are represented by symbol shapes. **C–G,** Histopathology of tumors and ear skin from the Capan-2 xenograft model collected at indicated time points post a single dose of vehicle control, RMC-6236 at 25 mg/kg or 7 consecutive daily doses of RMC-6236 at 25 mg/kg (*n* = 3–6/time point/dose). Staining of indicated markers in the tumor area or ear skin was quantified and compared with vehicle using one-way ANOVA followed by the Dunnett multiple comparison test (*, *P* < 0.05; **, *P* < 0.01; ***, *P* < 0.001). Representative images are shown at 200× magnification from samples closest to the mean of the respective groups. Scale bar, 50 µm.

To characterize the mechanistic basis of the broad antitumor activity of RMC-6236 in *KRAS*^MUT^ (especially *KRAS*^G12X^) models, we conducted detailed and quantitative analyses of the pharmacologic profile of RMC-6236 in xenograft tumors and representative normal tissues from tumor-bearing animals. First, the relationship between RMC-6236 concentration in tumors (tumor PK) and RAS pathway inhibition, as measured by human *DUSP6* levels (tumor PD), in xenograft tumors was investigated by compiling a data set from multiple PK/PD studies on each model (Supplementary Table S6). In the Capan-2 model, RAS pathway inhibition was tightly associated with RMC-6236 tumor concentrations (Fig. 5A), and the calculated EC_50_ value of 90 nmol/L from this PK/PD relationship was close to that observed in the biochemical KRAS^G12V^-RAF RBD disruption assay (Fig. 1B; Supplementary Fig. S1B). A single dose of 25 mg/kg resulted in tumor RMC-6236 exposure above the EC_90_ (809 nmol/L) for over 24 hours (Fig. 2A), while repeated daily dosing at 25 mg/kg drove tumor regressions in all animals. At a dose of 10 mg/kg, which drove regressions in about half of the evaluated tumors, RMC-6236 tumor concentration crossed EC_50_ but only reached EC_90_ transiently in a 24-hour period. These data suggest that the local tumor concentration of RMC-6236 is a critical determinant of pharmacodynamic pathway modulation and that maintaining RMC-6236 tumor concentrations above EC_90_ is necessary to drive maximal suppression of tumor growth consistently in this model. A close and comparable PK/PD relationship was also apparent in two additional xenograft models, NCI-H441 and HPAC (Fig. 5A), demonstrating that the pharmacologic potency of RMC-6236 for inhibition of RAS pathway activity can be determined from the exposure–response relationship described herein. These data also substantiate our rationale for daily dosing of RMC-6236 at a dose of 25 mg/kg to achieve target coverage over an inhibition threshold (EC_90_) throughout the 24-hour dosing interval and consistently drive deep tumor regressions in RAS pathway–dependent models. In agreement with these findings, the same dose of 25 mg/kg RMC-6236 covered EC_50_ (454 nmol/L) for about 24 hours but did not cross EC_90_ (2893 nmol/L) in a less sensitive model, i.e., KP-4 (Supplementary Fig. S5A), wherein repeated RMC-6236 dosing drove tumor growth inhibition but could not sustain tumor regressions (Supplementary Fig. S2C).

The broad anticancer activity of RMC-6236 in a wide range of preclinical models of RAS-addicted solid tumors at dose levels that were well tolerated raises the question of how normal tissues respond to the inhibition of RAS-GTP signaling. To explore this, we examined the concentration–response relationship of RMC-6236 for murine *Dusp6* mRNA inhibition in two self-renewing tissues with proliferative compartments, i.e., skin and colon (41, 42). The potency of RMC-6236 for RAS pathway modulation in both normal tissues was appreciably lower than that in tumor cells of Capan-2, NCI-H441, and HPAC models, exhibiting a ≥10-fold shift in EC_50_ and EC_90_, respectively, in both skin and colon tissues from tumor-bearing animals (Fig. 5B; Supplementary Fig. S5B). In fact, only transient pERK suppression was observed in the skin from Capan-2 tumor-bearing animals in contrast to the deep and durable pathway modulation observed in tumors (compare Fig. 2B, C with Fig. 5C). Notably, RMC-6236 exposure in both skin and colon after a single dose at 25 mg/kg remained well below EC_90_ for these tissues at all times and dropped below the EC_50_ between 8 and 24 hours after dose, consistent with the transient RAS signaling modulation observed (Supplementary Table S6).

We then examined the downstream consequences of RAS inhibition in both RAS-addicted tumors and in normal tissues including ear skin (Fig. 5D–G) and colon (Supplementary Fig. S5C). We hypothesized that, in contrast to mutant RAS-addicted tumor cells, normal cells have reduced levels and/or dependence on RAS-GTP and use homeostatic mechanisms to restore equilibrium following perturbation of RAS signaling (43, 44). In Capan-2 xenograft tumors, we observed a notable increase in CC3-positive cells and a significant decrease in actively proliferating cells relative to vehicle controls after single and 7-day repeat dosing of RMC-6236 at 25 mg/kg (Fig. 5D–G). In contrast, few apoptotic cells were observed in the matched skin and colon tissues from these tumor-bearing mice, and no apparent effect on the proliferation of ear skin cells was detected (Fig. 5D–G; Supplementary Fig. S5C). Together, these results highlight a marked difference in the potency and kinetics of RMC-6236–mediated PD pathway modulation between RAS mutant oncogene-addicted tumors and normal cells. The differences in apoptosis induction and proliferative indices reflect key differences in how normal tissues respond and adapt to RAS inhibition with RMC-6236 compared with tumors driven by mutant *KRAS*, providing a rational basis for the tumor selectivity of RAS inhibition.

### PK/PD/Efficacy Modeling to Predict a Clinically Active Dose Range

**Figure 6.**
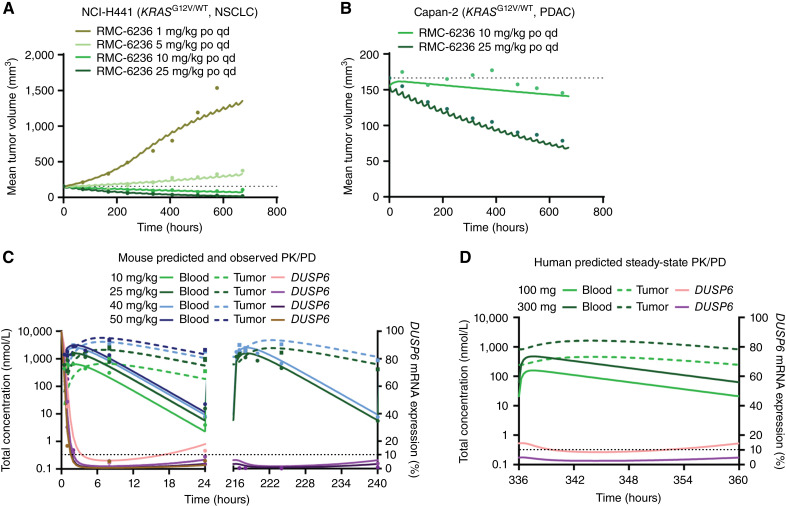
PK/PD/Efficacy modeling to predict clinically active dose range. **A,** Comparison of observed and predicted tumor growth data at multiple dose levels of RMC-6236 in the NCI-H441 xenograft tumor model. Tumor growth was predicted using the Simeoni tumor growth model. The dotted line indicates the initial average tumor volume. **B,** Comparison of observed and predicted tumor growth data at multiple dose levels of RMC-6236 in the Capan-2 xenograft tumor model. Tumor growth was predicted using the Simeoni tumor growth model. The dotted line indicates the initial average tumor volume. **C,** Comparison of observed vs. simulated PK and PD data at multiple dose levels of RMC-6236 in mice bearing NCI-H441 xenograft tumors. Single-dose data from all dose levels are presented from 0 to 24 hours, whereas repeat-dose data from 25 and 40 mg/kg repeat daily dosing is presented from 216 to 240 hours. Simulated blood and tumor PK data are indicated by the solid and dashed green and blue lines, respectively. Simulated PD data are indicated by the solid purple lines. Observed data are indicated by dots (blood PK and PD) or squares (tumor PK). The dotted line indicates the 10% expression level of *DUSP6* mRNA as normalized to the vehicle control group. **D,** Predicted profiles of human whole blood and tumor PK as well as tumor PD at clinical dose levels at steady state. Blood PK and tumor PK are indicated by the solid and dashed green lines whereas tumor PD is indicated by the solid purple lines. Repeat dose data are presented from 336 to 360 hours after two weeks of simulated daily dosing. The dotted line indicates a 10% expression level of *DUSP6* mRNA as normalized to the vehicle control group.

We next used PK/PD/efficacy modeling to relate RMC-6236 blood and tumor concentrations, tumor PD, and antitumor activity to establish target exposure and predict an active dose range in humans (Supplementary Methods). As shown in the studies described herein, RMC-6236 treatment resulted in modest tumor regressions (10%–29% mean regression after 4-week treatment) at 10 mg/kg daily dosing, while deep and durable tumor regression (50%–80% mean regression after 4-week treatment) was achieved at 25 mg/kg daily dosing in relatively sensitive models such as Capan-2 and NCI-H441 (Fig. 2D and E). We first developed a preclinical PK/efficacy model to explore the relationship between blood exposure of RMC-6236 and antitumor activity. The Simeoni tumor growth model (45) adequately described the tumor volume data in both NCI-H441 and Capan-2 xenograft models (Fig. 6A and B) and identified 158 nmol/L as the average blood threshold concentration (C_T_) required for tumor stasis across both models. Daily dosing of approximately 9 mg/kg in mice is projected to maintain an average blood concentration (C_avg_) above this threshold. Correcting for species differences in blood–plasma partitioning and plasma protein binding resulted in a human equivalent C_T_ of approximately 80 nmol/L. Based on simulated mean human PK profiles, we anticipated that a daily dose of approximately 100 mg could maintain C_avg_ above the estimated C_T_ and likely result in disease control (tumor stasis to modest regressions) in patients with mutant RAS-driven tumors. Similarly, a daily dose of approximately 300 mg was projected to achieve the equivalent mean blood exposure (C_avg_) observed upon 25 mg/kg daily dosing in xenograft tumor-bearing mice.

Next, a translational PK/PD model relating blood PK to tumor PK and PD (46) was developed to investigate the level of PD modulation associated with tumor regression in NCI-H441 xenografts and to estimate the PD modulation expected at the projected clinically active dose levels in humans. The animal model was able to appropriately capture mouse blood and tumor PK as well as tumor PD from multiple experiments. In the xenograft PK/PD model, a daily dose of 10 mg/kg achieved ≥90% maximal *DUSP6* suppression albeit this suppression was transient (Fig. 6C). Additionally, a daily dose of 25 mg/kg achieved sustained *DUSP6* suppression of 90%–95% over the entire dosing interval at steady state.

Subsequently, we scaled the model to humans and predicted mean *DUSP6* suppression in tumors for two clinical dose levels, as shown in Fig. 6D. A daily dose of approximately 100 mg was estimated to transiently achieve ≥90% maximal *DUSP6* suppression in human tumors at steady state, comparable to the PD modulation profiles observed at 10 mg/kg in xenograft models. Furthermore, a daily dose of approximately 300 mg was estimated to maximize and maintain ≥90% mean *DUSP6* suppression across the dosing interval in tumors at steady state, closely matching the PD response at 25 mg/kg in xenograft models. In summary, our models predicted that a daily dose range of 100 to 300 mg would be clinically active in patients with mutant RAS-driven tumors. Furthermore, the higher dose of 300 mg daily would be expected to drive deep and sustained pathway inhibition and to maximize antitumor activity.

### The Preclinical Antitumor Activity of RMC-6236 Translates into Responses in Patients with Advanced *KRAS*^G12^ NSCLC and PDAC

**Figure 7.**
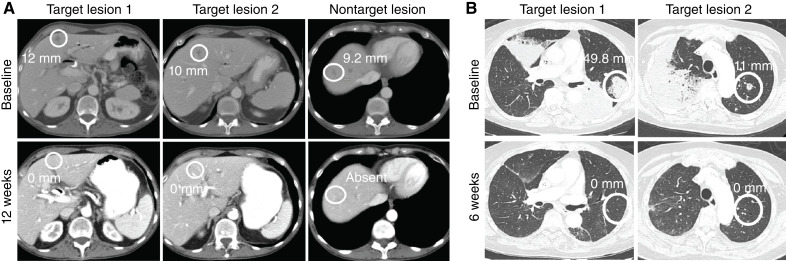
Activity of RMC-6236 in pancreatic and lung cancer patients. **A,** Pretreatment and 12-week (post cycle 4) scans of a heavily pretreated patient with a *KRAS*^G12D^ mutation-positive PDAC indicating a complete response of both target and nontarget lesions. Patient continued on study treatment in cycle 6. Axial views of computed tomography (CT) abdomen images prior to RMC-6236 treatment (top) and after four cycles of RMC-6236 treatment (bottom). **B,** Pretreatment and 6-week (post cycle 2) scans of a patient with a *KRAS*^G12V^ mutation-positive NSCLC indicating a complete response of target lesions (no nontarget lesions present at baseline), atelectatic changes in the right lung are also largely resolved by 6 weeks. Complete response was confirmed at cycle 4, and the patient continued on study treatment with a complete response in cycle 10. Axial views of computed tomography (CT) chest images prior to RMC-6236 treatment (top) and after two cycles of RMC-6236 treatment (bottom).

A first-in-human clinical trial of RMC-6236 (NCT05379985) opened enrollment in May 2022 to assess the safety, tolerability, and initial efficacy of RMC-6236 monotherapy in patients with previously treated, advanced solid tumors. Two cases of patients treated in this trial are provided here to show selected examples of the preliminary clinical activity of RMC-6236 as monotherapy (Fig. 7).

#### Case 1

A 77-year-old woman with metastatic *KRAS*^G12D^ mutated pancreatic ductal adenocarcinoma with liver and peritoneal metastases. Local laboratory testing of biopsied disease also revealed a *SMAD4* deletion. The patient was previously treated with FOLFIRINOX chemotherapy, after which she progressed with growing liver metastases. She was enrolled in the RMC-6236-001 study at 300 mg daily oral dosing in 21-day cycles. The only adverse event she experienced was a single episode of grade 1 vomiting. Disease assessment after the first two cycles showed a partial response with a 68% reduction in her target liver lesions, and disease evaluation after four cycles showed a complete response per RECIST 1.1 with no evidence of disease remaining on CT scans. The complete response was subsequently confirmed on follow-up scans after a total of 6 cycles of RMC-6236 therapy (Fig. 7A). This patient remained on treatment after 5 months without evidence of disease.

#### Case 2

An 83-year-old woman with metastatic *KRAS*^G12V^ mutated NSCLC refractory to multiple lines of therapy including ipilimumab/nivolumab, carboplatin/pemetrexed, and paclitaxel. Local laboratory testing showed comutation in *NFE2L2* and *CDKN2A* deletion. The patient was enrolled on the RMC-6236-001 trial at the 300 mg oral dose, given daily in 21-day cycles; the patient was dose-reduced to 200 mg daily in cycle 4 secondary to adverse events of grade 2 fatigue and grade 1 diarrhea. Other adverse events possibly related to RMC-6236 included grade 2 rectal and vaginal irritation that resolved while on treatment with supportive care measures, grade 1 nausea, grade 1 diarrhea, and grade 1 weight loss. Disease evaluation after two cycles of therapy demonstrated a RECIST 1.1 complete response with a 100% decrease in both target lesions and no nontarget lesions at baseline. The complete response was confirmed on subsequent scans. This patient remained on treatment after 8 months without evidence of disease by imaging (Fig. 7B).

## DISCUSSION

Direct pharmacologic targeting of canonical RAS proteins has been an aspirational goal since the discovery of the RAS oncogenes over 40 years ago (47, 48). Activating oncogenic point mutations in RAS GTPases result in impaired GTP hydrolysis activity and an accumulation of active GTP-bound RAS proteins, which in turn causes increased oncogenic flux and uncontrolled cell proliferation (49). Thus, inhibition of the active GTP-bound state of RAS (RAS(ON)) is likely a preferred therapeutic strategy in RAS-dependent tumors. The advent of inactive-state KRAS^G12C^ inhibitors has provided an elegant clinical proof of concept for the benefits of targeting mutant KRAS, resulting in a surfeit of covalent KRAS^G12C^ inhibitors entering clinical evaluation (9, 10, 37). These are closely being followed by early clinical testing of inhibitors of the inactive state of KRAS^G12D^ as well as “pan-KRAS” inhibitors that begin to address the unserved patient populations harboring more common *KRAS*^G12^ mutations (18, 20). However, all these mutant-selective and “pan-KRAS” inhibitors target the GDP-bound inactive state of the RAS-GTPase(s) in question and are susceptible to perturbations that drive the cellular equilibrium toward the GTP-bound active state of RAS proteins in tumor cells, e.g., increased upstream RTK signaling. Consistent with this notion, reactivation of RAS pathway signaling via diverse mechanisms comprises a large proportion of resistance mechanisms to KRAS^G12C^ inhibitors (thus far) and reinforces the concept that active state RAS(ON) inhibition has the potential to be a superior therapeutic strategy (44, 49).

Here, we describe RMC-6236, a potent, oral RAS(ON) multi-selective tri-complex inhibitor designed to treat cancers driven by a variety of RAS mutations, and with the potential to overcome many of the frequent resistance mechanisms reported following inactive-state RAS-GDP inhibition. Inhibition of all canonical RAS isoforms at once has broad therapeutic potential and applicability but raises the key question of whether this will be tolerated in mammals given the critical role of RAS proteins in embryonic development and normal tissue homeostasis. The discovery of RMC-6236 heralds a long-awaited evaluation of these questions in nonclinical species and in humans. We hypothesized that RAS-GTP inhibition via RMC-6236 would be effective in RAS-dependent (or RAS-addicted) tumor cells but would spare normal cells and tissues. This hypothesis was based on the relatively low levels of active RAS-GTP in normal tissues altogether (50) and the homeostatic mechanisms that exist in proliferative normal cells (51, 52) that may restore equilibrium following the therapeutic pressure exerted by RMC-6236.

Exposure to RMC-6236 suppressed RAS signaling and cell growth and induced apoptosis in multiple human RAS-addicted cancer cell lines *in vitro*. RMC-6236 induced dose-dependent, deep, and durable suppression of RAS pathway activation in preclinical xenograft models *in vivo*, and consequently induced profound and durable tumor regressions in multiple RAS^MUT^ cell line–derived xenograft (CDX) and patient-derived xenograft (PDX) models of (but not restricted to) NSCLC, colorectal cancer, and PDAC. Antitumor activity was particularly notable in tumor models dependent on *KRAS* position 12 mutations (*KRAS*^G12X^), albeit clearly extended to models with other *KRAS* hotspot mutations. Moreover, majority of observed responses at 25 mg/kg dosed daily, whether tumor regressions or tumor growth inhibition, were durable out to 90 days of treatment, predicting that RMC-6236 treatment would achieve durable tumor control in patients with RAS-driven cancers at a tolerated dose. We also found that of the prevalent genomic aberrations reported to modify responses to targeted RAS pathway inhibition with approved KRAS^G12C^ inhibitors (32, 33), mutations in *SMARCA4* were not associated with impaired RMC-6236 effects on durability of response in *KRAS*^G12X^ NSCLC xenograft models, while *KEAP1* mutation or loss of *CDKN2A* expression were associated with a less durable response, opening the door for rational combinations to enhance durability.

Additionally, RMC-6236 promoted antitumor immunity *in vivo* and was additive with anti–PD-1-mediated immune-checkpoint inhibition, driving durable complete responses and immunologic memory in a *Kras*-mutant syngeneic mouse model. This is particularly relevant in the translation of RMC-6236 for the treatment of patients with NSCLC, wherein a combinatorial approach is most likely to benefit the majority. Also, of potential relevance for patients with NSCLC, RMC-6236 was found to cross the intact blood–brain barrier and was active in intracranially implanted tumors in animals. Taken together, these preclinical results support the initial inclusion of patients with solid tumors harboring *KRAS*^G12X^ mutations in a phase I clinical trial with RMC-6236 and the potential expansion to a larger population of RAS-addicted tumor types (53). Furthermore, we demonstrated that RAS(ON) multi-selective inhibition has the potential to overcome RAS signaling reactivation when it arises as a resistance mechanism to mutant-selective RAS inhibition (https://doi.org/10.1038/s41586-024-07205-6).

Having demonstrated that selective noncovalent RAS-GTP inhibition via the tri-complex modality was feasible, tolerable, and effective in preclinical systems, as exemplified by RMC-7977 (https://doi.org/10.1038/s41586-024-07205-6) and RMC-6236 (here), we examined how best to translate these findings into a useful human dosing paradigm. We deployed established PK/efficacy and PK/PD modeling approaches to inform desirable human dose(s) to achieve optimal tumor control and objective responses and to link these to the depth and duration of RAS pathway suppression. As expected for a driver oncogenic pathway, we predicted that near-complete and durable inhibition of RAS signaling would be desirable to maximize objective responses to RMC-6236 monotherapy and to benefit the broadest scope of patients with RAS-dependent cancers. In the ongoing phase I study, we observed initial clinical activity with RMC-6236 starting at 80 mg dosed daily in patients, consistent with our <100 mg dose predictions (53). The two case studies presented here show selected examples of the activity of RMC-6236 dosed at 300 mg given daily (in 21-day cycles), equivalent to the optimal preclinical dose of 25 mg/kg that achieved durable target coverage and ≥90% pathway suppression for a 24-hour period per our model predictions. Each of these patients demonstrated an objective response after two cycles of therapy and remained on treatment at the time of manuscript submission.

The discovery and ongoing development of the investigational agent RMC-6236 realizes the aspiration of directly and concurrently targeting the multiple active RAS isoforms that drive and sustain oncogenesis in a significant fraction of human cancers. The distinctive noncovalent RAS(ON) multi-selective tri-complex inhibitor series, as described here and in Holderfield and colleagues (https://doi.org/10.1038/s41586-024-07205-6), enables the evaluation, including in human subjects, of a long-standing collection of biological principles in the fields of RAS oncology and drug development. The initial results described herein support the assessment of RMC-6236 monotherapy in a variety of RAS-addicted tumors (NCT05379985), as well as the evaluation of a combination with immune-checkpoint modulation in NSCLC (NCT06162221). Finally, we posit that broad-spectrum inhibition of mutant and wild-type RAS in active, GTP-bound states has the potential to serve as the foundation of additional therapeutic combinations aimed at RAS signaling and parallel oncogenic pathways designed for increasingly enduring patient benefit.

## METHODS

### X-Ray Crystallography Methods

#### Protein Production.

His6-TEV-KRAS4B G12D, C51S, C80L, C118S [residues 1–169], and His6-TEV-CYPA [full-length] were expressed from a pET28 vector in BL21(DE3) *E. coli* and purified as described previously (24).

#### Tri-Complex Crystallization.

Purified human CypA (HUGO symbol PPIA) and KRAS G12D, C51S, C80L, C118S bound to GMPPNP were combined in a 2:1 CypA:RAS molar ratio in a buffer solution consisting of 12.5 mmol/L HEPES-NaOH pH 7.3, 75 mmol/L NaCl, and 5 mmol/L MgCl_2_. RMC-6236 was added from a 10 mmol/L DMSO stock to give solutions of 100 µmol/L KRAS, 200 µmol/L CypA, and 300 µmol/L RMC-6236 in 1 mL total volume. These mixtures were incubated for 5 minutes on ice and the tri-complexes were purified via gel filtration using a Superdex 75 10/300 GL column preequilibrated with a buffer consisting of 12.5 mmol/L HEPES-NaOH pH 7.3, 75 mmol/L NaCl, and 5 mmol/L MgCl_2_. Fractions containing the tri-complex were pooled and concentrated to 15 mg/mL using an Amicon Ultra-4 30K centrifugal filter (Millipore Sigma). 80 µL of a screen composed of 0.1 TRIS pH 8.0 and 20% to 30% PEG 4000 (increasing by 0.833% increments was dispensed into the wells of an MRC 2 crystallization plate. 0.3 µL of the well solution was mixed with 0.3 µL of the concentrated tri-complex in a sitting drop and the plate was incubated at 18°C. Crystals grew overnight and to maximum size within 3 days. Crystals vitrified following cryoprotection via mother liquor supplemented with 12.5% glycerol.

#### Crystallography Data Collection and Refinement.

X-ray diffraction data sets were collected at the Stanford Synchrotron Radiation Lightsource (SSRL 9-2; wavelength of 0.979 Å). Data collection and processing were performed as described previously (1). There are no Ramachandran outliers, and 97.1% of residues fall in the favored region. Final processing and refinement statistics can be found in Supplementary Table S7.

### Cell Cultures and Reagents

All cells were purchased from ATCC, ECACC, or JCRB and maintained *in vitro* as a monolayer culture in an appropriate medium supplemented with 10% fetal bovine serum (FBS); some cells required additional supplementary such as penicillin, streptomycin, sodium pyruvate, HEPES buffer, and glucose. All cells were maintained at 37°C in a humidified incubator at 5% CO_2_. Cells in the exponential phase of growth were harvested for tumor cell inoculation.

#### Cell Line Engineering.

LU99 cells (JCRB0080) were stably modified with lentivirus-based pHBLV-CMV-MCS-EF1-fLuc-T2A-puro vector to generate luciferase-expressing LU99-Luc cells. NCI-H1373 cells (ATCC CRL-5866) were stably modified with lentivirus-based pHBLV-CMV-MCS-EF1-fluc-T2A-PURO vector to generate luciferase-expressing NCI-H1373-Luc cells. The eCT26 *Kras*^G12C^^/G12C^*Abcb1*^−^^/^^−^ (clone I20) and eCT26 *Kras*^G12D^^/G12D^*Abcb1*^−^^/^^−^ (clone I12) cell lines were engineered from the murine CT26 homozygous *Kras*^G12D^ tumor cell line (ATCC CRL-2638). All *Kras*^G12D^ alleles were replaced with *Kras*^G12C^ using CRISPR technology at Synthego. Additionally, the P-glycoprotein drug transporter, *Abcb1a* and *Abcb1b*, were knocked out using CRISPR guide sequences TAAGTGGGAGCGCCACTCCA and CCAAACACCAGCATCAAGAG, respectively. The homozygous G12C mutation and the Abcb1 knockout were confirmed by Sanger sequencing in the clone selected and were used for *in vivo* experiments.

#### RMC-6236 Formulation.

For *in vitro* studies, RMC-6236 was resuspended in dimethyl sulfoxide (DMSO) and used at 10 mmol/L stock concentration. For use in *in vivo* studies, RMC-6236 was prepared using formulation of 10/20/10/60 (%v/v/v/v) DMSO/PEG 400/Solutol HS15/water. The same vehicle formulation was used for all control groups.

### *In Vitro* Assays

#### RAS-RAF TR-FRET.

Disruption of the interactions between wild-type KRAS or the mutant oncogenic RAS proteins and the RAS-binding domain of BRAF were assessed by time-resolved fluorescence energy transfer (TR-FRET) in reactions consisting of 12.5 nmol/L His6- KRAS [1–169], 50 nmol/L GST-BRAF [155–229], 10 nmol/L LANCE Eu-W1024 anti-6xHis antibody (PerkinElmer AD0111), 50 nmol/L Allophycocyanin-anti-GST antibody (PerkinElmer AD0059G), and 25 µmol/L CypA in reaction buffer (25 mmol/L HEPES-NaOH pH 7.3, 0.002% Tween20, 0.1% bovine serum albumin, 100 mmol/L NaCl, 5 mmol/L MgCl_2_). Compound or DMSO control (1% v/v) was added and incubated for 1.5 hours, and then TR-FRET was measured on a PerkinElmer Envision plate reader (excitation at 320 nm, 20 µs delay, 100 µs window, 2,000 µs time between flashes; emission at 665 nm and 615 nm in separate channels). The FRET ratio (665/615 nmol/L emission) was used to calculate % Inhibition as: [1 – (FRET ratio of sample – Average FRET ratio of positive controls)/(Average FRET ratio of DMSO control – Average FRET ratio of positive controls)] × 100%.

#### CypA Binding Affinity (K_D_1).

The binding affinity of compounds for CypA was assessed by surface plasmon resonance (SPR) on Biacore 8K instrument. AviTag-CypA was immobilized on a streptavidin sensor chip, and varying compound concentrations were flowed over the chip in assay buffer (10 mmol/L HEPES-NaOH pH 7.4, 150 mmol/L NaCl, 0.005% v/v Surfactant P20, 2% v/v DMSO). The SPR sensograms were fit using either a steady-state affinity model or a 1:1 binding (kinetic) model to assess the K_D_1 for CypA binding.

#### RAS-Binding Affinity (K_D_2).

The binding affinity of compound-bound CypA for the mutant oncogenic RAS proteins mentioned was assessed by SPR on Biacore 8K instrument. AviTag-RAS [1–169] was immobilized on a streptavidin sensor chip, and varying compound concentrations were flowed over the chip in assay buffer (10 mmol/L HEPES-NaOH pH 7.4, 150 mmol/L NaCl, 0.005% v/v Surfactant P20, 2% v/v DMSO, 25 µmol/L CypA). The SPR sensorgrams were fit using either a steady-state affinity model or a 1:1 binding (kinetic) model to assess the *K_D_* for RAS binding.

#### AlphaLISA and MesoScale Discovery (MSD) Analysis of Cellular ERK Phosphorylation.

NCI-H441, Capan-2, HPAC, or isogenic RAS-less MEF cells were seeded in tissue culture–treated 384- and 96-well plates and incubated overnight. The following day, cells were exposed to serial dilutions of compound or DMSO control (0.1% v/v) for specified time points using a Labcyte Echo 550 or Tecan D300e digital dispenser. Following incubation, cells were lysed, and the levels of ERK phosphorylation were determined using the AlphaLISA SureFire Ultra pERK1/2 (T202/Y204) Assay kit (PerkinElmer ALSU-PERK-A50K) or MSD Multi-Array Assay Systems for Phospho/Total ERK1/2 Whole Cell Lysate Kit (K15107D), following the manufacturers’ protocols. Signal was detected using a PerkinElmer Envision with standard AlphaLISA settings, or a Meso QuickPlex SQ120 reader for MSD. For AlphaLISA, data were expressed as % of DMSO-treated control: 100−100 × (pERK_DMSO_ − pERK_treated_)/(pERK_DMSO_ − pERK_media_). MSD signal from pERK1/2 was divided by MSD signal for total ERK1/2. The ratio was normalized to vehicle (% of pERK/total ERK = ((ratio pERK_treated_/total ERK_treated_)/(ratio pERK_DMSO_/total ERK_DMSO_)) × 100). For both assays, data were plotted as a function of log M [compound] with a sigmoidal concentration response (variable slope) model fitted to the data to estimate the inhibitor EC_50_ in Prism 9 (GraphPad).

#### 2D Cell Proliferation Analysis.

NCI-H441, Capan-2, and HPAC cells were seeded in tissue culture–treated 384- or 96-well plates and incubated overnight. Cells were exposed to serial dilutions of compound or DMSO control (0.1% v/v) using a Labcyte Echo 550 or Tecan D300e digital dispenser and incubated for 120 hours at 37°C. Doxycycline-inducible cell lines were retreated with doxycycline at the time of compound treatment. Cell viability was determined by CellTiter-Glo 2.0 reagent (Promega, G9243) according to the manufacturers’ protocols. Luminescence was detected using a SpectraMax M5 Plate Reader (Molecular Devices) of PerkinElmer Enspire. Luminescence signal was normalized to vehicle-treated wells [% vehicle = (lum_treated_/mean(lum_vehicle_) × 100]. Data were plotted as a function of log molar [inhibitor], and a 4-parameter sigmoidal concentration response model was fitted to the data to calculate the EC_50_. Growth percentages were calculated by normalizing the treated cell counts to their respective untreated cell counts.

##### PRISM Assay

###### Cell Lines.

The PRISM cell set comprised 845 cell lines representing more than 45 lineages (see Supplementary Table S1 for cell line information), which largely overlapped with the Cancer Cell Line Encyclopedia; https://portals.broadinstitute.org/ccle). Cell lines were grown in RPMI without phenol red and supplemented with 10% or 20% FBS for adherent and suspended lines, respectively. Parental cell lines were stably infected with a unique 24-nucleotide DNA barcode via lentiviral transduction and blasticidin selection. After selection, barcoded cell lines were expanded and subjected to quality control (*Mycoplasma* contamination test, an SNP test for confirming cell line identity, and barcode ID confirmation). Approved cell lines were then pooled (20–25 cell lines per pool) based on doubling time similarity and frozen in assay-ready vials.

###### PRISM Screening

RMC-6236 was added to 384-well plates at 8-point concentration with 3-fold dilutions in triplicate. These assay-ready plates were then seeded with the thawed cell line pools. Adherent cell pools were plated at 1,250 cells per well, whereas suspension and mixed adherent/suspension pools were plated at 2,000 cells per well. Treated cells were incubated for 5 days, and then lysed. Lysate plates were collapsed together prior to barcode amplification and detection.

###### Barcode Amplification and Detection

Each cell line's unique barcode is located in the 3′UTR of the blasticidin-resistance gene and therefore is expressed as mRNA. Total mRNA was captured using magnetic particles that recognize polyA sequences. Captured mRNA was reverse-transcribed into cDNA and then the sequence containing the unique PRISM barcode was amplified using PCR. Finally, Luminex beads that recognize the specific barcode sequences in the cell set were hybridized to the PCR products and detected using a Luminex scanner which reports the signal as a median fluorescent intensity (MFI).

###### Data Processing

- Each detection well contained 10 control barcodes in increasing abundances as spike-in controls. For each plate, we first create a reference profile by calculating the median of the log_2_(MFI) values across negative control wells for each of these spiked-in barcodes.
- For each well, a monotonic smooth p-spline was fitted to map the spike-in control levels to the reference profile. Next, we transform the log_2_(MFI) for each cell barcode using the fitted spline to allow well-to-well comparisons by correcting for amplification and detection artifacts.
- Next, the separability between negative and positive control treatments was assessed. In particular, we calculated the error rate of the optimum simple threshold classifier between the control samples for each cell line and plate combination. The error rate is a measure of the overlap of the two control sets and was defined as error = (FP + FN)/*n*, where FP is false positives, FN is false negatives, and *n* is the total number of controls. A threshold was set between the distributions of positive and negative control log_2_(MFI) values (with everything below the threshold said to be positive and above said to be negative) such that this value is minimized. Additionally, we also calculated the dynamic range of each cell line. Dynamic range was defined as DR = µ_−_ − µ_+_, where µ+/− stood for the median of the normalized logMFI values in positive/negative control samples.
- From the downstream analysis, we filtered out cell lines with an error rate above 0.05 or a dynamic range less than 1.74. Additionally, any cell line that had less than 2 passing replicates was also omitted for the sake of reproducibility. Finally, we computed viability by normalizing with respect to the median negative control for each plate. Log-fold-change viabilities were computed as log-viability log_2_(x) − log_2_(µ−), where log_2_(x) is the corrected log_2_(MFI) value in the treatment and log_2_(µ−) is the median corrected log_2_(MFI) in the negative control wells in the same plate.
- Log-viability scores were corrected for batch effects coming from pools and culture conditions using the ComBat algorithm^1^.
- We fit a robust four-parameter logistic curve to the response of each cell line to the compound: f(x) = b + (a − b)/(1 + e^s log(x/EC^^50^^)^)
  With the following restrict
  1. We require that the upper asymptote of the curve be between 0.99 and 1.01
  2. We require that the lower asymptote of the curve be between 0 and 1.01
  3. We do not enforce decreasing curves
  4. We initialize the curve-fitting algorithm to guess an upper asymptote of 1 and a lower asymptote of 0.5
  5. When the standard curve fit fails, we report the robust fits provided by the dr4pl R-package
  and computed area under the curve (AUC) values for each dose–response curve and IC_50_ values for curves that dropped below 50% viability.
- Finally, the replicates were collapsed to a treatment-level profile by computing the median log-viability score for each cell line.

#### Associations between Inhibitor Sensitivity AUC and Mutations.

For every gene with nonsilent mutations in at least four cell lines, we compared the AUC values between cells with and without those mutations using a *t* test. This analysis was carried out for (i) the full data set; (ii) excluding cell lines with nonsilent *KRAS* mutations; and (iii) excluding cell lines that have either *KRAS* or *NRAS* nonsilent mutations.

#### Bioinformatics Analyses.

Gene mutation and gene-expression data were downloaded from the 23Q4 release of the DepMap Data Portal11. All qc-filtered compound AUC values were cross-referenced with DepMap Data using an exact matching of the cell line name. For tumor models with no publicly available data, we performed whole-exome sequencing analysis to ascertain gene mutations and RNA sequencing analysis to ascertain gene expression. DNA mutation calling was accomplished with TNSeq using the hg38 version of the human genome. Functional annotation of the resulting mutation calls was accomplished with Variant Effect Predictor and further annotated with oncoKB13. Gene expression was quantified using salmon against the hg38 version of human transcriptome and further processed using txImport and edgeR to generate normalized counts. Copy-number values were downloaded from the DepMap Data portal as log_2_(CN ratio + 1) unless noted otherwise.

#### Cell Panel.

A panel of 78 cancer cell lines harboring mutant and wild-type RAS was selected for screening at Crown Bioscience (Supplementary Table S2). The panel consisted of cell lines with any substitution at position 12 of *KRAS*, cell lines with any substitutions at position 61 of *NRAS*, and cell lines with a *BRAF*^V600E^ mutation. To measure inhibition of cell proliferation/viability and caspase activity, cells were cultured in methylcellulose and treated in triplicate with nine concentrations of RMC-6236 (top concentration of 0.1 µmol/L, 3-fold serial dilutions) or DMSO dispensed by a BioMek FX liquid handler. Cells were incubated for 120 hours prior to measurement of adenosine triphosphate (ATP) levels using the CellTiter-Glo Luminescent Cell Viability Assay (CTG; Promega, G7572), a method of determining the number of metabolically active cells based on quantitation of cellular ATP, according to the manufacturer's instructions. To measure induction of apoptosis, cells were incubated with RMC-6236 for 48 hours prior to measuring cleavage of a proluminescent caspase-3/7 DEVD-aminoluciferin substrate using the CaspaseGlo-3/7 Assay (Promega, G8092) according to the manufacturer’s instructions.

Assay readouts were plotted as a function of log molar [inhibitor] and a 4-parameter sigmoidal concentration response model was fitted to the data to estimate the inhibitor EC_50_ for CTG (half maximal concentration of RMC-6236) and EC_200_ for CaspaseGlo-3/7 (concentration of RMC-6236 that induces a 2-fold increase in CaspaseGlo-3/7 signal relative to DMSO-treated control cells) using Genedata Screener.

#### Western Blot Analysis.

Cells were seeded at 0.3 to 2.0 million cells per well of tissue culture–treated 6-well plates. After overnight incubation, compounds or DMSO (0.1% v/v) was added and incubated for the indicated time points. Cells were washed twice with ice-cold PBS and lysed with MSD Tris Lysis Buffer (MSD, R60TX-2), and scraped and collected before centrifugation. Tumor tissues were dissected and cut into 50 to 100 mg fragments, then snap-frozen, and then lysed with NP-40 Cell Lysis Buffer (Invitrogen, FNN0021) before homogenization with tissue grinder (Scientz, Scientz-48). All lysis buffers were supplemented with protease and phosphatase inhibitors. Lysates were centrifuged at 21,000 × *g* for 10 minutes at 4°C. The protein-containing supernatants were quantified by BCA assay (Pierce, 23225), and equal quantities of protein were denatured with LDS and reducing agent for 10 minutes at 95°C (for cell lysates) or 75°C (for tissue lysates). Samples were resolved on 4% to 12% Bis-Tris polyacrylamide gels and then subjected to Western blot.

The following primary antibodies were used at 1:1,000 to 1:2,000 dilutions: anti–phospho-p44/42 MAPK (ERK1/2) T202/Y204 (no. 9101), anti-p44/42 (ERK1/2; no. 9107), pAKT S473 (no. 4060), AKT (no. 2920), pS6 S235/236 (no. 2211), S6 (no. 2317), PARP (no. 9542), β-Actin (no. 4967, 1:2,000 diluted), and vinculin (no. 13901) all from Cell Signaling Technology; anti-RAS (Abcam, ab108602) and anti-KRAS (Sigma-Aldrich, WH0003845M1). The following secondary antibodies were used as appropriate: goat anti-rabbit IR800-conjugated (LI-COR, 926-32211), goat anti-mouse IR680-conjugated (LI-COR, 926-68070), donkey anti-rabbit IR800-conjugated (LI-COR, 926-32213), donkey anti-mouse IR680-conjugated (LI-COR, 926-68072), goat anti-rabbit IgG (H + L) HRP-conjugated (Thermo Fisher Scientific, 31462) or goat anti-mouse IgG (H + L; Thermo Fisher Scientific, A16072) HRP-conjugated.

### *In Vivo* Studies

#### Animal Studies Using Xenograft Tumor Models.

Studies were conducted at the following contract research organizations (CROs): GenenDesign, Pharmaron, Wuxi AppTec, Champions Oncology, Charles River Laboratories, and XenoSTART. All CDX/PDX mouse studies and procedures related to animal handling, care, and treatment complied with all applicable regulations and guidelines of the Institutional Animal Care and Use Committee (IACUC) at each facility with their approvals. Female BALB/c nude, NOD-SCID, NMRI nu/nu, and athymic nude mice 6 to 12 weeks old were used. Animal vendors include Beijing Vital River/VR Laboratory Animal Co. LTD., Beijing AniKeeper Biotech Co. Ltd., Shanghai Sino-British SIPPR/BK Laboratory Animal Co. LTD., and Charles River Laboratories.

#### Generation of Xenograft Models.

To generate subcutaneous CDX, each mouse was inoculated at the right flank with tumor cells (2 × 10^6^ − 1 × 10^7^) in 100 to 200 µL of media/PBS Supplemented with Matrigel. Treatments were initiated when average tumor volume reached 130 to 200 mm^3^ for tumor growth evaluation and 350 to 650 mm^3^ for single-dose pharmacokinetic/pharmacodynamic (PK/PD). Tumor diameter was measured in two dimensions using a digital caliper, and the tumor volume in mm^3^ was calculated using the formula: Volume = ((width)^2^ × length)/2. Mice in the study were weighed and tumors were measured twice weekly.

The human primary cancer PDX models were generated using fresh tumor fragments obtained from the hospital with written informed consent from patients in accordance with protocols approved by the Hospital's Institutional Ethical Committee. Tumor fragments were subcutaneously serial passaged in immunodeficient mice and cryopreserved for further use. Recovered tumor fragments were implanted into the right flanks of immunodeficient mice; treatment started when the average tumor volume reached 150 to 350 mm^3^.

#### Intracranial Tumor Model.

NCI-H1373-Luc cells (3 × 10^5^) were prepared in 3 µL sterile PBS with 20% Matrigel. Each mouse was anesthetized by intraperitoneal (ip) injection of sterile avertin (250 mg/kg) and positioned on the stereotaxic unit. Cells were intracranially injected over 2 minutes at the site 2 mm lateral (right), 0.5 mm anterior, and 3 mm ventral with respect to the bregma. Meloxicam (2 mg/kg) was administered subcutaneously to relieve pain post-surgery for three consecutive days. Study mice were weighed and intraperitoneally administered with luciferin at 150 mg/kg on the day of measurement. Ten minutes after the luciferin injection, animals were anesthetized and moved into the imaging chamber for bioluminescence measurements with an IVIS (Lumina II) imaging system. The bioluminescence and animal weights were measured and recorded once and twice per week, respectively. The tumor growth curve is plotted using bioluminescence intensity (photons/sec) as a surrogate measurement for tumor size.

#### RMC-6236 Treatment.

Tumor-bearing animals were randomized and assigned into groups (*n* = 1–10/group). The vehicle at 10 mL/kg or RMC-6236 at indicated doses was administered via oral gavage daily, and animals were treated for 28 days, or up to 90 days if PFS was being assessed. Animals were terminated early if the tumor burden reached a humane endpoint, or adverse effect was observed with body weight loss as a surrogate. For single-dose PKPD study, mice were randomized and assigned into groups (*n* = 3/dose/time point). A single dose of RMC-6236 was administered orally at either 3, 10, or 25 mg/kg. Blood and tissues, including the tumor, brain, colon, ear skin, and muscle, were harvested at indicated time points. Whole blood was collected in K_2_EDTA Microtainer tubes, incubated for 5 minutes, and snap-frozen in liquid nitrogen. The tissue was either fixed in 10% formalin or snap-frozen in liquid nitrogen for further analysis.

#### *In Vivo* Study Data Analysis.

In tumor volume plots, the average tumor volume of each group was plotted over the course after implantation (except for PDX models, where the x-axis started on the first day of treatment). Control and RMC-6236 groups were compared by two-way repeated-measures ANOVA on the last measurement day of the control group. Percentage change in body weight for each animal on a given day was determined as [(body weight on test article administration end date/body weight on test article administration start date) −1] × 100. The percentage mean tumor volume change from baseline was graphed in the waterfall plots. The mRECIST score was determined based on % mean tumor volume change, mCR is more than 80% regression, mPR is between 30% and 80% regression, mSD is between 30% growth and 30% regression, mPD is more than 30% growth. Progression is defined as tumor volume doubling from baseline and represented with Kaplan–Meier plots. Log-rank test was used to compare vehicle control with treatment groups. Cox Proportional Hazards models were used to estimate hazard ratios between vehicle control and treatment groups. Tumor relapse was defined here as models that were considered mPR or mCR at the response calling date and then rebounded from mean tumor volume regressions to either stable disease or progression when dosed long-term (more than 60 days).

#### *In Vivo* Pharmacodynamic Analysis by *DUSP6* qPCR.

RNA was extracted from at least 20 mg of tissue using an RNeasy Mini Kit (Qiagen, 74104) and a High-Throughput Tissue grinder following the manufacturer's protocol. Reverse transcription was carried out using High-Capacity cDNA Reverse Transcription Kit (ABI, 4368814) according to the manufacturer's protocol. The cDNA product was used for qPCR analysis using TaqMan Gene-Expression Master Mix (ABI, 4369016). TaqMan primer probes specific to *DUSP6* (human—Hs00737962_m1, murine—Mm00518185_m1, FAM-MGB) and *18S* (*RNA18S1*; human—Hs99999901_s1, murine—Mm03928990_g1, FAM-MGB, used as an internal control gene) were used to detect the levels from each sample in duplicates using a 10 µL final reaction volume in a 384-well clear optical reaction plate. For qPCR, Ct values of *DUSP6* and *18S* were obtained for analysis. *DUSP6* Ct value was normalized to *18S*, and then the mean relative mRNA expression levels of each group were normalized to the vehicle control group. Values were plotted as relative change in mRNA expression compared with vehicle. Means ± SEM were shown.

#### Droplet Digital PCR.

gDNA was extracted from at least 20 mg of tumor tissue using a QIAamp DNA Mini Kit (Qiagen, cat no. 51304). 10 ng of gDNA was included for droplet digital PCR (ddPCR) using the Naica system multiplex digital PCR (Stilla) per the manufacturer’s protocol. Probes and primers for the following genes were included in the multiplexed ddPCR: *KRAS*^WT^ [dPCR Mutation Detection Assay KRAS Wild-Type for p.G12C, Human (apexbio, AA100902-WT)], *KRAS*^G12C^ [dPCR Mutation Detection Assay KRAS Mutant for p.G12C, Human (apexbio, AA100902-MU)], and *ACTB* (*ACTB* probe, 5′-Cy5-ATTGCCGACAGGATGCAGAAGGA-3′; *ACTB* primer F, 5′-GACATCCGCAAAGACCTGTA-3′; *ACTB* primer R, 5′-GGAAAGACACCCACCTTGAT-3′). For copy-number assessment, the results were normalized to *ACTB*.

#### Mouse Blood and Tissue Sample Bioanalysis.

The whole blood, tumor, brain, colon, and ear skin concentrations of RMC-6236 were determined using liquid chromatography–tandem mass spectrometry (LC/MS-MS) methods. Tissue samples were homogenized with a 10 × volume of homogenization buffer [methanol/15 mmol/L PBS (1:2; v:v) or 15 mmol/L PBS with 10% methanol]. An aliquot of whole blood or homogenized tissue (10, 20, or 40 µL) was transferred to 96-well plates (or tubes) and quenched with a 10 × volume of acetonitrile or 20 × volume of acetonitrile/methanol (1:1; v/v) with 0.1% formic acid containing a cocktail of internal standards (IS). After thorough mixing and centrifugation, the supernatant was diluted with water or directly analyzed on a Sciex 5500 or Sciex 6500+ triple quadrupole mass spectrometer equipped with an ACQUITY or Shimadzu UPLC system. A Halo 90Å AQ-C18 2.7 µm (2.1 × 50 mm) or an ACQUITY UPLC BEH C18 or C4 1.7 µm (2.1 × 50 mm) column was used with gradient elution for compound separation. RMC-6236 and IS (verapamil, celecoxib, glyburide, dexamethasone, or terfenadine) were detected by positive electrospray ionization using multiple reaction monitoring (RMC-6236: m/z 811/779; verapamil: m/z 455/165; celecoxib: m/z 382/362; glyburide: m/z 494/169; dexamethasone: m/z 393/373; terfenadine: m/z 472/436). The lower limit of quantification was 1 ng/mL or 2 ng/mL for blood, tumor, and other tissue. Bioanalysis on blood and tissue samples from xenograft models was run at Pharmaron and Wuxi AppTec.

#### PK Analysis.

Concentrations reported as below the quantification limit were treated as zero for PK analysis. PK parameters were calculated by noncompartmental analysis of the concentration–time profiles using Phoenix WinNonLin (version 8.3 Certara). Apparent terminal elimination half-life (t_1/2_) values were calculated as ln(2)/k, where k represents the terminal elimination rate constant. Area under the concentration–time curve (AUC) values were estimated using a linear trapezoidal method. AUC_last_ values were calculated from the dosing time to the last quantifiable concentration. Maximum concentration (C_max_) was recorded as observed.

#### Immunohistochemistry.

All tissues were fixed for up to 24 hours using 10% neutral buffered formalin and then moved to 70% ethanol for long-term storage. FFPE sections (4 µm) were stained on the Biocare intelliPATH automated staining platform using the manufacturer’s recommended settings.

*Anti-EpCAM* rabbit monoclonal antibody (Cell Signaling Technology, cat no. 14452, clone: D9S3P) was used at 1:200 with citrate-based pH 6.2 Heat-Induced Epitope Retrieval; an isotype control (rabbit IgG) was used under the same conditions.

*Anti–Ki-67* rabbit monoclonal antibody (Biocare, cat no. CRM325, clone: SP6) was used at 1:50 with citrate-based pH 6.2 heat-induced epitope retrieval; an isotype control (rabbit IgG) was used under the same conditions.

*Anti–phospho-p44/42 MAPK (Erk1/2; Thr202/Tyr204)* rabbit monoclonal antibody (Cell Signaling Technology, cat no. 4370, clone: D13.14.4E) was used at 1:200 with citrate-based pH 6.2 heat-induced epitope retrieval; an isotype control (rabbit IgG) was used under the same conditions.

*Anti–phospho-histone H3 (Ser10)* rabbit polyclonal antibody (Cell Signaling Technology, cat no. 9701, Lot # 17) was used at 1:200 (0.1 µg/mL) with citrate-based pH 6.2 heat-induced epitope retrieval; an isotype control (rabbit IgG) was used under the same conditions.

*Anti–phospho-S6 ribosomal protein (Ser235/236)* rabbit monoclonal antibody (Cell Signaling Technology, cat no. 4858, clone: D57.2.2E) was used at 1:200 with citrate-based pH 6.2 heat-induced epitope retrieval; an isotype control (rabbit IgG) was used under the same conditions. FFPE sections (4 µm) were stained on the Biocare intelliPATH automated staining platform using the manufacturer's recommended settings.

All tissue sections were incubated with Biocare Peroxidase Blocker (Biocare, cat no. PX968) and Background Punisher (Biocare, cat no. BP974M) to block nonspecific background. MACH4 HRP-polymer Detection System (Biocare, cat no. MRH534) was used to detect rabbit primary antibodies.

DAB-stained slides were scanned and digitized with a Huron TissueScope LE120 whole-slide scanner at 200× magnification.

#### Whole-Slide Image Analysis.

The EpCAM staining was used to identify epithelial cells. First, the area to be analyzed was delineated, excluding necrotic regions. The random forest tumor classifier from the HALO Image Analysis package was used to identify the tumor compartment. Three classes were created: glass, tumor (EpCAM-positive), and stroma. The tumor class mark was then copied onto the serial sections stained with various markers to perform the image analysis only on the tumor compartment.

Quantification of Ki-67 was performed with the HALO Image Analysis software from Indica Labs using the CytoNuclear module. The analysis was performed only on the tumor compartment copied from the EpCAM slide. The software was tuned to detect all the nuclei based on the hematoxylin stain (blue color) and to detect positive DAB staining (brown color). Total positivity was plotted and subjected to statistical analysis using GraphPad Prism (Dunnett multiple comparisons test).

Quantification of P-ERK and pS6 was performed with the HALO Image Analysis software from Indica labs using the Area Quantification module. The analysis was performed only on the tumor compartment copied from the EpCAM slide. The software was tuned to detect positive DAB staining (brown color). Percentage of area positivity was chosen to represent the results (area positive for brown/total area); and subjected to statistical analysis using GraphPad Prism (Dunnett multiple comparisons test).

#### PK/PD Relationship.

Concentrations of RMC-6236 in tumor or normal tissues and percentage of *DUSP6* inhibition as compared with the vehicle control from individual animals were collected and analyzed post a single dose of RMC-6236 ranging from 0.3 to 100 mg/kg (Supplementary Table S6). A 3-parameter sigmoidal exposure–response model was fitted to the data in GraphPad Prism to derive EC_50_ and EC_90_ values.

#### PK/Efficacy and PK/PD Modeling.

PK/Efficacy and PK/PD models were built sequentially by first fitting observed blood PK parameters and subsequently incorporating either tumor growth inhibition or tumor PK/PD parameters. Akaike information criterion values were used to discriminate between model structure and fit. Individual data points (*n* = 3/time point) were averaged from sparsely sampled PK/PD data to create mean profiles, which were used for subsequent modeling. A graphical representation of the model and all parameter estimates and equations can be found in the Supplementary Information (Supplementary Fig. S6; Supplementary Table S8l Supplementary Method).

For PK modeling, whole blood PK data from single or repeat dose administration of 25 or 40 mg/kg RMC-6236 to NCI-H441 xenograft tumor-bearing mice were used (Supplementary Table S9). RMC-6236 blood PK was best described using a one-compartment model with first-order absorption and elimination. Because intravenous data were not included in the modeling, the model was parameterized in terms of apparent clearance (CL/F) and volume of distribution (V/F), where F is the oral bioavailability.

Tumor growth was modeled using the approach previously described by Simeoni and colleagues (45). As detailed previously, tumor volume data were collected from mice bearing either NCI-H441 or Capan-2 xenograft tumors in 28-day efficacy studies (Supplementary Table S9). Estimated intrinsic tumor growth parameters include initial tumor volume, W0, which was set to the observed initial tumor volume, and the exponential and linear growth rates constants, λ_0_ and λ_1_, which were estimated from vehicle-treated animal tumor growth. Drug-specific parameters include a measure of drug potency, k_2_, and a transfer rate constant, k_1_, which describes cell death kinetics. A tumor stasis concentration threshold (C_T_) was calculated as λ_0_/k_2_ and converted to a human equivalent by adjusting for species differences in blood-plasma partitioning and plasma protein binding as discussed in Supplementary Methods. Human PK parameters were predicted via multispecies allometric scaling or assigned as the preclinical average and used to simulate mean steady-state human exposure at various dose levels.

The mouse whole-blood PK model was expanded to include tumor PK/PD by incorporating blood–plasma partitioning, plasma protein binding, and tumor partitioning and PD parameters. PK/PD modeling was conducted using data from NCI-H441 xenograft tumor-bearing mice due to greater data availability. RMC-6236 tumor concentrations and *DUSP6* mRNA expression were measured in tumor tissue after single or repeat dose administration of 25 or 40 mg/kg to NCI-H441 xenograft tumor-bearing mice (Supplementary Table S9). These data were used to fit and refine tumor PK/PD parameters and then validated against data collected from single-dose administration of 10, 25, or 50 mg/kg RMC-6236 to NCI-H441 xenograft tumor-bearing mice. The PD response was modeled using *DUSP6* biomarker data from NCI-H441 mouse xenograft studies. Blood concentrations did not directly correlate with tumor PD; however, tumor concentrations were generally well correlated. At early time points, tumor concentrations could not directly account for the observed *DUSP6* modulation, suggesting that a time delay must be incorporated into the PD model. As such, an indirect response model was used to describe the suppression of *DUSP6* expression, assuming zero-order production, first-order degradation, and maximal inhibition fixed at 100%.

The mouse PK/PD model was scaled to humans by incorporating human-specific plasma protein binding, blood–plasma partitioning, and estimated human blood PK parameters. The tumor partitioning rate constants were held constant from the mouse model under the assumption that RMC-6236 will partition similarly from free plasma into human-derived xenografts or clinical tumor tissue. Similarly, parameters governing *DUSP6* expression were kept the same and assumed to reflect a similarly sensitive tumor in humans. Human simulations were conducted for multiple clinical dose levels using mean PK parameters and assuming an average body weight of 70 kg.

### Animal Studies Using Syngeneic Models and Genetically Engineered Mouse Models (GEMMs)

Studies were conducted at Revolution Medicines and D2G Oncology. Studies were approved by the IACUC. All studies were conducted in compliance with the facility's animal welfare body guidelines and animal use protocols.

### Syngeneic Model Study

Female BALB/c immunocompetent mice and female NOD-SCID/IL2Rg(null; NSG) immune-deficient mice (6–8 weeks old) were purchased from the Jackson Laboratory and were acclimated at Revolution Medicines for 7 days before cell implantation. Each mouse was inoculated subcutaneously at the right flank with eCT26 *Kras*^G12C/G12C^ clone I20 cancer cells (5 × 10^6^ live cells) or eCT26 *Kras*^G12D/G12D^ clone I12 cancer cells (3 × 10^6^ live cells) in 0.1 mL of serum-free RPMI-1640 for tumor development (day 0 is the day of cell implantation). Treatments were initiated at the time of randomization when the average tumor volume reached 100 to 120 mm^3^. The animals were assigned into groups using Studylog randomization software, performing stratified randomization based on their tumor volumes. In combination studies, anti–PD-1 (clone RMP1-14, anti-mouse PD-1 rat IgG2a) was dosed intraperitoneally at 10 mg/kg biweekly for a total of six doses.

### Flow-Cytometric Analysis

For the TME analysis, tumor tissue was collected 24 hours post the last dose after 4 days of treatment with vehicle or RMC-6236 and was processed for flow-cytometric analysis. The tumor volume average for the vehicle group on day 0 was 278 ± 18 mm^3^, and on day 4 it was 535 ± 115 mm^3^. For the RMC-6236–treated group, the tumor average on day 0 was 445 ± 163 mm^3^, and it regressed by day 4 to 71 ± 32 mm^3^. Tumor tissue was minced, processed with the Dri Tumor & Tissue Dissociation Reagent from BD Biosciences and homogenized with the gentleMACS Dissociator. Tumor cell suspensions were incubated at 4°C for 30 minutes with Mouse BD Fc Block (Clone 2.4G2 from BD Pharmingen), 10 minutes with Blue Dead Cell Stain Kit (from Invitrogen) and 30 minutes in cell staining buffer. Antibodies used targeted CD45 (clone 30F11), CD19 (clone 1D3), CD8b (clone H35-17.2), Ly-6G (clone 1A8), I-A/I-E (clone M5/114.15.2) from BD and CD3ε (clone 145-2C11), CD4 (clone GK1.5), CD11b (clone M1/70), F4/80 (clone BM8), and Ly-6C (clone HK1.4) from BioLegend. Cells were analyzed on a 4-laser Cytek Aurora (Cytek Biosciences), and data analysis was done using SpectroFlo (Cytek Biosciences) and FlowJo (FlowJo LLC).

### Generation, Treatment, and Analysis of Autochthonous Tumors in GEMMs of Lung Cancer

For *in vivo* experiments in autochthonous mouse models of oncogenic *Kras*-driven NSCLC (Fig. 4A; Supplementary Fig. S4A), tumors were initiated via intratracheal delivery of lentivirus to the lungs of mice as previously described (35). Specifically, to understand the responsiveness of tumors harboring diverse oncogenic *Kras* variants to RMC-6236 treatment, lung tumors were initiated in B6 mice using a barcoded lentivirus pool including vectors encoding oncogenic KRAS mutant (G12C, G12V, G12D, G12A, Q61H, or G13D) cDNAs (Lenti;Kras^MUT^;BC). Thirteen weeks post tumor initiation, mice were treated for 3 weeks with either: (i) vehicle (10% DMSO, 20% PEG400, 10% Solutol HS15, 60% water) po qd and 10 mg/kg isotype rat igg2a[2a3] ip biw; or (ii) RMC-6236 20 mg/kg po qd. Effects were captured and quantified by extraction of genomic DNA from tumor-bearing lung tissue, followed by PCR amplification and ultra-deep Illumina sequencing of tumor barcodes, and analysis of sequencing data as previously described (35).

Figure 4A and Supplementary Fig. S4A show confidence intervals for the relative tumor burden (RTN) of treated versus vehicle tumors of each genotype. Briefly, for each tumor genotype, we computed the median tumor burden (the sum of the neoplastic cell counts of all observed tumors) across mice in the treated and vehicle-treated arms. From these, a percentage change between vehicle-treated and treated was computed. This procedure was repeated on 1,000 bootstraps, constructed using the same procedure used for RTN score (see above). The bootstrap distribution of percentage changes is presented with boxplots.

### Data Availability

Data used to generate analyses and visualization in this publication are available within the article and its Supplementary data files.

Revolution Medicines will not provide access to patient-level data if there is a reasonable likelihood that individual patients could be reidentified.

### Clinical Trials

The RMC-6236-001 clinical trial (NCT05379985) is being conducted in accordance with recognized U.S. ethical guidelines (i.e., U.S. Common Rule) and per local institutional review board guidelines. All patients included in the clinical trial were subject to and provided written informed consent prior to study enrollment. RMC-6236 was administered once daily in 21-day cycles to patients enrolled on the protocol.

## Supplementary Material

Supplementary Method RMC-6236 synthetic route and detailed PK/PD/Efficacy Modeling method

Supplementary Table S1 Supplementary Table S1 shows information on the PRISM cell set comprised of 845 cell lines representing more than 45 lineages.

Supplementary Table S2 Supplementary Table S2 shows cell panel screening data with a panel of 78 cancer cell lines harboring mutant and wild-type RAS selected for screening at Crown Bioscience.

Supplementary Table S3 Supplementary Table S3 shows PK and/or PD data of RMC-6236 in subcutaneous xenograft tumors, intracranially implanted xenograft tumors, normal brain from naüve mice, and ear skin and colon from xenograft tumor bearing mice.

Supplementary Table S4 Supplementary Table S4 shows % mean tumor volume change, % mean body weight change of RMC-6236 treatment group on mRECIST response calling date; and PPIA mRNA expression levels of each xenograft model in RMC-6236 mouse clinical trial.

Supplementary Table S5 Supplementary Table S5 shows time to tumor doubling of individual animals in RMC-6236 mouse clinical trial.

Supplementary Table S6 Supplementary Table S6 shows PK/PD data used to derive PK/PD relationship curves in Fig. 5 and Fig. S5.

Supplementary Table S7 Supplementary Table S7 showes final processing and refinement statistics used for RMC-6236 Crystallography data collection and refinement.

Supplementary Table S8 Supplementary Table S8 shows all parameter estimates for PK/Efficacy and PK/PD modeling.

Supplementary Table S9 Supplementary Table S9 shows datasets used for PK/Efficacy and PK/PD modeling.

Supplementary Figure S1-S6 Supplementary Figure 1 shows RMC-6236 crystal structure in tri-complex, as well as biophysical and cellular potencies of RMC-6236 by genotype.Supplementary Figure 2 shows RMC-6236 demonstrates dose-dependent anti-tumor activities at tolerable doses; and pharmacodynamic effects on RAS signaling in NCI-H441 xenograft tumors as assessed by IHC, and in relatively refractory KP-4 and NCI-H2122 xenograft tumors as assessed by human DUSP6 mRNA expression in vivo.Supplementary Figure 3 shows genotype dependent response of RMC-6236 across NSCLC, PDAC and CRC; and potential modifiers to the durability of response of KRASG12C NSCLC models upon RMC-6236 treatment assessed by Kaplan-Meier analyses.Supplementary Figure 4 shows Efficacy of RMC-6236 on KrasG12C–driven autochthonous lung tumors harboring cis second-site mutations within KrasG12C (KrasG12C,H95D or KrasG12C,Y96C) and eCT26 (KrasG12D/G12D) syngeneic model in immunocompetent mice; anti-tumor immunity of RMC-6236; and in intracranially implanted NCI-H1373-Luc xenograft model on nude mice.Supplementary Figure 5 shows effects of RMC-6236 mediated pharmacological modulation in KP-4 xenograft tumors and normal colon tissues isolated from xenograft tumor bearing mice.Supplementary Figure 6 shows a graphical representation of the combined mouse PK-Efficacy and PK/PD model.
