# Correction: Translational and Therapeutic Evaluation of RAS-GTP Inhibition by RMC-6236 in RAS-Driven Cancers

**DOI:** 10.1158/2159-8290.CD-25-1519

**Published:** 2025-10-06

**Authors:** Jingjing Jiang, Lingyan Jiang, Benjamin J. Maldonato, Yingyun Wang, Matthew Holderfield, Ida Aronchik, Ian P. Winters, Zeena Salman, Cristina Blaj, Marie Menard, Jens Brodbeck, Zhe Chen, Xing Wei, Michael J. Rosen, Yevgeniy Gindin, Bianca J. Lee, James W. Evans, Stephanie Chang, Zhican Wang, Kyle J. Seamon, Dylan Parsons, James Cregg, Abby Marquez, Aidan C.A. Tomlinson, Jason K. Yano, John E. Knox, Elsa Quintana, Andrew J. Aguirre, Kathryn C. Arbour, Abby Reed, W. Clay Gustafson, Adrian L. Gill, Elena S. Koltun, David Wildes, Jacqueline A.M. Smith, Zhengping Wang, Mallika Singh

In the original version of this article (1), LXFA2250 was duplicated in Fig. 3A by the compositor during production of the paper, resulting in misalignment of the NSCLC xenograft model names. The publisher regrets this error. In the “Cell Panel” section of the Methods, the text should read: “To measure inhibition of cell proliferation/viability, cells were cultured in methylcellulose and treated in triplicate with nine concentrations of RMC-6236 (top concentration of 0.1 μmol/L, 3-fold serial dilutions) or DMSO dispensed by a BioMek FX liquid handler,” and additional information about the assay format was omitted in the original version of the article. The authors regret these errors. The errors have been corrected in the latest online HTML and PDF versions of the article.
