# Supplementary material for: Translational and Therapeutic Evaluation of RAS-GTP Inhibition by RMC-6236 in RAS-Driven Cancers
Source: Cancer Discov. 2024 Apr 9;14(6):994–1017. doi: 10.1158/2159-8290.CD-24-0027 (PMC11149917; doi:10.1158/2159-8290.CD-24-0027)
Supplement: Supplementary Method — RMC-6236 synthetic route and detailed PK/PD/Efficacy Modeling method [file cd-24-0027_supplementary_method_suppst0.docx]

**Supplementary Methods**

**Synthesis of RMC-6236**

*(S)-4-(5-bromo-6-(1-methoxyethyl)pyridin-3-yl)piperazine-1-carboxylate* (**T1**) Into a 3-L 3-necked round-bottom flask purged and maintained with an inert atmosphere of argon, was placed 3-bromo-5-iodo-2-[(1*S*)-1-methoxyethyl]pyridine (147 g, 429.8 mmol) benzyl piperazine-1-carboxylate (94.69 g, 429.8 mmol), Pd(OAc)_2_ (4.83 g, 21.4 mmol), BINAP (5.35 g, 8.6 mmol), Cs_2_CO_3_ (350.14 g, 1074.6 mmol), Toluene (1 L). The resulting solution was stirred for overnight at 100 °C in an oil bath. The reaction mixture was cooled to 25 °C after reaction completed. The resulting mixture was concentrated under reduced pressure. The residue was applied onto a silica gel column with ethyl acetate/hexane (1:1). Removal of solvent under reduced pressure gave benzyl (*S*)-4-(5-bromo-6-(1-methoxyethyl)pyridin-3-yl)piperazine-1-carboxylate (135 g, 65.1% yield) as a dark yellow solid. ^1^H NMR (400 MHz, DMSO-*d*_6_) δ 8.33 (d, *J* = 2.5 Hz, 1H), 7.60 (d, *J* = 2.5 Hz, 1H), 7.42 – 7.30 (m, 5H), 5.12 (s, 2H), 4.75 (q, *J* = 6.4 Hz, 1H), 3.55 (t, *J* = 4.8 Hz, 4H), 3.29 (t, *J* = 5.2 Hz, 4H), 1.37 (d, *J* = 6.4 Hz, 3H). ^13^C NMR (101 MHz, Chloroform-*d*) δ 155.03, 147.07, 146.23, 136.34, 133.11, 128.66, 128.55, 128.18, 127.91, 120.82, 76.30, 67.43, 57.02, 47.27, 43.03, 20.05. LCMS (ESI): m/z [M+H] calc’d for C_20_H_24_BrN_3_O_3_ 433.1; found 434.1.

*(S)-4-(6-(1-methoxyethyl)-5-(4,4,5,5-tetramethyl-1,3,2-dioxaborolan-2-yl)pyridin-3-yl)piperazine-1-carboxylate* (**T2**) Into a 3-L 3-necked round-bottom flask purged and maintained with an inert atmosphere of argon, was placed benzyl 4-[5-bromo-6-[(1*S*)-1-methoxyethyl]pyridin-3-yl]piperazine-1-carboxylate (135 g, 310.8 mmol), bis(pinacolato)diboron (86.82 g, 341.9 mmol), Pd(dppf)Cl_2_ (22.74 g, 31.0 mmol), KOAc (76.26 g, 777.5 mmol), Toluene (1 L). The resulting solution was stirred for 2 days at 90 °C in an oil bath. The reaction mixture was cooled to 25 °C. The resulting mixture was concentrated under vacuum. The residue was applied onto a [neutral alumina](http://dict.youdao.com/search?q=neutral%20alumina%0D%0A&keyfrom=fanyi.smartResult) column with ethyl acetate/hexane (1:3). Removal of solvent under reduced pressure gave benzyl (*S*)-4-(6-(1-methoxyethyl)-5-(4,4,5,5-tetramethyl-1,3,2-dioxaborolan-2-yl)pyridin-3-yl)piperazine-1-carboxylate (167 g, crude) as a dark yellow solid. ^1^H NMR (400 MHz, DMSO-*d*_6_) δ 8.27 (d, *J* = 3.0 Hz, 1H), 7.98 (s, 1H), 7.43 – 7.29 (m, 6H), 5.12 (s, 2H), 4.56 (dq, *J* = 31.3, 6.5 Hz, 1H), 3.55 (q, *J* = 10.2, 7.5 Hz, 4H), 3.17 (q, *J* = 5.2, 3.9 Hz, 5H), 3.09 (s, 2H), 1.38 (dd, *J* = 12.4, 6.5 Hz, 3H), 1.31 (s, 6H). ^13^C NMR (101 MHz, DMSO-*d*_6_) δ 154.88, 145.10, 138.94, 137.31, 128.88, 128.86, 128.32, 128.07, 128.03, 84.11, 80.26, 79.15, 66.81, 56.31, 56.25, 48.49, 48.30, 25.21, 24.96, 21.34, 20.73. LCMS (ESI): m/z [M+H] calc’d for C_26_H_36_BN_3_O_5_ 481.3; found 482.1.

*benzyl (S)-4-(5-(5-bromo-3-(3-((tert-butyldiphenylsilyl)oxy)-2,2-dimethylpropyl)-1H-indol-2-yl)-6-(1-methoxyethyl)pyridin-3-yl)piperazine-1-carboxylate* (**T3**) Into a 3-L 3-necked round-bottom flask purged and maintained with an inert atmosphere of argon, was placed (*S*)-4-(6-(1-methoxyethyl)-5-(4,4,5,5-tetramethyl-1,3,2-dioxaborolan-2-yl)pyridin-3-yl)piperazine-1-carboxylate (167 g, 346.9 mmol), 5-bromo-3-[3-[(tert-butyldiphenylsilyl)oxy]-2,2-dimethylpropyl]-2-iodo-1H-indole (224.27 g, 346.9 mmol), Pd(dppf)Cl_2_ (25.38 g, 34.6 mmol), dioxane (600 mL), H_2_O (200 mL), K_3_PO_4_ (184.09 g, 867.2 mmol), Toluene (200 mL). The resulting solution was stirred for overnight at 70 °C in an oil bath. The reaction mixture was cooled to 25 °C after reaction completed. The resulting mixture was concentrated under vacuum. The residue was applied onto a silica gel column with ethyl acetate/hexane (1:1). Removal of solvent under reduced pressure gave benzyl (*S*)-4-(5-(5-bromo-3-(3-((*tert*-butyldiphenylsilyl)oxy)-2,2-dimethylpropyl)-1*H*-indol-2-yl)-6-(1-methoxyethyl)pyridin-3-yl)piperazine-1-carboxylate (146 g, 48.1% yield) as a yellow solid. ^1^H NMR (400 MHz, DMSO-*d*_6_) δ 11.32 (s, 1H), 8.43 (d, *J* = 2.8 Hz, 1H), 7.77 (d, *J* = 1.9 Hz, 1H), 7.57 – 7.47 (m, 4H), 7.46 – 7.29 (m, 12H), 7.25 (d, *J* = 2.9 Hz, 1H), 7.21 (dd, *J* = 8.6, 1.9 Hz, 1H), 5.12 (s, 2H), 4.10 (q, *J* = 6.2 Hz, 1H), 3.52 (t, *J* = 5.2 Hz, 4H), 3.18 (d, *J* = 5.7 Hz, 4H), 2.87 (s, 3H), 2.51 (m, 2H), 1.44 – 1.20 (m, 4H), 0.96 (s, 9H), 0.65 (d, *J* = 7.5 Hz, 6H). ^13^C NMR (101 MHz, Chloroform-*d*) δ 149.20, 137.38, 136.44, 135.73, 135.71, 134.19, 133.83, 131.64, 129.64, 129.10, 128.63, 128.28, 128.11, 127.67, 125.82, 125.03, 122.67, 112.43, 111.48, 73.22, 67.49, 55.32, 48.28, 39.09, 32.57, 27.12, 24.45, 24.32, 19.51. LCMS (ESI): m/z [M+H] calc’d for C_49_H_57_BrN_4_O_4_Si 872.3; found 873.3.

*benzyl (S)-4-(5-(5-bromo-3-(3-((tert-butyldiphenylsilyl)oxy)-2,2-dimethylpropyl)-1-ethyl-1H-indol-2-yl)-6-(1-methoxyethyl)pyridin-3-yl)piperazine-1-carboxylate* (**T4**) To a stirred mixture of benzyl (*S*)-4-(5-(5-bromo-3-(3-((*tert*-butyldiphenylsilyl)oxy)-2,2-dimethylpropyl)-1*H*-indol-2-yl)-6-(1-methoxyethyl)pyridin-3-yl)piperazine-1-carboxylate (146 g, 167.0 mmol) and Cs_2_CO_3_ (163.28 g, 501.1 mmol) in DMF (1200 mL) was added C_2_H_5_I (52.11 g, 334.0 mmol) in portions at 0 °C under N_2_ atmosphere. The final reaction mixture was stirred at 25 °C for 12 h. Desired product could be detected by LCMS. The resulting mixture was diluted with EA (1 L) and washed with brine (3 x 1.5 L). The organic layers were dried over anhydrous Na_2_SO_4_. After filtration, the filtrate was concentrated under reduced pressure to give benzyl (*S*)-4-(5-(5-bromo-3-(3-((*tert*-butyldiphenylsilyl)oxy)-2,2-dimethylpropyl)-1-ethyl-1*H*-indol-2-yl)-6-(1-methoxyethyl)pyridin-3-yl)piperazine-1-carboxylate (143 g, crude) as a yellow solid that was used directly for next step without further purification. ^1^H NMR (400 MHz, DMSO-*d*_6_) δ 8.48 (dd, *J* = 4.4, 2.8 Hz, 1H), 7.81 (dd, *J* = 4.2, 1.9 Hz, 1H), 7.59 – 7.46 (m, 5H), 7.45 – 7.34 (m, 10H), 7.33 – 7.22 (m, 3H), 5.13 (s, 2H), 4.12 – 3.63 (m, 3H), 3.52 (s, 4H), 3.42 (t, *J* = 9.2 Hz, 1H), 3.37 (s, 3H), 3.28 – 3.08 (m, 5H), 2.98 (s, 1H), 2.81 (s, 2H), 1.34 (d, *J* = 6.2 Hz, 2H), 1.21 (d, *J* = 6.3 Hz, 1H), 1.14 (t, *J* = 7.1 Hz, 2H), 1.07 (t, *J* = 7.1 Hz, 1H), 0.95 (d, *J* = 9.0 Hz, 9H), 0.77 (s, 1H), 0.69 (d, *J* = 3.9 Hz, 3H), 0.63 (s, 2H). ^13^C NMR (101 MHz, DMSO-*d*_6_) δ 154.81, 149.77, 145.24, 138.60, 137.24, 137.16, 136.69, 135.58, 135.52, 134.64, 133.48, 130.88, 130.12, 128.81, 128.29, 128.15, 128.08, 128.06, 126.01, 124.13, 122.48, 112.25, 112.12, 110.55, 75.32, 66.83, 56.04, 55.75, 47.87, 27.15, 27.13, 25.17, 25.03, 24.75, 19.37, 19.35, 19.03, 17.91, 15.63, 15.36. LCMS (ESI): m/z [M+H] calc’d for C_51_H_61_BrN_4_O_4_Si 900.4; found 901.4.

*benzyl (S)-4-(5-(5-bromo-1-ethyl-3-(3-hydroxy-2,2-dimethylpropyl)-1H-indol-2-yl)-6-(1-methoxyethyl)pyridin-3-yl)piperazine-1-carboxylate* (**T5**) To a stirred mixture of benzyl benzyl (*S*)-4-(5-(5-bromo-3-(3-((*tert*-butyldiphenylsilyl)oxy)-2,2-dimethylpropyl)-1-ethyl-1*H*-indol-2-yl)-6-(1-methoxyethyl)pyridin-3-yl)piperazine-1-carboxylate (143 g, 158.5 mmol) in DMF (1250 mL) was added CsF (72.24 g, 475.5 mmol). Then the reaction mixture was stirred at 60 °C for 2 days under N_2_ atmosphere. Desired product could be detected by LCMS. The resulting mixture was diluted with EA (1 L) and washed with brine (3 x 1L). Then the organic phase was concentrated under reduced pressure. The residue was purified by silica gel column chromatography, eluted with PE/EA (1/3) to afford two atropisomers of benzyl (*S*)-4-(5-(5-bromo-1-ethyl-3-(3-hydroxy-2,2-dimethylpropyl)-1*H*-indol-2-yl)-6-(1-methoxyethyl)pyridin-3-yl)piperazine-1-carboxylate **A** (38 g, 36% yield, RT = 1.677 min in 3 min LCMS(0.1% FA)) and **B** (34 g, 34% yield, RT = 1.578 min in 3 min LCMS(0.1% FA)) both as yellow solid. ^1^H NMR (400 MHz, DMSO-d6) δ 8.47 (d, J = 2.8 Hz, 1H), 7.90 (d, J = 1.9 Hz, 1H), 7.55 – 7.42 (m, 2H), 7.41 – 7.24 (m, 6H), 5.12 (s, 2H), 4.11 – 4.01 (m, 1H), 3.86 (m, 1H), 3.56 (d, J = 6.2 Hz, 4H), 3.33 (q, J = 5.0 Hz, 4H), 3.06 (d, J = 10.3 Hz, 1H), 2.98 (d, J = 10.3 Hz, 1H), 2.89 (s, 3H), 2.66 (d, J = 14.0 Hz, 1H), 2.51 (m, 1H), 2.15 (d, J = 14.1 Hz, 1H), 1.37 (d, J = 6.2 Hz, 3H), 1.16 (m, 4H), 0.63 (d, J = 8.3 Hz, 6H). ^13^C NMR (101 MHz, DMSO-d6) δ 154.89, 147.86, 145.90, 137.25, 136.12, 135.68, 134.69, 130.97, 128.84, 128.29, 128.11, 128.04, 124.36, 122.81, 112.28, 112.17, 111.73, 74.76, 70.20, 66.86, 56.06, 47.66, 38.20, 25.37, 25.18, 18.39, 15.35. LCMS (ESI): m/z [M+H] calc’d for C_35_H_43_BrN_4_O_4_ 663.2; found 662.2.

*(S)-4-(5-(1-ethyl-3-(3-hydroxy-2,2-dimethylpropyl)-5-(4,4,5,5-tetramethyl-1,3,2-dioxaborolan-2-yl)-1H-indol-2-yl)-6-(1-methoxyethyl)pyridin-3-yl)piperazine-1-carboxylate* (**T6**) Into a 500-mL 3-necked round-bottom flask purged and maintained with an inert atmosphere of nitrogen, was placed benzyl (*S*)-4-(5-(5-bromo-1-ethyl-3-(3-hydroxy-2,2-dimethylpropyl)-1*H*-indol-2-yl)-6-(1-methoxyethyl)pyridin-3-yl)piperazine-1-carboxylate **A** (14 g, 21.1 mmol), bis(pinacolato)diboron (5.89 g, 23.21 mmol), Pd(dppf)Cl_2_ (1.54 g, 2.1 mmol), KOAc (5.18 g, 52.7 mmol), Toluene (150 mL). The resulting solution was stirred for 5 h at 90 °C in an oil bath. The reaction mixture was cooled to 25 °C. The resulting mixture was concentrated under vacuum. The residue was purified by silica gel column chromatography, eluted with PE/EA (1/3) to give benzyl (*S*)-4-(5-(1-ethyl-3-(3-hydroxy-2,2-dimethylpropyl)-5-(4,4,5,5-tetramethyl-1,3,2-dioxaborolan-2-yl)-1*H*-indol-2-yl)-6-(1-methoxyethyl)pyridin-3-yl)piperazine-1-carboxylate (12 g, 76.0% yield) as a yellow solid. ^1^H NMR (400 MHz, DMSO-*d*_6_) δ 8.48 (d, *J* = 2.6 Hz, 1H), 8.09 (s, 1H), 7.56 (d, *J* = 8.2 Hz, 1H), 7.49 – 7.21 (m, 7H), 5.10 (s, 2H), 4.48 (m, 1H), 4.08 – 4.03 (m, 1H), 3.87 (m, 1H), 3.55 (d, *J* = 16.8 Hz, 10H), 3.34 – 3.19 (m, 4H), 3.07 (m, 2H), 2.82 (d, *J* = 13.8 Hz, 3H), 2.72 (d, *J* = 13.4 Hz, 1H), 2.26 (t, *J* = 15.0 Hz, 1H), 1.95 (s, 1H), 1.52 – 1.33 (m, 3H), 1.33 – 1.19 (m, 8H), 1.09 (s, 1H), 0.66 (dd, *J* = 30.5, 13.4 Hz, 6H). ^13^C NMR (101 MHz, DMSO-*d*_6_) δ 154.90, 149.97, 145.37, 138.41, 137.96, 137.24, 135.68, 128.96, 128.84, 128.29, 128.03, 111.81, 83.52, 75.30, 71.24, 66.84, 60.21, 55.74, 48.06, 38.33, 25.36, 25.23, 25.14, 25.07, 21.11, 18.02, 15.37, 15.29, 14.47. LCMS (ESI): m/z [M+H] calc’d for C_41_H_55_BN_4_O_6_ 710.4; found 711.3.

*(S)-1-((S)-3-(4-(2-(5-(4-((benzyloxy)carbonyl)piperazin-1-yl)-2-((S)-1-methoxyethyl)pyridin-3-yl)-1-ethyl-3-(3-hydroxy-2,2-dimethylpropyl)-1H-indol-5-yl)thiazol-2-yl)-2-((tert-butoxycarbonyl)amino)propanoyl)hexahydropyridazine-3-carboxylate* (**T7**) Into a 250-mL round-bottom flask purged and maintained with an inert atmosphere of argon, was placed benzyl (*S*)-4-(5-(1-ethyl-3-(3-hydroxy-2,2-dimethylpropyl)-5-(4,4,5,5-tetramethyl-1,3,2-dioxaborolan-2-yl)-1*H*-indol-2-yl)-6-(1-methoxyethyl)pyridin-3-yl)piperazine-1-carboxylate (10.8 g, 15.2 mmol), methyl (3*S*)-1-[(2*S*)-3-(4-bromo-1,3-thiazol-2-yl)-2-[(*tert*-butoxycarbonyl)amino]propanoyl]-1,2-diazinane-3-carboxylate (7.98 g, 16.7 mmol), Pd(dtbpf)Cl_2_ (0.99 g, 1.52 mmol), K_3_PO_4_ (8.06 g, 37.9 mmol), Toluene (60 mL), dioxane (20 mL), H_2_O (20 mL). The resulting solution was stirred for 3 h at 70 °C in an oil bath. The reaction mixture was cooled to 25 °C. The resulting solution was extracted with EtOAc (2 x 50 mL) and concentrated under reduced pressure. The residue was applied onto a silica gel column with ethyl acetate/hexane (10:1). Removal of solvent to give methyl (*S*)-1-((*S*)-3-(4-(2-(5-(4-((benzyloxy)carbonyl)piperazin-1-yl)-2-((*S*)-1-methoxyethyl)pyridin-3-yl)-1-ethyl-3-(3-hydroxy-2,2-dimethylpropyl)-1*H*-indol-5-yl)thiazol-2-yl)-2-(*(tert*-butoxycarbonyl)amino)propanoyl)hexahydropyridazine-3-carboxylate (8 g, 50.9% yield) as a yellow solid. ^1^H NMR (400 MHz, DMSO-*d*_6_) δ 8.48 (d, *J* = 2.8 Hz, 1H), 8.22 (d, *J* = 1.6 Hz, 1H), 7.84 – 7.67 (m, 2H), 7.52 (d, *J* = 8.6 Hz, 1H), 7.42 – 7.27 (m, 6H), 6.62 (d, *J* = 8.7 Hz, 1H), 5.46 – 5.24 (m, 2H), 5.12 (s, 2H), 4.45 (t, *J* = 5.1 Hz, 1H), 4.16 – 3.98 (m, 2H), 3.91 (m, 2H), 3.61 (d, *J* = 25.6 Hz, 8H), 3.29 (m, 5H), 3.11 (dd, *J* = 10.2, 5.3 Hz, 1H), 3.02 (dd, *J* = 10.2, 5.2 Hz, 1H), 2.86 (s, 3H), 2.70 (d, *J* = 14.0 Hz, 1H), 2.51 (p, *J* = 1.8 Hz, 1H), 2.26 (d, *J* = 14.0 Hz, 1H), 1.95 – 1.80 (m, 1H), 1.77 – 1.46 (m, 3H), 1.38 (d, *J* = 5.1 Hz, 12H), 1.16 (dd, *J* = 8.4, 5.9 Hz, 4H), 0.66 (d, *J* = 22.3 Hz, 6H). ^13^C NMR (101 MHz, DMSO-*d*_6_) δ 171.94, 171.33, 166.01, 154.90, 149.90, 145.40, 138.39, 137.28, 136.25, 135.82, 129.48, 128.87, 128.32, 128.07, 127.40, 126.50, 126.30, 120.70, 111.81, 110.23, 78.62, 75.32, 70.98, 66.85, 58.34, 55.76, 52.17, 48.04, 41.31, 38.36, 28.65, 25.27, 25.21, 18.03, 15.45. LCMS (ESI): m/z [M+H] calc’d for C_52_H_68_N_8_O_9_S 980.5; found 980.9.

*(S)-1-((S)-3-(4-(2-(5-(4-((benzyloxy)carbonyl)piperazin-1-yl)-2-((S)-1-methoxyethyl)pyridin-3-yl)-1-ethyl-3-(3-hydroxy-2,2-dimethylpropyl)-1H-indol-5-yl)thiazol-2-yl)-2-((tert-butoxycarbonyl)amino)propanoyl)hexahydropyridazine-3-carboxylic acid* (**T8**) To a stirred mixture of methyl (*S*)-1-((*S*)-3-(4-(2-(5-(4-((benzyloxy)carbonyl)piperazin-1-yl)-2-((S)-1-methoxyethyl)pyridin-3-yl)-1-ethyl-3-(3-hydroxy-2,2-dimethylpropyl)-1*H*-indol-5-yl)thiazol-2-yl)-2-((*tert*-butoxycarbonyl)amino)propanoyl)hexahydropyridazine-3-carboxylate (12 g, 12.23 mmol) in THF (100 mL)/H_2_O (100 mL) was added LiOH (2.45 g, 61.1 mmol) under N_2_ atmosphere and the resulting mixture was stirred for 2 h at 25 °C. Desired product could be detected by LCMS. THF was concentrated under reduced pressure. The pH of aqueous phase was acidified to 5 with HCL (1N) at 0 °C. The aqueous layer was extracted with DCM (3 x 100ml). The organic phase was concentrated under reduced pressure to give (*S*)-1-((*S*)-3-(4-(2-(5-(4-((benzyloxy)carbonyl)piperazin-1-yl)-2-((*S*)-1-methoxyethyl)pyridin-3-yl)-1-ethyl-3-(3-hydroxy-2,2-dimethylpropyl)-1*H*-indol-5-yl)thiazol-2-yl)-2-((*tert*-butoxycarbonyl)amino)propanoyl)hexahydropyridazine-3-carboxylic acid (10 g, 84.5% yield) as a light yellow solid. ^1^H NMR (400 MHz, DMSO-*d*_6_) δ 8.47 (d, *J* = 2.7 Hz, 1H), 8.29 (s, 1H), 7.75 (d, *J* = 8.7 Hz, 2H), 7.59 – 7.18 (m, 8H), 5.23 (s, 1H), 5.11 (s, 2H), 4.63 (d, *J* = 11.4 Hz, 1H), 4.20 (d, *J* = 11.4 Hz, 1H), 4.06 (m, 3H), 3.89 (m, 2H), 3.62 – 3.44 (m, 12H), 3.12 (d, *J* = 10.1 Hz, 1H), 3.02 (d, *J* = 10.3 Hz, 2H), 2.86 (d, *J* = 6.7 Hz, 3H), 2.70 (d, *J* = 16.6 Hz, 2H), 2.25 (d, *J* = 13.8 Hz, 1H), 1.96 (s, 1H), 1.71 (s, 2H), 1.28 – 0.96 (m, 11H), 0.65 (d, *J* = 21.1 Hz, 9H). ^13^C NMR (101 MHz, DMSO-*d*_6_) δ 165.66, 155.92, 155.24, 154.92, 149.87, 145.37, 137.23, 136.13, 135.75, 128.89, 128.35, 128.05, 111.91, 78.80, 75.28, 70.86, 66.84, 55.79, 47.99, 28.63, 25.31, 25.22, 18.09, 15.45. LCMS (ESI): m/z [M+H] calc’d for C_51_H_66_N_8_O_9_S 966.5; found 967.0.

*benzyl 4-(5-((6^3^S,4S,Z)-4-((tert-butoxycarbonyl)amino)-1^1^-ethyl-10,10-dimethyl-5,7-dioxo-6^1^,6^2^,6^3^,6^4^,6^5^,6^6^-hexahydro-1^1^H-8-oxa-2(4,2)-thiazola-1(5,3)-indola-6(1,3)-pyridazinacycloundecaphane-1^2^-yl)-6-((S)-1-methoxyethyl)pyridin-3-yl)piperazine-1-carboxylate* (**T9**) Into a 3-L round-bottom flask purged and maintained with an inert atmosphere of nitrogen, was placed (*S*)-1-((*S*)-3-(4-(2-(5-(4-((benzyloxy)carbonyl)piperazin-1-yl)-2-((*S*)-1-methoxyethyl)pyridin-3-yl)-1-ethyl-3-(3-hydroxy-2,2-dimethylpropyl)-1*H*-indol-5-yl)thiazol-2-yl)-2-((*tert*-butoxycarbonyl)amino)propanoyl)hexahydropyridazine-3-carboxylic acid (18 g, 18.61 mmol), ACN (1.8 L), DIEA (96.21 g, 744.4 mmol), EDCI (107.03 g, 558.3 mmol), HOBT (25.15 g, 186.1 mmol). The resulting solution was stirred for overnight at 25 °C. The resulting mixture was concentrated under reduced pressure after reaction completed. The resulting solution was diluted with DCM (1 L). The resulting mixture was washed with HCl (3 x 1 L, 1N aqueous). The resulting mixture was washed with water (3 x 1 L). Then the organic layer was concentrated, the residue was applied onto a silica gel column with ethyl acetate/hexane (1:1). Removal of solvent under reduced pressure gave benzyl 4-(5-((6^3^*S*,4*S*,*Z*)-4-((*tert*-butoxycarbonyl)amino)-1^1^-ethyl-10,10-dimethyl-5,7-dioxo-6^1^,6^2^,6^3^,6^4^,6^5^,6^6^-hexahydro-1^1^*H*-8-oxa-2(4,2)-thiazola-1(5,3)-indola-6(1,3)-pyridazinacycloundecaphane-1^2^-yl)-6-((*S*)-1-methoxyethyl)pyridin-3-yl)piperazine-1-carboxylate (10.4 g, 54.8% yield) as a light yellow solid. ^1^H NMR (400 MHz, DMSO-*d*_6_) δ 8.50 (d, *J* = 1.7 Hz, 1H), 8.47 (d, *J* = 2.8 Hz, 1H), 7.80 (s, 1H), 7.74 (dd, *J* = 8.6, 1.6 Hz, 1H), 7.55 (d, *J* = 8.7 Hz, 1H), 7.41 – 7.35 (m, 4H), 7.35 – 7.22 (m, 3H), 5.24 (t, *J* = 8.7 Hz, 1H), 5.11 (s, 2H), 5.06 (d, *J* = 12.2 Hz, 1H), 4.22 (m, 5H), 3.56 (t, *J* = 7.9 Hz, 6H), 3.29 (t, *J* = 5.3 Hz, 4H), 3.22 (s, 3H), 2.95 (d, *J* = 14.3 Hz, 1H), 2.77 (m, 1H), 2.50 (m, 1H), 2.44 (d, *J* = 14.4 Hz, 1H), 2.08 (t, *J* = 9.2 Hz, 1H), 1.80 (d, *J* = 9.0 Hz, 2H), 1.40 (s, 9H), 1.34 (d, *J* = 6.2 Hz, 4H), 1.08 (s, 1H), 0.95 – 0.84 (m, 6H), 0.35 (s, 3H). ^13^C NMR (101 MHz, DMSO-*d*_6_) δ 172.32, 167.04, 155.84, 154.90, 154.62, 149.67, 145.94, 137.44, 137.27, 135.52, 130.20, 128.86, 128.31, 128.06, 127.22, 126.38, 111.60, 78.50, 66.84, 55.82, 47.93, 35.69, 28.70, 26.25, 24.89, 16.49, 15.33. LCMS (ESI): m/z [M+H] calc’d for C_51_H_64_N_8_O_8_S 948.5; found 949.3.

*tert-butyl ((6^3^S,4S,Z)-11-ethyl-1^2^-(2-((S)-1-methoxyethyl)-5-(piperazin-1-yl)pyridin-3-yl)-10,10-dimethyl-5,7-dioxo-6^1^,6^2^,6^3^,6^4^,6^5^,6^6^-hexahydro-1^1^H-8-oxa-2(4,2)-thiazola-1(5,3)-indola-6(1,3)-pyridazinacycloundecaphane-4-yl)carbamate* (**T10**) Into a 250-mL round-bottom flask purged and maintained with an inert atmosphere of nitrogen, was placed benzyl 4-(5-((6^3^*S*,4*S*,*Z*)-4-((*tert*-butoxycarbonyl)amino)-1^1^-ethyl-10,10-dimethyl-5,7-dioxo-6^1^,6^2^,6^3^,6^4^,6^5^,6^6^-hexahydro-1^1^*H*-8-oxa-2(4,2)-thiazola-1(5,3)-indola-6(1,3)-pyridazinacycloundecaphane-1^2^-yl)-6-((*S*)-1-methoxyethyl)pyridin-3-yl)piperazine-1-carboxylate (10.40 g, 10.9 mmol), Pd(OH)_2_/C (5 g, 46.9 mmol), MeOH (100 mL). The resulting solution was stirred for 3 h at 25 °C under 2 atm H_2_ atmosphere. The solids were filtered out and the filter cake was washed with MeOH (3 x 100 mL). Then combined organic phase was concentrated under reduced pressure to give *tert*-butyl ((6^3^*S*,4*S*,*Z*)-1*1*-ethyl-1^2^-(2-((*S*)-1-methoxyethyl)-5-(piperazin-1-yl)pyridin-3-yl)-10,10-dimethyl-5,7-dioxo-6^1^,6^2^,6^3^,6^4^,6^5^,6^6^-hexahydro-1^1^*H*-8-oxa-2(4,2)-thiazola-1(5,3)-indola-6(1,3)-pyridazinacycloundecaphane-4-yl)carbamate (8.5 g, 90.4% yield) as a light yellow solid. ^1^H NMR (400 MHz, DMSO-*d*_6_) δ 8.54 (d, *J* = 1.7 Hz, 1H), 8.49 (dd, *J* = 8.8, 2.9 Hz, 1H), 7.84 (s, 1H), 7.77 (dd, *J* = 8.6, 1.6 Hz, 1H), 7.59 (d, *J* = 8.7 Hz, 1H), 7.33 (d, *J* = 9.2 Hz, 1H), 7.21 (d, *J* = 2.9 Hz, 1H), 5.79 (s, 1H), 5.28 (t, *J* = 9.0 Hz, 1H), 5.10 (d, *J* = 12.2 Hz, 1H), 4.44 – 4.05 (m, 6H), 3.63 (t, *J* = 7.7 Hz, 3H), 3.49 – 3.07 (m, 13H), 3.06 – 2.94 (m, 2H), 2.89 (dd, *J* = 6.3, 4.0 Hz, 3H), 2.81 (t, *J* = 13.7 Hz, 1H), 2.66 (s, 1H), 2.54 (m, 1H), 2.49 (d, *J* = 14.4 Hz, 1H), 2.13 (d, *J* = 14.4 Hz, 1H), 1.83 (s, 2H), 1.66 – 1.50 (m, 1H), 1.44 (s, 8H), 1.38 (d, *J* = 6.1 Hz, 4H), 1.12 (s, 1H), 0.95 (d, *J* = 7.1 Hz, 6H), 0.40 (s, 3H). ^13^C NMR (101 MHz, DMSO-*d*_6_) δ 173.07, 172.31, 167.02, 155.84, 154.63, 146.59, 137.62, 135.50, 130.22, 127.12, 124.89, 119.57, 111.51, 111.27, 78.47, 55.78, 55.33, 48.82, 45.53, 35.68, 28.72, 26.27, 24.90, 16.50, 15.34. LCMS (ESI): m/z [M+H] calc’d for C_43_H_58_N_8_O_6_S 814.4; found 815.3.

*tert-butyl ((6^3^S,4S,Z)-1^1^-ethyl-1^2^-(2-((S)-1-methoxyethyl)-5-(4-methylpiperazin-1-yl)pyridin-3-yl)-10,10-dimethyl-5,7-dioxo-6^1^,6^2^,6^3^,6^4^,6^5^,6^6^-hexahydro-1^1^H-8-oxa-2(4,2)-thiazola-1(5,3)-indola-6(1,3)-pyridazinacycloundecaphane-4-yl)carbamate* (**T11**) A solution of *tert*-butyl ((6^3^*S*,4*S*,*Z*)-1*1*-ethyl-1^2^-(2-((*S*)-1-methoxyethyl)-5-(piperazin-1-yl)pyridin-3-yl)-10,10-dimethyl-5,7-dioxo-6^1^,6^2^,6^3^,6^4^,6^5^,6^6^-hexahydro-1^1^*H*-8-oxa-2(4,2)-thiazola-1(5,3)-indola-6(1,3)-pyridazinacycloundecaphane-4-yl)carbamate (1.5 g, 1.58 mmol, 1 equiv), Pd(OH)_2_/C (1.5 g) and Paraformaldehyde (208.85 mg, 4.74 mmol, 3 equiv) in MeOH (15 mL) was stirred for 2 h at 25 °C under hydrogen atmosphere. The resulting mixture was filtered, the filter cake was washed with methanol (3 x 50 mL). The filtrate was concentrated under reduced pressure. The residue was purified by reversed-phase flash chromatography with the following conditions: column, C18 silica gel; mobile phase, MeCN in water (10 mmol/L NH_4_HCO_3_), 10 % to 70 % gradient in 30 min; detector, UV 254 nm. This resulted in tert-butyl ((6^3^S,4S,Z)-1^1^-ethyl-1^2^-(2-((S)-1-methoxyethyl)-5-(4-methylpiperazin-1-yl)pyridin-3-yl)-10,10-dimethyl-5,7-dioxo-6^1^,6^2^,6^3^,6^4^,6^5^,6^6^-hexahydro-1^1^H-8-oxa-2(4,2)-thiazola-1(5,3)-indola-6(1,3)-pyridazinacycloundecaphane-4-yl)carbamate (800 mg, 58.01 % yield) as a yellow solid. ^1^H NMR (400 MHz, DMSO-*d*_6_) δ 8.37 – 8.15 (m, 2H), 7.61 – 7.43 (m, 2H), 7.31 (d, *J* = 8.6 Hz, 1H), 7.12 – 6.90 (m, 2H), 5.17 – 4.96 (m, 1H), 4.85 (d, *J* = 11.8 Hz, 1H), 4.22 – 3.86 (m, 5H), 3.39 (q, *J* = 11.7 Hz, 3H), 3.16 – 2.89 (m, 9H), 2.77 (d, *J* = 14.1 Hz, 1H), 2.57 (d, *J* = 17.4 Hz, 1H), 2.36 – 2.09 (m, 5H), 1.98 (s, 3H), 1.91 – 1.81 (m, 1H), 1.66 – 1.44 (m, 2H), 1.17 (d, *J* = 17.7 Hz, 12H), 0.70 (d, *J* = 8.5 Hz, 6H), 0.16 (s, 3H). ^13^C NMR (101 MHz, DMSO-*d*_6_) δ 173.04, 172.29, 167.00, 155.81, 154.63, 149.06, 146.09, 137.59, 135.50, 130.21, 127.12, 126.35, 111.52, 78.45, 60.19, 55.76, 54.64, 47.87, 46.24, 35.68, 28.71, 26.26, 24.89, 16.49, 15.34, 14.53. LCMS (ESI): m/z [M+H] calc’d for C_44_H_60_N_8_O_6_S 828.4; found 829.3.

*(6^3^S,4S,Z)-4-amino-1^1^-ethyl-1^2^-(2-((S)-1-methoxyethyl)-5-(4-methylpiperazin-1-yl)pyridin-3-yl)-10,10-dimethyl-6^1^,6^2^,6^3^,6^4^,6^5^,6^6^-hexahydro-1^1^H-8-oxa-2(4,2)-thiazola-1(5,3)-indola-6(1,3)-pyridazinacycloundecaphane-5,7-dione* (**T12**) A solution of tert-butyl ((6^3^*S*,4*S*,*Z*)-1^1^-ethyl-1^2^-(2-((*S*)-1-methoxyethyl)-5-(4-methylpiperazin-1-yl)pyridin-3-yl)-10,10-dimethyl-5,7-dioxo-6^1^,6^2^,6^3^,6^4^,6^5^,6^6^-hexahydro-1^1^*H*-8-oxa-2(4,2)-thiazola-1(5,3)-indola-6(1,3)-pyridazinacycloundecaphane-4-yl)carbamate (800 mg, 0.97 mmol, 1 equiv) in DCM (10 mL) was treated with TFA (2 mL) at 0 °C. After 3 min, the resulting solution was warmed to 25 °C and then stirred for 2 h. The mixture was then basified to pH 7 with saturated NaHCO_3_ (aq.). The resulting mixture was extracted with DCM (3 x 50 mL) and dried over anhydrous Na_2_SO_4_. After filtration, the filtrate was concentrated under reduced pressure to afford (6^3^*S*,4*S*,*Z*)-4-amino-1^1^-ethyl-1^2^-(2-((*S*)-1-methoxyethyl)-5-(4-methylpiperazin-1-yl)pyridin-3-yl)-10,10-dimethyl-6^1^,6^2^,6^3^,6^4^,6^5^,6^6^-hexahydro-1^1^*H*-8-oxa-2(4,2)-thiazola-1(5,3)-indola-6(1,3)-pyridazinacycloundecaphane-5,7-dione (680 mg, crude) as a yellow solid. ^1^H NMR (400 MHz, DMSO-*d*_6_) δ 8.48 (dd, *J* = 18.2, 2.2 Hz, 2H), 7.81 – 7.65 (m, 2H), 7.54 (d, *J* = 8.6 Hz, 1H), 7.23 (d, *J* = 2.9 Hz, 1H), 4.93 (d, *J* = 12.1 Hz, 1H), 4.38 (d, *J* = 8.5 Hz, 1H), 4.29 (p, *J* = 7.3, 5.9 Hz, 2H), 4.21 – 4.09 (m, 3H), 4.03 (q, *J* = 7.1 Hz, 1H), 3.60 (q, *J* = 10.9 Hz, 2H), 3.21 (s, 3H), 2.97 (d, *J* = 14.5 Hz, 1H), 2.75 (m, 2H), 2.54 – 2.37 (m, 6H), 2.21 (s, 3H), 2.08 (d, *J* = 12.1 Hz, 1H), 1.99 (s, 2H), 1.88 – 1.64 (m, 2H), 1.51 (m, 1H), 1.35 (d, *J* = 6.1 Hz, 3H), 1.23 (s, 1H), 1.18 (t, *J* = 7.1 Hz, 2H), 0.97 – 0.85 (m, 6H), 0.36 (s, 3H). ^13^C NMR (101 MHz, DMSO-*d*_6_) δ 176.35, 172.38, 168.21, 154.46, 148.98, 146.08, 137.48, 135.44, 130.17, 127.17, 126.50, 125.00, 119.62, 117.54, 111.54, 110.90, 110.75, 75.73, 70.80, 57.85, 55.73, 54.62, 51.37, 47.83, 46.23, 35.73, 26.15, 24.91, 16.50, 15.35. LCMS (ESI): m/z [M+H] calc’d for C_39_H_52_N_8_O_4_S 728; found 729.

*(1S,2S)-N-((6^3^S,4S,Z)-1^1^-ethyl-1^2^-(2-((S)-1-methoxyethyl)-5-(4-methylpiperazin-1-yl)pyridin-3-yl)-10,10-dimethyl-5,7-dioxo-6^1^,6^2^,6^3^,6^4^,6^5^,6^6^-hexahydro-1^1^H-8-oxa-2(4,2)-thiazola-1(5,3)-indola-6(1,3)-pyridazinacycloundecaphane-4-yl)-2-methylcyclopropane-1-carboxamide* (**RMC-6236**) To a stirred solution of (6^3^*S*,4*S*,*Z*)-4-amino-1^1^-ethyl-1^2^-(2-((*S*)-1-methoxyethyl)-5-(4-methylpiperazin-1-yl)pyridin-3-yl)-10,10-dimethyl-6^1^,6^2^,6^3^,6^4^,6^5^,6^6^-hexahydro-1^1^*H*-8-oxa-2(4,2)-thiazola-1(5,3)-indola-6(1,3)-pyridazinacycloundecaphane-5,7-dione (400 mg, 0.55 mmol, 1 equiv) and (1S,2S)-2-methylcyclopropane-1-carboxylic acid (65.92 mg, 0.66 mmol, 1.2 equiv) in DMF (4 mL) was added DIEA (283.7 mg, 2.2 mmol, 4 equiv) and HATU (229.51 mg, 0.60 mmol, 1.1 equiv) in portions at 0 °C under nitrogen atmosphere. The resulting mixture was stirred for 40 min at 0 °C then the reaction was quenched by the addition of sat. NH_4_Cl (aq.) (30 mL). The resulting mixture was extracted with EtOAc (3 x 10 mL). The combined organic layers were washed with brine (2 x 50 mL) and dried over anhydrous Na_2_SO_4_. After filtration, the filtrate was concentrated under reduced pressure. The resulting crude product was purified by Prep-HPLC with the following conditions (Column: C18 spherical, 20-35 um; 100 A, 80 g; Mobile Phase A:Water(10 MMOL/L NH_4_HCO_3_), Mobile Phase B:ACN; Flow rate:60 mL/min; Gradient:30 B to 70 B in 30 min, 254 nm; RT1:15.2) to afford (1*S*,2*S*)-*N*-((6^3^*S*,4*S*,*Z*)-1^1^-ethyl-1^2^-(2-((*S*)-1-methoxyethyl)-5-(4-methylpiperazin-1-yl)pyridin-3-yl)-10,10-dimethyl-5,7-dioxo-6^1^,6^2^,6^3^,6^4^,6^5^,6^6^-hexahydro-1^1^*H*-8-oxa-2(4,2)-thiazola-1(5,3)-indola-6(1,3)-pyridazinacycloundecaphane-4-yl)-2-methylcyclopropane-1-carboxamide (102 mg, 96.4% purity, 22.0% yield) as a white solid. ^1^H NMR (400 MHz, DMSO-d6) δ 8.26 (d, J = 22.0 Hz, 3H), 7.50 (d, J = 18.5 Hz, 2H), 7.29 (d, J = 8.5 Hz, 1H), 6.98 (s, 1H), 5.37 (t, J = 9.2 Hz, 1H), 4.84 (d, J = 11.7 Hz, 1H), 4.16 – 3.84 (m, 5H), 3.15 (s, 2H), 3.01 (d, J = 18.9 Hz, 9H), 2.74 (d, J = 14.0 Hz, 1H), 2.23 (d, J = 20.8 Hz, 5H), 1.96 (s, 3H), 1.86 (s, 1H), 1.55 (s, 2H), 1.29 (s, 2H), 1.21 – 1.01 (m, 3H), 0.84 (s, 4H), 0.68 (s, 8H), 0.31 (s, 1H), 0.14 (s, 3H). 13C NMR (100 MHz, DMSO) δ 172.78, 172.32, 166.74, 154.72, 149.04, 146.09, 137.56, 135.49, 130.18, 127.12, 126.32, 125.00, 119.59, 117.45, 111.51, 111.36, 110.86, 75.72, 70.73, 57.89, 55.81, 54.65, 49.42, 47.87, 46.28, 38.24, 35.69, 33.00, 28.94, 26.23, 24.92, 23.47, 22.58, 21.16, 18.08, 16.54, 15.37, 15.09. HRMS (ESI): m/z [M+H] calc’d for C_44_H_58_N_8_O_5_S 811.43073.; found 811.43236.

Synthesis of Intermediate 1

*(S)-3-(4-bromothiazol-2-yl)-2-((tert-butoxycarbonyl)amino)propanoic acid* (**I1**) To a solution of methyl (2*S*)-3-(4-bromo-1,3-thiazol-2-yl)-2-[(*tert*-butoxycarbonyl)amino]propanoate (110 g, 301.2 mmol) in THF (500 mL) and H_2_O (200 mL) at room temperature was added LiOH (21.64 g, 903.6 mmol). The solution was stirred for 1 h and was then concentrated under reduced pressure. The residue was adjusted to pH 6 with 1 M HCl and then extracted with DCM (3 x 500 mL). The combined organic layers were, dried over Na_2_SO_4_, filtered, and concentrated under reduced pressure to give (*S*)-3-(4-bromothiazol-2-yl)-2-((*tert*-butoxycarbonyl)amino)propanoic acid (108 g, crude). ^1^H NMR (400 MHz, DMSO-d6) δ 7.70 (s, 1H), 7.24 (d, J = 8.6 Hz, 1H), 4.28 (m, 1H), 3.44 (dd, J = 15.0, 4.5 Hz, 1H), 3.27 (dd, J = 14.9, 9.8 Hz, 1H), 1.36 (s, 9H). ^13^C NMR (101 MHz, DMSO-d6) δ 172.81, 168.56, 155.73, 123.40, 118.64, 78.85, 53.76, 34.86, 28.58. LCMS (ESI): m/z [M+H] calc’d for C_11_H_16_BrN_2_O_4_S 351.0; found 351.0.

*(S)-1-((S)-3-(4-bromothiazol-2-yl)-2-((tert-butoxycarbonyl)amino)propanoyl)hexahydropyridazine-3-carboxylate* (**Intermediate 1**) To a solution of (*S*)-3-(4-bromothiazol-2-yl)-2-((*tert*-butoxycarbonyl)amino)propanoic acid (70 g, 199.3 mmol) in DCM (500 mL) at 0 °C was added methyl (3*S*)-1,2-diazinane-3-carboxylate bis(trifluoroacetic acid) salt (111.28 g, 298.96 mmol), NMM (219.12 mL, 1993.0 mmol), EDCI (76.41 g, 398.6 mmol) and HOBt (5.39 g, 39.89 mmol). The solution was warmed to room temperature and stirred for 1 h. The reaction was then quenched with H_2_O (500 mL) and was extracted with EtOAc (3 x 500 mL). The combined organic layers were dried over Na_2_SO_4_, filtered, and concentrated under reduced pressure. The residue was purified by silica gel column chromatography to give methyl (*S*)-1-((*S*)-3-(4-bromothiazol-2-yl)-2-((*tert*-butoxycarbonyl)amino)propanoyl)hexahydropyridazine-3-carboxylate (88.1 g, 93% yield). ^1^H NMR (400 MHz, DMSO-d6) δ 7.69 (s, 1H), 6.66 (d, J = 8.9 Hz, 1H), 5.34 (d, J = 9.5 Hz, 1H), 5.24 (m, 1H), 3.85 – 3.71 (m, 1H), 3.67 (s, 3H), 3.60 (q, J = 8.7 Hz, 1H), 3.27 (d, J = 4.0 Hz, 1H), 3.13 (dd, J = 14.7, 8.8 Hz, 2H), 1.96 – 1.78 (m, 1H), 1.68 (m, 2H), 1.57 (m, 1H), 1.35 (s, 9H). ^13^C NMR (101 MHz, DMSO-d6) δ 171.90, 171.19, 168.68, 155.22, 123.23, 118.79, 78.57, 58.14, 52.20, 50.94, 41.23, 35.91, 28.61, 22.52. LCMS (ESI): m/z [M+H] calc’d for C_17_H_26_BrN_4_O_5_S 477.1; found 477.1.


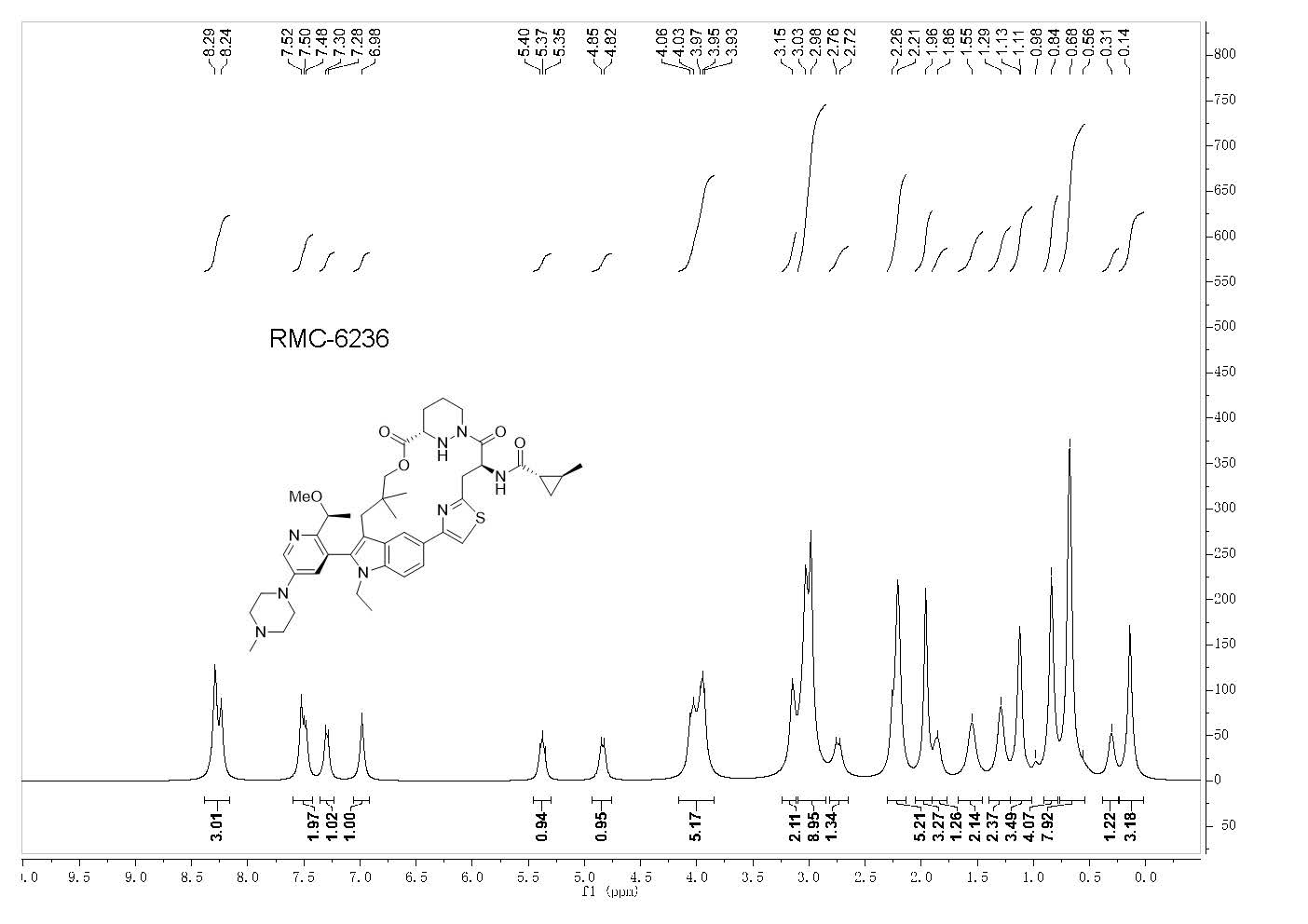


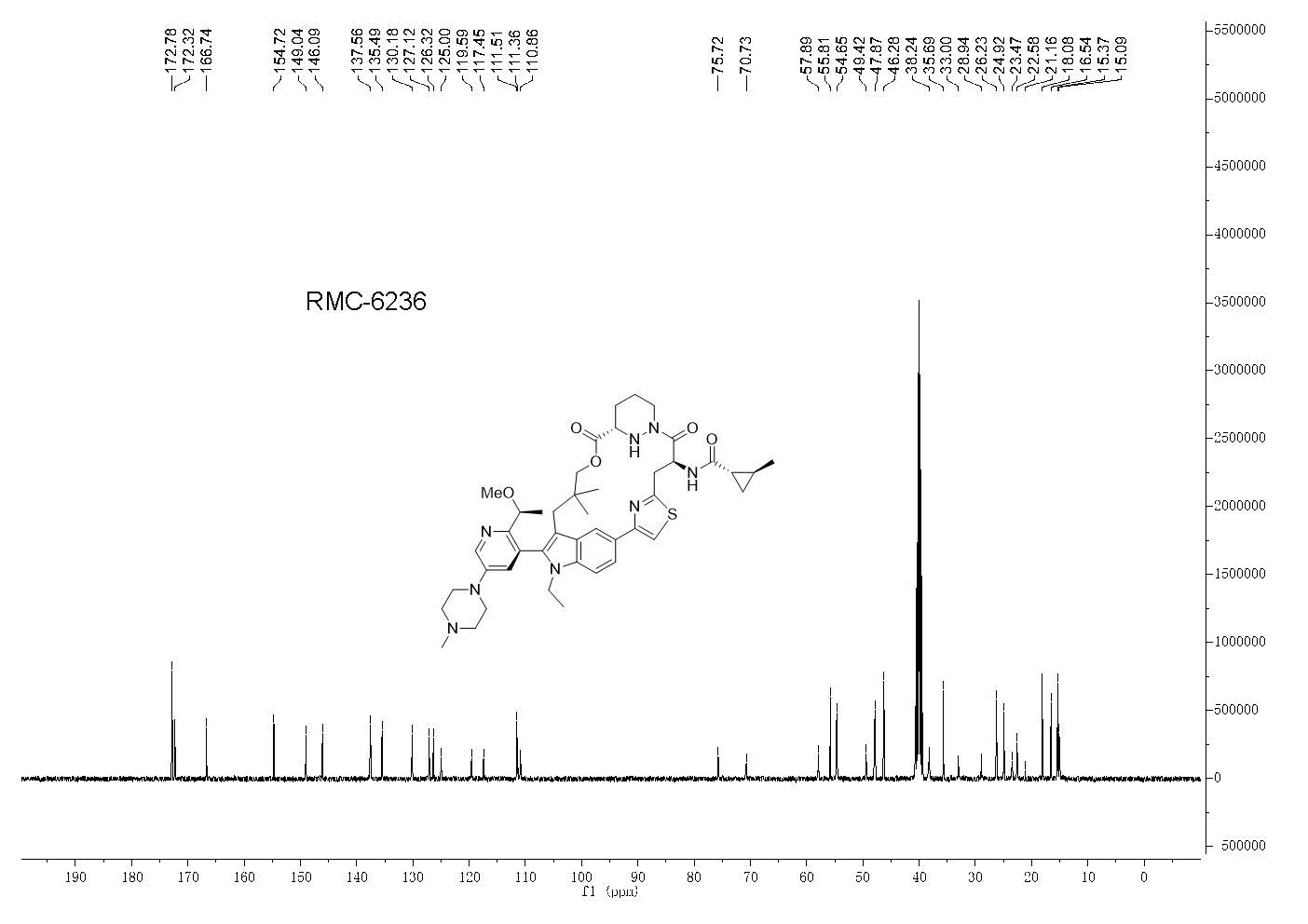


**PK/PD/Efficacy Modeling**

All modeling was conducted using Phoenix WinNonlin version 8.3.5.340.

PK/efficacy modeling was conducted by fitting blood PK to a one-compartment model and the whole blood concentration was used to drive tumor growth inhibition in a tumor model based on the Simeoni et al approach. The tumor growth inhibition model incorporates exponential and linear growth phases for intrinsic tumor growth and a concentration-dependent drug-mediated tumor killing effect. Intrinsic tumor growth parameters include initial tumor volume, W_0_, which was set to the observed initial tumor volume, and the exponential and linear growth rates constants, λ_0_ and λ_1_, which were estimated from vehicle-treated animal tumor growth and fixed. Drug-specific parameters include a measure of drug potency, k_2_, and a transfer rate constant, k_1_, which describes cell death kinetics. Estimates of k_2_ were dose-dependent and were inversely related to dose, leading to underestimation of drug potency at dose levels that saturate cell killing. As such, the parameters estimated from data at the 10 mg/kg dose level were used for translational modeling since this dose level leads to shallow regression but does not saturate the cell-killing effect. A tumor stasis concentration threshold (C_T_) was calculated as λ_0_/k_2_.

Tumor PK and PD were added to the model by incorporating blood-plasma partitioning, plasma protein binding, and tumor partitioning and PD parameters. RMC-6236 exhibits concentration-dependent blood-plasma partitioning across species so a non-linear function was fitted to in vitro whole blood-plasma partitioning data as shown in Equation 1.

Equation 1.

$$B:P={B:P}_{0}-(\frac{I_{max}*C_{B}}{{IC}_{50}+C_{B}})$$

Where B:P is the blood:plasma partitioning ratio at a specific blood concentration, B:P_0_ is the fitted blood:plasma partitioning ratio at zero concentration, I_max_ is the maximal decrease in blood:plasma partitioning, and IC_50_ is the half-maximal concentration in blood to achieve the lowest blood:plasma partitioning ratio.

Plasma protein binding did not exhibit concentration-dependence, so a constant value (98.9% and 97.7% in mice and humans) was applied to calculate unbound plasma concentrations. The unbound plasma concentrations were used as a driving function to predict total tumor concentrations. Separate tumor partitioning rates were fit to match the observed extended half-life of RMC-6236. The tumor partitioning rates were manually adjusted to best fit repeat dose data as this was assumed to be more relevant for clinical predictions. It is important to note that the kinetic rate constant for removal of drug from the tumor incorporates a non-identifiable tumor fraction unbound variable, since only unbound drug should partition across cell membranes.

The ordinary differential equations for the PK/Efficacy and PK/PD models are listed below. All parameter estimates are listed in Supplemental Table 7.

**Mouse PK-Efficacy Model:**

dAa/dt = -Ka * Aa

dA1/dt = Ka * Aa – Cl * C

dX1/dt = ((lambda0 * X1)/(1+((W_tot_ * (lambda0/lambda1))^Psi^))^1/Psi^ – K2 * C * X1

dX2/dt = K2 * C * X1 – K1 * X2

dX3/dt = K1 * X2 – K1 * X3

dX4/dt = K1 * X3 – K1 * X4

where:

C = A1/V

W_tot_ = X1 + X2 + X3 + X4

RMC-6236 dose administered at Aa and W_0_ administered as X1 at t=0

**Mouse PK/PD Model:**

dAa/dt = -Ka * Aa

dA1/dt = Ka * Aa – Cl * C

dCt/dt = K_pt_ * fu * (C/BP) – K_tb_ * C_t_

dE/dt = K_in_ * (1 – I_max_*C_t_/(C_t_ + IC_50_)) – K_out_*E

where:

C = A1/V

fu = 0.011

BP = 5.5 – ((5.2*C)/(1819+C))

K_in_ = Baseline*K_out_

Baseline = 100

RMC-6236 dose administered at Aa

**Human PK/PD Model:**

dAa/dt = -Ka * Aa

dA1/dt = Ka * Aa – Cl * C

dCt/dt = K_pt_ * fu * (C/BP) – K_tb_ * C_t_

dE/dt = K_in_ * (1 – I_max_*C_t_/(C_t_ + IC_50_)) – K_out_*E

where:

C = A1/V

fu = 0.023

BP = 5.7 – ((5.2*C)/(963+C))

K_in_ = Baseline*K_out_

Baseline = 100

RMC-6236 dose administered at Aa
