# Supplementary material for: Translational and Therapeutic Evaluation of RAS-GTP Inhibition by RMC-6236 in RAS-Driven Cancers
Source: Cancer Discov. 2024 Apr 9;14(6):994–1017. doi: 10.1158/2159-8290.CD-24-0027 (PMC11149917; doi:10.1158/2159-8290.CD-24-0027)
Supplement: Supplementary Figure S1-S6 — Supplementary Figure 1 shows RMC-6236 crystal structure in tri-complex, as well as biophysical and cellular potencies of RMC-6236 by genotype.Supplementary Figure 2 shows RMC-6236 demonstrates dose-dependent anti-tumor activities at tolerable doses; and pharmacodynamic effects on RAS signaling in NCI-H441 xenograft tumors as assessed by IHC, and in relatively refractory KP-4 and NCI-H2122 xenograft tumors as assessed by human DUSP6 mRNA expression in vivo.Supplementary Figure 3 shows genotype dependent response of RMC-6236 across NSCLC, PDAC and CRC; and potential modifiers to the durability of response of KRASG12C NSCLC models upon RMC-6236 treatment assessed by Kaplan-Meier analyses.Supplementary Figure 4 shows Efficacy of RMC-6236 on KrasG12C–driven autochthonous lung tumors harboring cis second-site mutations within KrasG12C (KrasG12C,H95D or KrasG12C,Y96C) and eCT26 (KrasG12D/G12D) syngeneic model in immunocompetent mice; anti-tumor immunity of RMC-6236; and in intracranially implanted NCI-H1373-Luc xenograft model on nude mice.Supplementary Figure 5 shows effects of RMC-6236 mediated pharmacological modulation in KP-4 xenograft tumors and normal colon tissues isolated from xenograft tumor bearing mice.Supplementary Figure 6 shows a graphical representation of the combined mouse PK-Efficacy and PK/PD model. [file cd-24-0027_supplementary_figure_s1-s6_suppsf1.docx]

**Supplementary Figures**

**Supplementary Fig. S1**


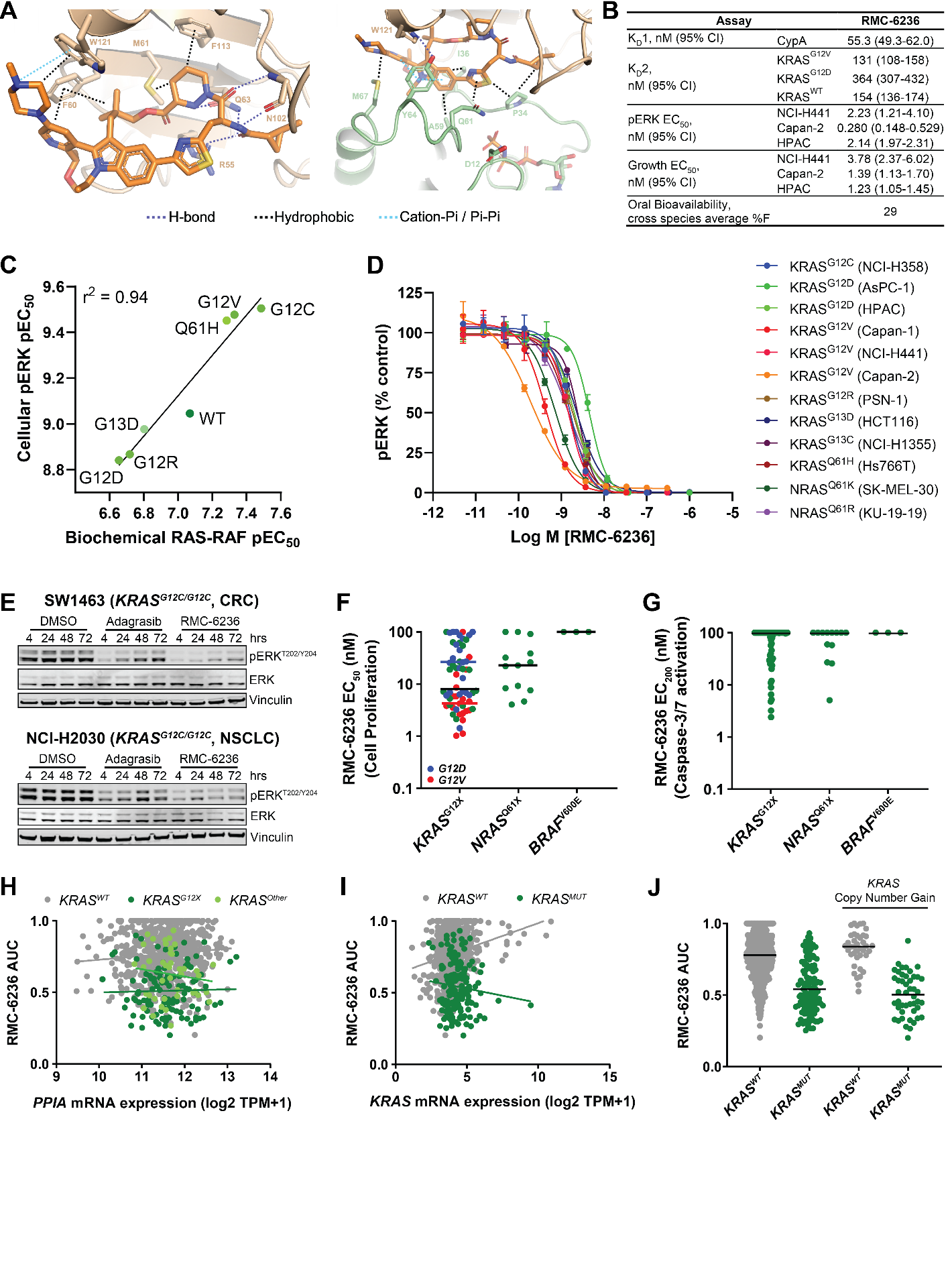


**Supplementary Figure S1**. RMC-6236 crystal structure and sensitivity by genotype. **A,** A high-resolution co-crystal structure of RMC-6236 bound to CypA and GMPPNP-bound KRAS^G12D^**.** Left panel, RMC-6236 binds CypA in its active site where it forms hydrophobic contacts with F60, M61, F113, and W121. The left-hand side piperidine of RMC-6236 forms a cation-pi interaction with the aromatic sidechain of W121. RMC-6236 forms hydrogen bonds with the sidechain of R55 and Q63 as well as the backbone of N102. Right panel, in the tri-complex, RMC-6236 forms numerous, mostly hydrophobic, interactions at the RAS SWI/SWII groove. The core indole moiety forms a large contact area with I36 on SWI and Y64 on SWII. CypA W121 donates a hydrogen bond to RAS Y64 and the acidic SWI region of RAS forms salt bridges with a basic region on CypA as previously described (not pictured). Mutant residue D12 forms no direct contacts with either RMC-6236 or CypA.  **B,** Potencies for RMC-6236 in biophysical (K_D_1 is an average of 18 replicate experiments measured by Surface Plasmon Resonance (SPR) and K_D_2 is an average of 8 replicate experiments measured by BioLayer Interferometry (BLI)), pERK measured by MesoScale Discovery (MSD) Assays, proliferation measured by CellTiter-Glo Cell Viability Assay (CTG), Oral bioavailability measured by LC-MS/MS analysis. **C,** Correlation between cellular (RAS-less MEFs) and biochemical potencies (pEC_50_ = -log_10_ of EC_50_ in moles/liter) of RMC-6236 for different RAS variants (R-squared value = 0.94), with. **D,** Cellular pERK inhibition potencies of RMC-6236 in cancer cell lines harboring indicated *KRAS* or *NRAS* mutations. pERK measured by AlphaLISA assays. Data shown are representative of 1-20 replicate experiments. **E,** Immunoblot protein Western analyses of pERK in SW1463 (*KRAS^G12C/G12C^*, CRC) and NCI-H2030 (*KRAS^G12C/G12C^*, NSCLC) cancer cells treated with DMSO, 1000nM Adagrasib, or 100nM of RMC-6236 at the indicated time points. **F,** RMC-6236 EC_50_ potency in *KRAS^G12X^*, *NRAS^Q61X^* and *BRAF^V600E^* mutant cell lines in a 5-day 3D methylcellulose CTG assay*.*In the *KRAS^G12X^* column, 19 *KRAS^G12V^* cell lines were plotted in red (median EC_50_ = 4.2 nM), and 23 *KRAS^G12D^* cell lines were blue (median EC_50_ = 26.2 nM). **G,** RMC-6236 EC_200_ (concentration at 2-fold induction) values measured in a 5-day caspase 3/7 activation assay. **H,** Scatter plot of *PPIA* mRNA expression (log2 TPM +1) and RMC-6236 potency (AUC) measured in the PRISM assay. *KRAS^G12X^* mutant cells shown in green, cells with other *KRAS* mutations (*KRAS^Other^*) shown in light-green, wild-type *KRAS* cells shown in gray. R squared values for *KRAS^G12X^*, *KRAS^Other^*, and wild-type *KRAS* were 0.00068, 0.018 and 0.0083, respectively. **I,** Scatter plot of *KRAS* mRNA expression (log2 TPM +1) and RMC-6236 potency (AUC) measured in the PRISM assay. *KRAS* mutant (*KRAS^MUT^*) cells shown in green, wild-type *KRAS* cells shown in gray. R squared values for *KRAS^MUT^* and wild-type *KRAS* were 0.00602 and 0.0146, respectively. **J,** RMC-6236 potency measured in the PRISM panel of cancer cell lines. Median AUC values for wild-type *KRAS*, *KRAS^MUT^*, wild-type copy number gained *KRAS*, and mutant copy number gained *KRAS* were 0.78, 0.57, 0.84, and 0.54, respectively. *KRAS* gene copy number gain was classified based on a copy number z-score being 2 or greater.

**Supplementary Fig. S2**


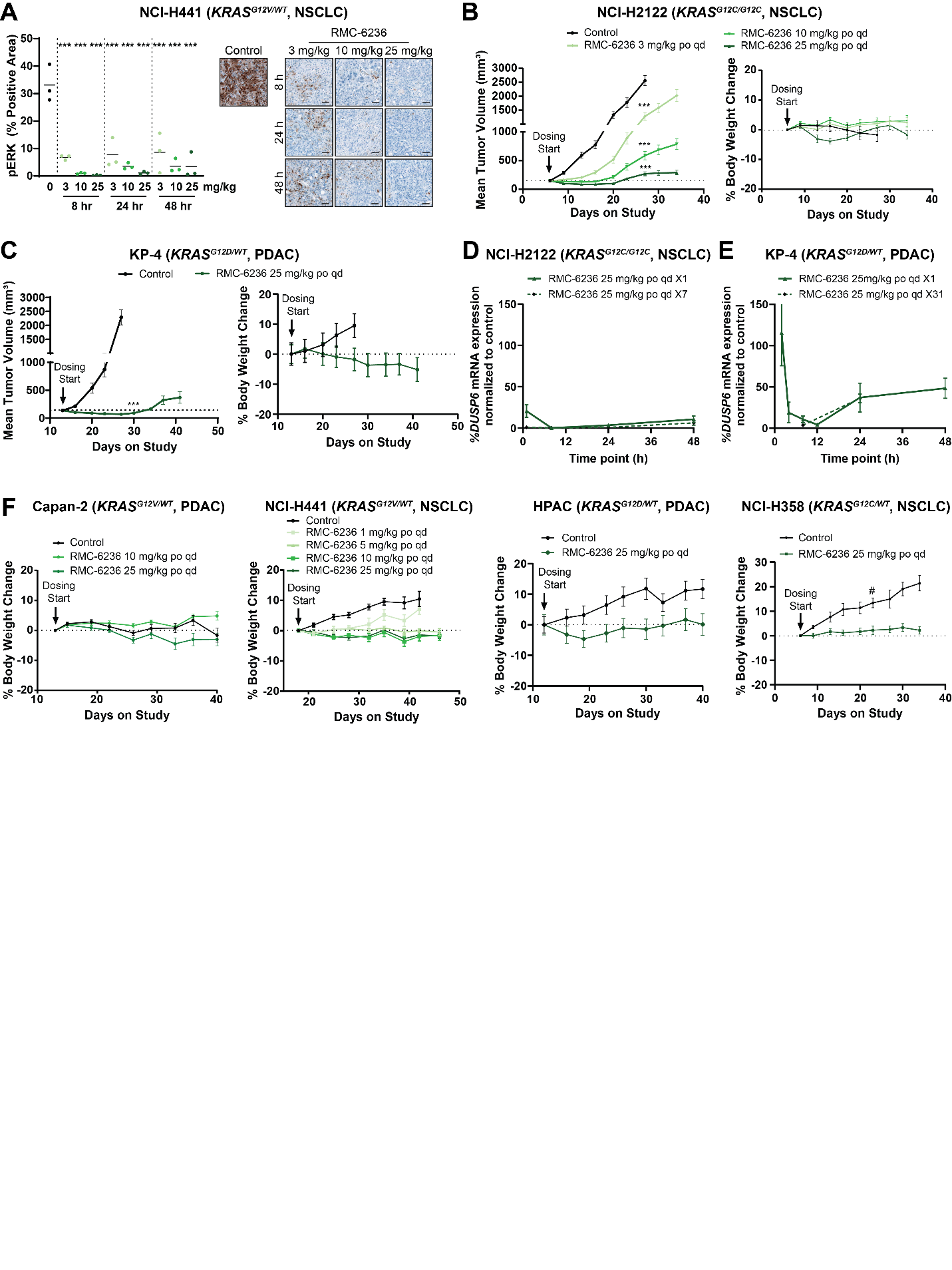


**Supplementary Figure S2.** RMC-6236 demonstrates dose-dependent antitumor activities at tolerable doses. **A,** Histopathology of NCI-H441 (*KRAS^G12V/WT^*, NSCLC) xenograft tumors treated with vehicle or single dose of RMC-6236 (3, 10, 25 mg/kg) and collected at indicated timepoints (n = 3/timepoint/dose). Staining of indicated markers in tumor areas was quantified and compared to vehicle using one-way ANOVA followed by Dunnett’s multiple comparison test (***p < 0.001). Representative images are shown at 200x magnification from samples closest to the mean of the respective groups. Scale bar is 50µm. **B-C,** Dose-dependent antitumor activity of RMC-6236 and % body weight change from baseline in subcutaneous xenograft models of (B) NCI-H2122 (*KRAS^G12C/G12C^*, NSCLC) (n = 8–15 per group) and (C) KP-4 (*KRAS^G12D/WT^*, PDAC) (n = 8 per group). Tumor-bearing mice were treated with vehicle or RMC-6236 at indicated doses for 14 to 28 days. Mean tumor volumes (left panels) or % body weight change (right panels) of each group were plotted over the course of treatment. Mean tumor volume of vehicle control and RMC-6236 groups were compared by 2-way repeated measures ANOVA on the last measurement day of the vehicle group (***p < 0.001). Dotted line indicates the initial average tumor volume (left panels) or % body weights change (right panels). Error bars indicate SEM. **D-E,** PD of RMC-6236 in (D) NCI-H2122 (*KRAS^G12C/G12C^*, NSCLC) and (E) KP-4 (*KRAS^G12D/WT^*, PDAC) xenograft tumors, shown as relative change in *DUSP6* mRNA expression. Tumor-bearing mice were treated with a single dose of vehicle or RMC-6236 at 25 mg/kg (solid lines); or 7 (D) or 31 (E) consecutive daily doses of RMC-6236 at 25 mg/kg (dashed lines). Values plotted as mean ± SEM.  **F,** % Body weight change from baseline in subcutaneous xenograft models of Capan-2 (*KRAS^G12V/WT^*, PDAC) (n = 8 per group), NCI-H441 (*KRAS^G12V/WT^*, NSCLC) (n = 10 per group), HPAC (*KRAS^G12D/WT^*, PDAC) (n = 10 per group) and NCI-H358 (*KRAS^G12C/WT^*, NSCLC) (n = 8–10 per group). Tumor-bearing mice were treated with vehicle or RMC-6236 at indicated doses for 27-28 days. Dotted line indicates the initial average body weight of each group. Error bars indicate SEM. # indicates 1 animal terminated due to tumor burden based on IACUC guidance.

**Supplementary Fig. S3**


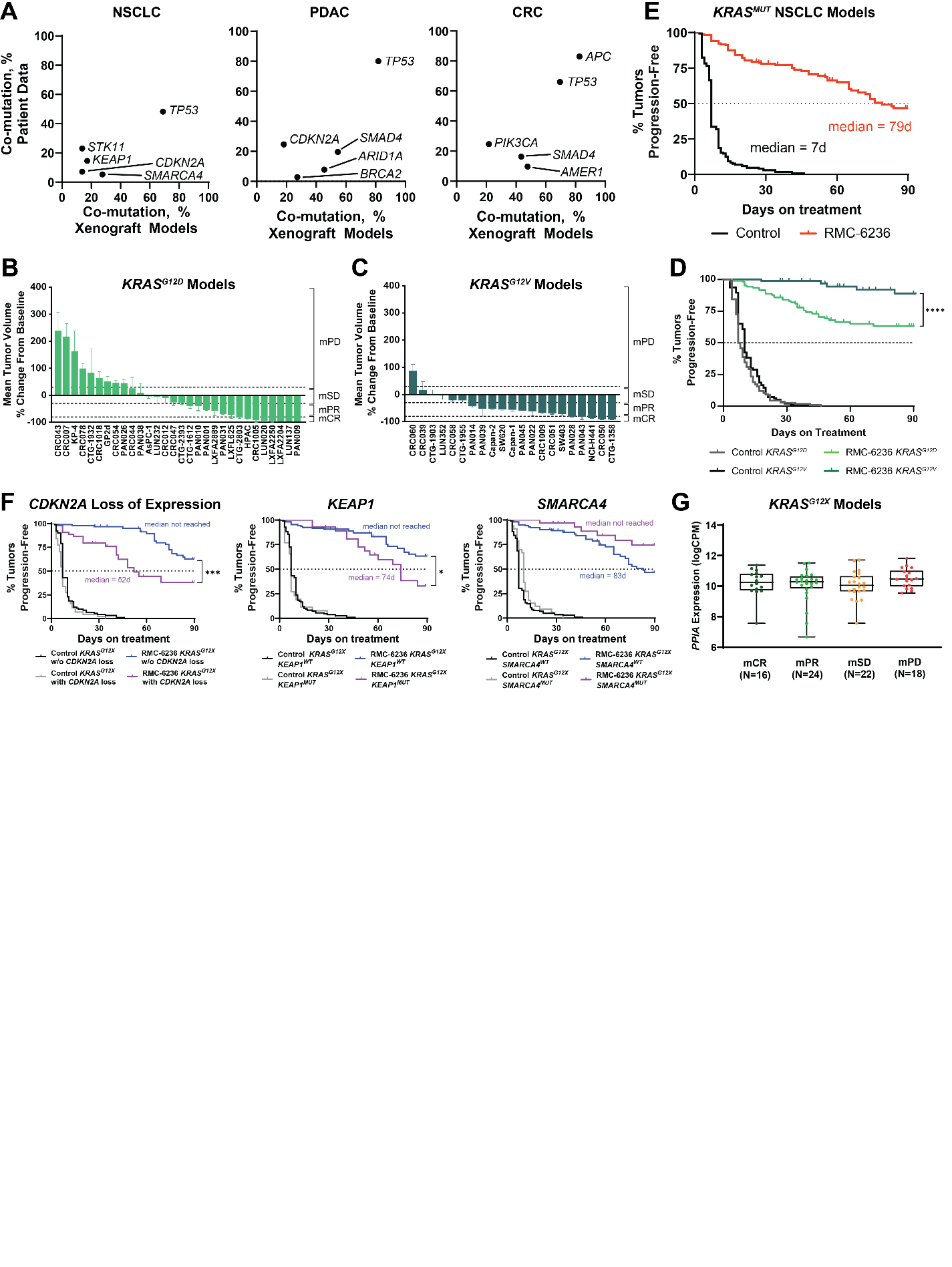


**Supplementary Figure S3.** Potential co-modifiers of response to RMC-6236 treatment. **A,** Correlation in the prevalence of co-occurring mutations in patients with *KRAS^G12X^* tumors and in the cohorts of NSCLC, PDAC and CRC xenograft models evaluated in this study. Patient data are from FMI (1). Only variants described as “oncogenic” or “likely oncogenic” were included. **B-C,** Tumor response waterfall plots of 30 *KRAS^G12D^* (B) and 21 *KRAS^G12V^* (C) xenograft models (n = 1–10 per model) upon RMC-6236 daily treatment at 25 mg/kg. Average % mean tumor volume change ± SEM from baseline at response calling date are shown. mRECIST criteria were used to call tumor response as indicated on right hand side of each waterfall plot. **D,** Kaplan-Meier analyses of time to tumor doubling on treatment in individual tumor-bearing animals from *KRAS^G12D^* (B) and *KRAS^G12V^* (C) xenograft models upon daily treatment of vehicle control or RMC-6236 at 25 mg/kg for up to 90 days. 30 *KRAS^G12D^* (n = 116 animals in Control, n = 106 in RMC-6236 treatment group), 21 *KRAS^G12V^* (n = 97 animals in Control, n = 90 in RMC-6236 treatment group) were included. Time to event was determined by the time on treatment until tumor volume doubling from baseline on survival plots by Kaplan-Meier analysis. Log-rank (Mantel-Cox) test and Cox Proportional Hazards model were used to compare predictive effect of *KRAS^G12V^* vs. *KRAS^G12D^* on PFS of RMC-6236 treated subjects. *KRAS^G12V^* models exhibited significantly longer PFS (log-rank ****p < 0.0001, Cox Proportional Hazard Ratio 0.178, 95% interval 0.069–0.454). **E,** Kaplan-Meier analysis of *KRAS^MUT^* NSCLC models upon daily treatment of vehicle control or RMC-6236 at 25 mg/kg for up to 90 days. 35 *KRAS^MUT^* NSCLC models (29 *KRAS^G12X^* and 6 *KRAS^Other^* models. n = 170 animals in Control, n = 169 animals in RMC-6236 treatment group) were included. Time to event was determined by the time on treatment until tumor size doubling from baseline on survival plots by Kaplan-Meier analysis. Log-rank test was used to compare vehicle control with treatment groups, Cox Proportional Hazards models were used to estimate Hazard Ratios: (HR 0.082, 95% interval 0.056-0.118, p < 2 × 10^−16^). **F,** Kaplan-Meier analysis of *KRAS^G12X^* NSCLC models stratified by indicated potential co-modifiers upon daily treatment of vehicle control or RMC-6236 at 25 mg/kg for up to 90 days. Time to event was determined as described above. 29 *KRAS^G12X^* NSCLC models were included in the analysis. Left panel, 10 models with *CDKN2A* loss (n = 44 in both Control and RMC-6236 treatment group) and 19 models without *CDKN2A* loss (n = 91 in both Control and RMC-6236 treatment group) were included. Middle panel, 5 *KEAP1^MUT^* models (n = 26 in Control, n = 28 in RMC-6236 treatment group) and 24 *KEAP1^WT^* models (n = 109 in Control, n = 107 in RMC-6236 treatment group) were included. Right panel, 8 *SMARCA4^MUT^* models (n = 33 in both Control and RMC-6236 treatment group) and 21 *SMARCA4^WT^* models (n = 102 in both Control and RMC-6236 treatment group) were included. Log-rank (Mantel-Cox) test and Cox Proportional Hazards model were used to compare predictive effect of indicated co-modifier on PFS of RMC-6236 treated subjects. *KRAS^G12X^* NSCLC models with *CDKN2A* loss exhibited significantly shorter PFS (log-rank ***p < 0.001, Cox Proportional Hazard Ratio 3.60, 95% interval 1.88–6.89) compared with those without *CDKN2A* loss. *KRAS^G12X^* NSCLC models with *KEAP1^MUT^* exhibited significantly shorter PFS (log-rank *p < 0.05, Cox Proportional Hazard Ratio 2.10, 95% interval 1.10–4.03) compared with *KEAP1^WT^* models. **G,** Bar plots of baseline *PPIA* mRNA levels in *KRAS^G12X^* xenograft models surveyed in MCT categorized by tumor response (mRECIST). Data from 2 models (OVCAR-5 and LXFA2889) were not available, hence not included in the plot.

**Supplementary Fig. S4**

**
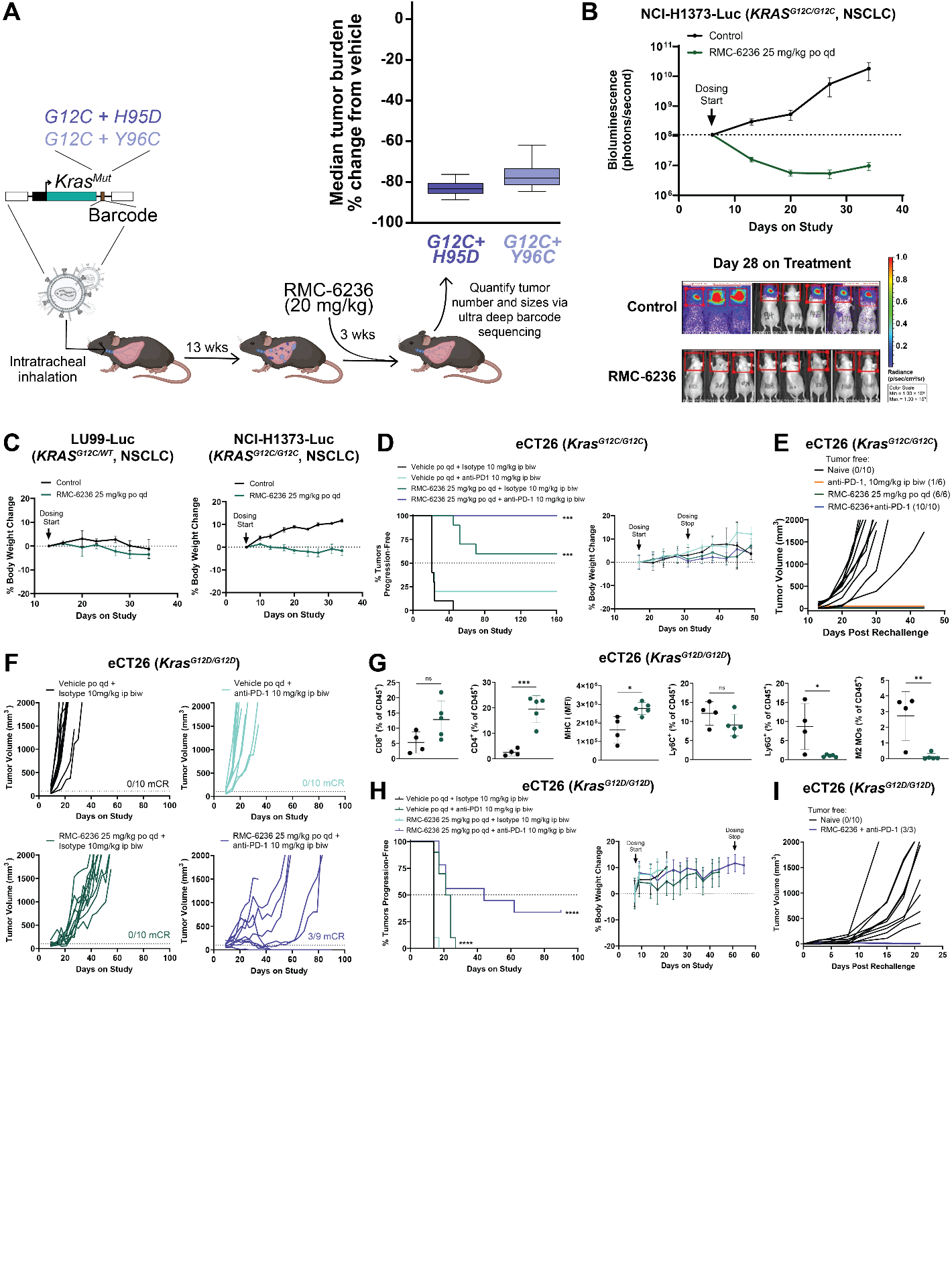
**

**Supplementary Figure S4.** Translating RMC-6236 activity in NSCLC. **A,** Efficacy of RMC-6236 on Kras^G12C^–driven autochthonous lung tumors harboring cis second-site mutations within *Kras^G12C^* (*Kras^G12C,H95D^* or *Kras^G12C,Y96C^*) in immunocompetent mice. A mix of lentiviral cDNA vectors encoding each oncogenic *Kras^G12C^* variant was delivered intratracheally to the lungs of each mouse, and 13 weeks post-tumor growth, mice were treated with RMC-6236 at 20 mg/kg po qd for 3 weeks prior to analysis. 95% confidence internals are shown. **B,** Efficacy of RMC-6236 in the intracranially implanted NCI-H1373-Luc xenograft model (n =8 per group). RMC-6236 was dosed at 25 mg/kg daily for 28 days. Images of bioluminescence in individual mice were shown. Results were shown as mean ± SEM. **C,** % Body weight change from baseline in intracranial xenograft models of LU99-Luc (n = 8 per group) and NCI-H1373-Luc (n = 8 per group). Tumor-bearing mice were treated daily with vehicle or RMC-6236 at 25 mg/kg for 21 days (Left) or 28 days (right). The dotted line indicates the initial average body weight of each group. Error bars indicate SEM. **D,** Treatment response durability (left) and mouse body weights (right) following repeated administration of RMC-6236 and the combination with anti-PD-1in BALB/c mice bearing the murine colon carcinoma eCT26 syngeneic tumors (*Kras^G12C/G12C^*) (n = 10 per group). Kaplan-Meier curve for progression-free survival analysis based on 14 days of treatment (130 days post treatment-stop, 161 days post implantation); Tumor doubling from the baseline was used as a surrogate for tumor progression. RMC-6236 and anti-PD-1 treatment started on day 17 post-implantation. RMC-6236 treatment was stopped at day 31 post-implantation and anti-PD-1 at day 35 post-implantation. Vehicle controls and treatment groups were compared by Log-rank (Mantel-Cox) test in Kaplan-Meier plot (***p < 0.001). **E,** Tumor specific immune response to the rechallenge implantation at day 130 post dosing stop (161 days post initial implantation) with eCT26 (*Kras^G12C/G12C^*) cells in mice with mCRs after treatment with RMC-6236 as single agent and in combination with anti‑PD-1, shown as individual spider plots. **F,** Antitumor activity of RMC-6236 and the combination with anti-PD-1 following repeated administration in BALB/c mice bearing the murine colon carcinoma eCT26 syngeneic model (*Kras^G12D/G12D^*) shown as individual tumor growth curves (n = 9-10 per group). Graphs indicate number of complete regressions per injected mice (mCR). RMC-6236 and anti-PD-1 treatment started at day 9 post-implantation. RMC-6236 treatment was stopped at day 51 post-implantation and anti-PD-1 at day 30 post-implantation. **G**, Immune cell composition (CD8+ and CD4+ T cells, Ly6C+ and Ly6G+ myeloid derived suppressor cells and M2 macrophages) in murine colon carcinoma eCT26 syngeneic model (*Kras^G12D/G12D^*) tumors represented as percentage of CD45+ cells and mean fluorescent intensity (MFI) of cell surface MHC I marker on viable, CD45- large cells (assessed as tumor cells) 24 hours post 8 days of treatment with vehicle (n = 4 biological replicates) or RMC-6236 at 25 mg/kg po qd (n = 5 biological replicates). *p<0.05; **p<0.01; ***p<0.001; ns, nonsignificant by two-sided Student’s test. **H,** Treatment response durability (right) and mouse body weights (left) following repeated administration of RMC-6236 and the combination with anti-PD-1in BALB/c mice bearing the murine colon carcinoma eCT26 syngeneic tumors (*Kras^G12D/G12D^*) (n = 9-10 per group). Kaplan-Meier curve for progression-free survival analysis based on 42 days of treatment (40 days post treatment-stop, 90 days post implantation); Tumor doubling from the baseline was used as a surrogate for tumor progression. RMC-6236 and anti-PD-1 treatment started at day 9 post-implantation. RMC-6236 treatment was stopped at day 51 post-implantation and anti-PD-1 at day 30 post-implantation. Vehicle controls and treatment groups were compared by Log-rank (Mantel-Cox) test in Kaplan-Meier plot (***p < 0.0001). **I,** Tumor specific immune response to the rechallenge implantation at day 42 post dosing stop (92 days post initial implantation) with eCT26 (*Kras^G12D/G12D^*) cells in mice with mCRs after treatment with RMC-6236 in combination with anti‑PD-1, shown as individual spider plots.

**Supplementary Fig. S5**


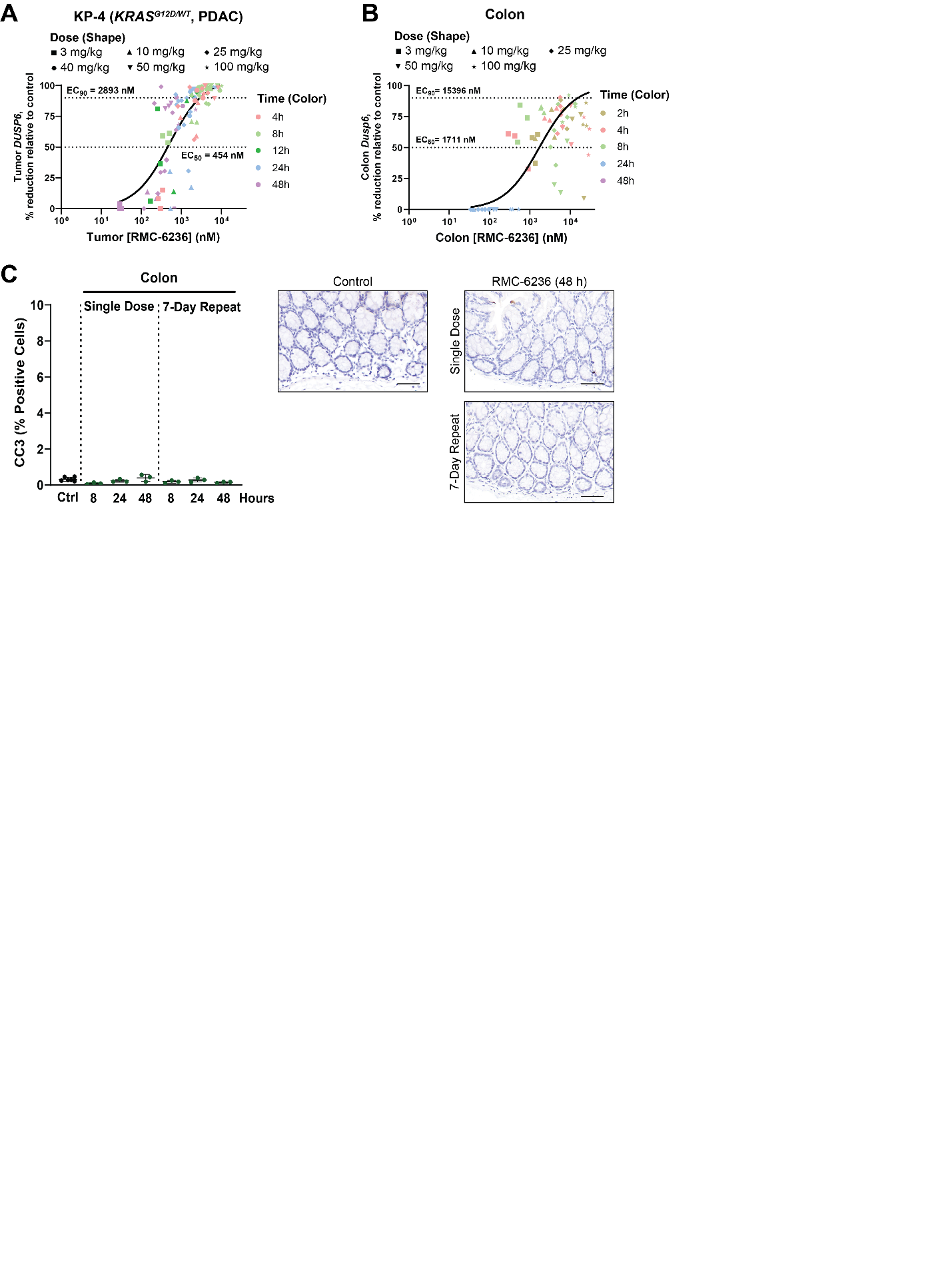


**Supplementary Figure S5.** Effects of RMC-6236 mediated pharmacological modulation in tumor-bearing mice. **A,** PK/PD relationship between RMC-6236 concentration and inhibition of *DUSP6* expression in KP-4 (EC_50_ = 454 nM and EC_90_ = 2893 nM) xenograft tumors. Subcutaneous xenograft tumors were treated with vehicle or RMC-6236 ranging from 3 mg/kg to 100 mg/kg. **B,** PK/PD relationship between RMC-6236 concentration and inhibition of *Dusp6* expression in colon (EC_50_ = 1711 nM and EC_90_ = 15,396 nM) isolated from tumor-bearing BALB/c nude mice treated with vehicle or RMC-6236 ranging from 3 mg/kg to 100 mg/kg. **A-B,** Tumors and colon from tumor-bearing BALB/c nude mice were harvested at indicated timepoints (n = 3/timepoint/dose). A 3-parameter sigmoidal exposure response model was fitted to the data to derive EC_50_ and EC_90_ values. Timepoints represented by colors and doses represented by symbol shapes. **C,** Histopathology of colon tissue from Capan-2 xenograft model collected at indicated timepoints post a single dose of vehicle control, RMC-6236 at 25 mg/kg or 7 consecutive daily doses of RMC-6236 at 25 mg/kg (n = 3–6/timepoint/dose). Staining of CC3 in colon was quantified. Representative images are shown at 200x magnification from samples closest to the mean of the respective groups. Scale bar is 50µm.

**Supplementary Fig. S6**


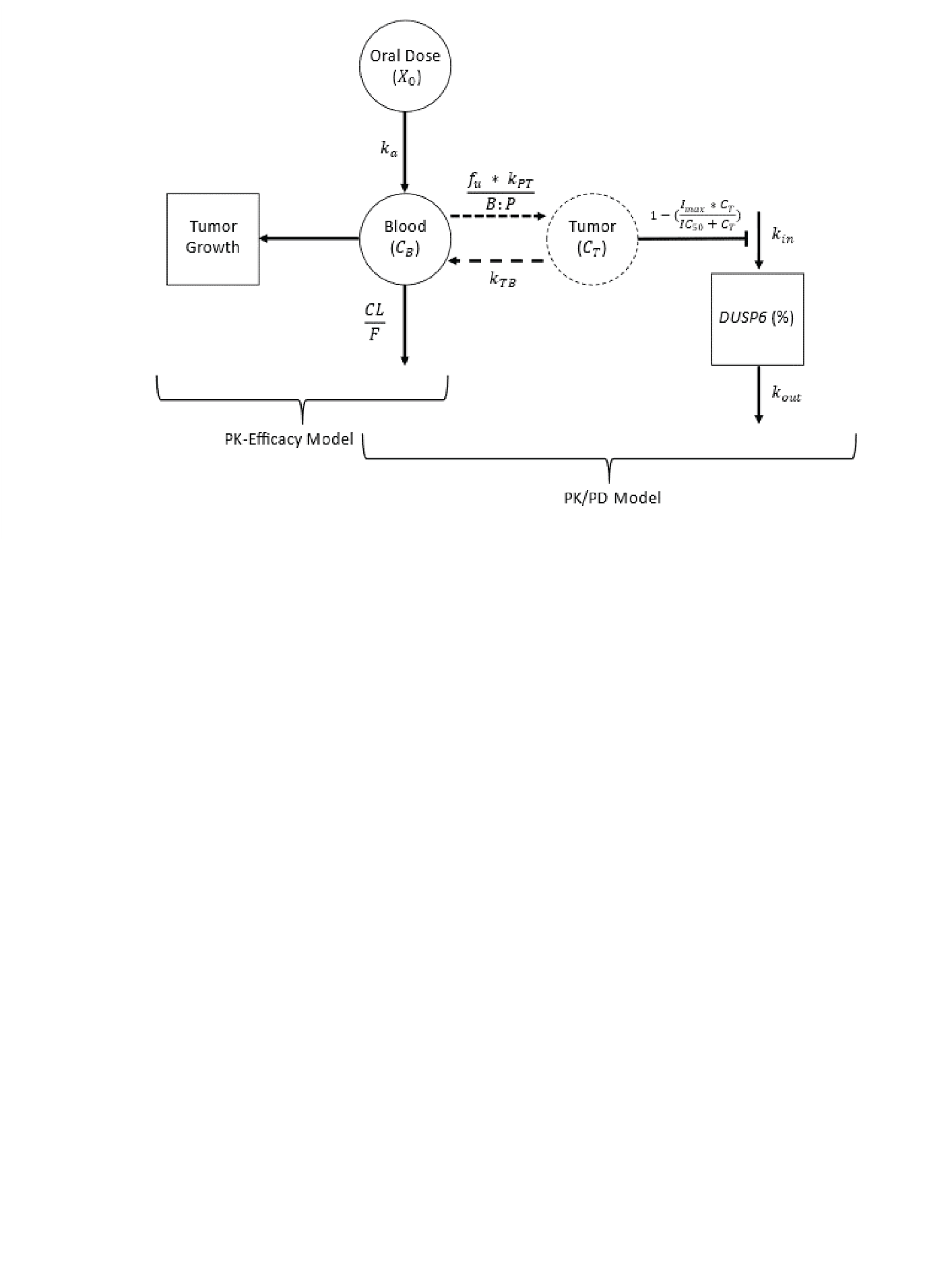


**Supplementary Figure S6.** A graphical representation of the combined mouse PK-Efficacy and PK/PD model. Whole blood pharmacokinetics were defined using a one compartment model and whole blood concentrations were used to drive inhibitory effects on tumor growth. Tumor concentrations were predicted from unbound plasma concentrations, corrected for concentration-dependent blood-plasma partitioning and plasma protein binding. *DUSP6* mRNA expression was used as the PD biomarker and was modeled via an indirect model driven by tumor PK.
